# When behavioural science can make a difference in times of COVID-19

**DOI:** 10.1017/bpp.2020.48

**Published:** 2020-09-01

**Authors:** DARIO KRPAN, FADI MAKKI, NABIL SALEH, SUZANNE IRIS BRINK, HELENA VLAHINJA KLAUZNICER

**Affiliations:** 1 London School of Economics and Political Science, Department of Psychological and Behavioural Science, London, UK; 2 B4Development Foundation, Doha, Qatar; 3 Nudge Lebanon, Beirut, Lebanon; 4 Independent Researcher, London, UK; 5 B4Development Foundation, Doha, Qatar

## Abstract

In a large study that involved 2637 participants recruited from a representative UK and US sample, we tested the influence of four behavioural interventions (versus control) on a range of behaviours important for reducing the spread of COVID-19 a day after the interventions were administered. Even if people largely complied with social distancing measures, our analyses showed that for certain subgroups of the population the interventions made a positive difference. More specifically, for those who started practising social distancing relatively recently, an information-based intervention increased general compliance with social distancing and reduced both the number of times people went out and the number of hours they spent outside. However, for people who started practising social distancing relatively early, the interventions tended to backfire and, in some cases, reduced compliance with social distancing. Overall, this research has various policy implications and shows that, although behavioural interventions can positively impact compliance with social distancing, their effect may depend on personal circumstances.

## Introduction

Starting in the Hubei province of China in December 2019, COVID-19 spread across the globe and had a major impact on the world in the following months. Many countries took unprecedented measures to curtail the pandemic and ‘flatten the curve’. They asked their citizens to wash their hands frequently, keep distance from others when outside and declared lockdowns, meaning that people had to stay at home unless undertaking essential activities, such as shopping for groceries and obtaining medicine. With this sudden need to convince millions of people to behave in ways radically different from their routine, governments started looking at behavioural science to encourage compliance with the newly introduced measures (e.g., Barari *et al.*, 2020; Gumber & Bulsari, 2020; Knight, 2020; Yates, 2020). At the same time, social and behavioural scientists wrote recommendations for policymakers concerning the interventions and scientific principles they should consider using (Brooks *et al.*, 2020; Hahn *et al.*, 2020a; Haushofer & Metcalf, 2020; Johnson *et al.*, 2020; Lunn *et al.*, 2020; Van Bavel *et al.*, 2020).

However, the results of the first controlled experiments deploying such interventions are mixed. Barari *et al.* (2020) investigated whether messages designed to evoke norms and prompt reflection about the social impacts of one's actions would improve attitudes and increase intentions to comply with governmental measures in Italy. They found no effect of the interventions compared to a control group. In another study, Blagov (2020) tested schema-congruence theory – according to which people prefer and are more easily persuaded by messages aligned with their own views of themselves – in the context of COVID-19. Participants rated the appeal of five public health messages directed at people with different personalities. Fewer than half of the hypotheses were supported: although certain personality traits predicted message appeal, these links were largely message non-specific. Moreover, in a wider study testing moral messages, Everett *et al.* (2020) employed the ‘messenger effect’ as one of the widely used behavioural science intervention techniques (Dolan *et al.*, 2012). They manipulated the messenger as being either a citizen (high school teacher) or a leader (Department of Education director), but did not find any meaningful effect on behavioural intentions. Finally, Pfattheicher *et al.* (2020) tested whether showing a short empathy-inducing video combined with information about the use of physical distancing would increase the motivation to adhere to the distancing compared to information alone or a control condition. In contrast to the other studies cited, they did find an impact of their intervention. The empathy and information condition increased respondents’ motivation to comply with physical distancing relative to both control and information alone.

Based on these mixed findings, are we to conclude that behavioural science interventions are not what they are cut out to be when push comes to shove, or that they only ‘sometimes work’? The reality is that there could be many explanations for the mixed results. However, one plausible explanation, as indicated by Barari *et al.*'s (2020) findings, is that most participants were already complying with the governmental measures and there may have been a limited space for the interventions to make a difference. This echoes Fetzer *et al.*'s (2020) findings, according to which, in a study covering 58 countries and over 100,000 respondents, many people took the pandemic seriously and complied with the governmental measures. Nevertheless, understanding how to influence those individuals who do not comply is crucial given that, even if they are not the majority, they can have a large impact on the spread of the virus (Liu *et al.*, 2020; Shereen *et al.*, 2020). Therefore, it is important to uncover which behavioural science interventions can further improve compliance with behaviours aimed at reducing the spread of COVID-19, and under which circumstances.

### Exploring the space for influence

In the present research, we aimed to further examine whether, and under which conditions, behavioural science can make a difference concerning the COVID-19 pandemic by focusing on the ‘space’ that other researchers – to the best of our knowledge – have not yet explored in detail. More specifically, we developed four interventions grounded in the literature and tested several moderator variables to identify circumstances in which these interventions are most likely to be effective. Moreover, in contrast to the previously conducted studies, our research tested actual self-reported behaviours aimed at reducing the spread of COVID-19 rather than the intentions to undertake these behaviours. This was important considering that research on the intention–behaviour gap indicates that intentions do not always result in behaviour (Webb & Sheeran, 2006).

We had several criteria when devising the interventions. First, previous COVID-19-related research has already tested most common behavioural science interventions (e.g., social norms; Barari *et al.*, 2020) stemming from frameworks such as MINDSPACE (Dolan *et al.*, 2012), or has comprehensively examined various models of behaviour change, such as the COM-B model of Michie *et al.* (2011; see Miller *et al.*, 2020). Therefore, rather than testing again these frameworks or models from scratch, we either built upon the most promising findings that researchers who examined them identified or upon influential theories of behaviour change that were not yet investigated concerning COVID-19 when we conducted our research (e.g., inoculation theory; McGuire & Papageorgis, 1961; Van der Linden *et al.*, 2017). Second, given that most previously examined interventions involved reading simple texts and messages, we aimed to create more ‘immersive’ interventions that required participants to engage with the content. Finally, because we expected effect sizes to be small (Fetzer *et al.*, 2020), we decided to devise four interventions, which was the optimal number that allowed us to detect small effects with the sample size we were able recruit (see the ‘Determining sample size’ section below).

In one intervention we created, participants reflected on an activity they found meaningful and formulated a clear plan (i.e., implementation intentions; Oettingen *et al.*, 2015) on how they would start doing the activity the next day and overcome any potential obstacles in this regard. Importantly, participants were instructed that this activity should be something that would help them to enjoy staying at home and make it less tempting for them to go out unless necessary. Given that motivation has been found to have a strong relationship with desired behaviours during COVID-19 (Miller *et al.*, 2020), meaningfulness combined with implementation intentions and mental contrasting may activate motivational states encouraging people to stay at home and pursue the planned activity instead of going out (Oettingen *et al.*, 2015; Lunn *et al.*, 2020).

Another intervention was a message describing how adhering to strict social distancing is important for saving the economy. There are several reasons why the economic message may have a positive impact on protective behaviours during COVID-19. Because people who report higher self-interest engage less in social distancing (Oosterhoff & Palmer, 2020), saving the economy, which serves one's self-interest, may appeal to these individuals because of it matching their values and thus ‘paradoxically’ prompt them to engage in distancing behaviours (Blagov, 2020; Van Bavel *et al.*, 2020). Simultaneously, statements revolving around an outcome that is ‘best for all’ (i.e., saving the economy) should encourage feelings of collaboration, which might equally prompt individuals to comply with the desired behaviours (Lunn *et al.*, 2020).

Moreover, an ‘information intervention’ we created leveraged insights from inoculation theory that postulates the possibility of administering a ‘psychological vaccine’ against common misconceptions. Just like one would expose people to a weakened virus in the case of a biological vaccine, individuals are exposed to a weakened version of a misconception that is subsequently refuted with an evidence-based counterargument (McGuire & Papageorgis, 1961; Eagly & Chaiken, 1993; Van der Linden *et al.*, 2017; Roozenbeek & Van der Linden, 2019). Because participants are cognitively engaged in the process of assessing the misconceptions, resistance can be conferred against future exposure to similar misconceptions. An additional reason why this intervention may be effective is that it might help mitigate the ‘optimism bias’ that people have been found to carry regarding the risk of contracting COVID-19 themselves and infecting others (Kuper-Smith *et al.*, 2020; Raude *et al.*, 2020).

A fourth intervention had respondents write a letter to a loved one who is vulnerable to COVID-19. Previous studies that tested interventions grounded in empathy and concern towards others produced mixed results. Whereas Everett *et al.* (2020) and Pfattheicher (2020) showed that these interventions positively impact people's intentions to comply with the desired behaviours, in Barari *et al.* (2020), asking people to write the name of a loved one they wanted to protect had no effect. We therefore wanted to explore whether a more ‘immersive’ version of such interventions, where people would not only think about a loved one vulnerable to COVID-19 but also write a letter to that person explaining that they will do everything that is necessary to stop the spread of the virus, thus also committing themselves to this endeavour (Dolan *et al.*, 2012), would create a behavioural change. Importantly, we also wanted to understand whether the effects of interventions based on empathy and concern for others are restricted to specific subsets of the population, as examined via the moderators we describe next.

A crucial part of our research was to test whether the effects of the four interventions we developed are bounded by several moderator variables. Noting Barari *et al.*'s (2020) and Fetzer *et al.*'s (2020) reported ‘ceiling’ effects, we were aware that the behavioural interventions were unlikely to influence people already complying with the governmental policies and guidelines. However, several studies conducted on US and UK participants indicated that not all subsets of the population are complying equally, and that there is a space for improvement (e.g., Atchison *et al.*, 2020; COVID-19 Psychological Research Consortium, 2020; Oosterhoff & Palmer, 2020; Wise *et al.*, 2020). We therefore decided to conduct our study on a representative sample of US and UK participants to uncover variables that explain when interventions may be effective in influencing behaviours to reduce the spread of COVID-19.

The variables on which we focused were ‘living situation’ (i.e., to what degree people's living situation allowed them to sufficiently self-distance if necessary) and ‘economic reasons’ (i.e., whether people found it difficult to practise self-distancing for economic reasons). We expected that the interventions we developed would work specifically for people who find it difficult to self-distance for economic reasons or due to their living situation, given that such individuals may be less likely to practise social distancing and their compliance rates may not be close to the ceiling (see Ansell, 2020; Chiou & Tucker, 2020; Im *et al.*, 2020; Wright *et al.*, 2020). Therefore, these individuals could further improve, and statistically speaking there would be more space for an intervention to increase compliance. On the other hand, for the remaining individuals who are highly likely to practice social distancing given their living situation or economic reasons, compliance rates may be so high that it would be improbable for an intervention to further increase compliance from a statistical perspective due to potential ceiling effects (Barari *et al.*, 2020; Fetzer *et al.*, 2020).

Moreover, recognizing the reigning debate around ‘behavioural fatigue’ in the media and among scientists themselves (Hahn *et al.*, 2020b; Yates, 2020), we also focused on the length of time people had already spent in quarantine. Arguments around behavioural fatigue would suggest that people become tired after being in quarantine for a long time (Collinson *et al.*, 2015; Yates, 2020). One might therefore predict that those who have been self-isolating for longer may require interventions to continue to comply. However, another possibility is that people who have been self-isolating for longer could possibly have done so because of taking the disease more seriously than others and may thus continue being highly compliant. This assumption is also in line with previous research showing that past behaviour is one of the strongest predictors of future behaviour (Ajzen, 2002). Therefore, it is also possible that our interventions will not be effective for individuals who have already spent a long time in isolation. Instead, in this line of reasoning, interventions should be effective for those who started distancing more recently, given that their behaviour may indicate that they are not taking the governmental recommendations seriously and there is further space for improvement.

In short, the present study aimed to investigate how a series of newly designed behavioural interventions impact self-reported protective behaviours against COVID-19. The moderating effects of participants’ living situations, economic reasons and the lengths of time they had already spent social distancing were tested.

## Method

### Behavioural interventions

Four behavioural interventions were used in the present research. In the *letter* condition, participants were asked to think about a person vulnerable to COVID-19 they know and who means a lot to them and to write a letter to that person explaining that they will do everything that is necessary to stop the spread of the virus and to ensure this person survives the crisis.

In the *meaningful activity* condition, participants were asked to take a moment to reflect on an activity they find meaningful and that they could realistically do under the current circumstances. Then they formulated a clear plan regarding how they would start doing this activity tomorrow: they were instructed to consider the necessary steps to ensure that they are ready to start pursuing the activity and to think about any obstacles that could stop them from doing this activity and how to overcome them, etc. (Oettingen *et al.*, 2015).

In the *economy* condition, participants were asked to read a text providing an economic argument regarding why adhering to strict social distancing measures is important and can save the economy in the long run.

Finally, in the *information* condition, participants were presented with six hypothetical scenarios inspired by examples of real-life situations in which people may violate behavioural recommendations aimed at tackling the spread of COVID-19 due to various misconceptions (e.g., socializing with neighbours who live in the same building and have been compliant with staying at home). Then, after reading each scenario, they were asked a question regarding the appropriateness of the actions described in the scenario (e.g., whether it would be appropriate to visit the neighbours or invite their kids over during lockdown). Upon answering, participants were immediately provided with feedback clarifying why their answer was accurate or correcting their wrong answer with a detailed explanation that debunked the related misconceptions.

In the *control* condition, participants did not receive any experimental manipulation. Exact procedures used for each of the four interventions are available in Supplementary Materials (pp. 3–14).

### Determining sample size

Considering that we aimed to obtain a nationally representative sample consisting of UK and US participants stratified according to age, gender and ethnicity via Prolific.co, we decided to recruit the maximum number of participants this platform allows: 3000 (1500 for the USA and 1500 for the UK). We estimated that potentially 20% of these participants would not enter the final analyses either because some of them would not complete both parts of the study or because they would not meet the exclusion criteria (see the ‘Exclusion criteria’ section below), thus amounting to 2400 participants in total (or 480 per condition). We then conducted sensitivity power analyses using G*Power (Faul *et al.*, 2009) to estimate the smallest effect sizes that the study was sufficiently powered to detect, using the power of 99% and significance criterion of 0.00136, which is the significance level that the most significant effect for each set of analyses (e.g., testing main and moderated effects) would need to obtain to pass the false-discovery rate (FDR) corrections for multiple tests (Benjamini & Hochberg, 1995) that we used (the ‘Testing main effects’ and ‘Testing moderated effects’ sections below describe across how many significance tests the FDR corrections were applied for each set of analyses). We performed sensitivity power analyses only for continuous dependent variables (see the ‘Dependent variables’ section below) by relying on multiple linear regression, given that power analyses for categorical dependent variables based on logistic regression required inputting various parameters that we could not determine in advance based on previous research and produced highly variable results depending on the values of these parameters.

The sensitivity power analyses showed that the smallest main effect of an intervention (versus control) condition on a continuous dependent variable that the study was sufficiently powered to detect was 0.0127664 (Cohen's *f*^2^), whereas the smallest moderated effect was 0.0127665 (Cohen's *f*^2^). These effects sizes are generally considered small (Cohen, 1988), and the study was therefore sufficiently powered to capture small effects for continuous dependent variables considering that the final number of participants included in the statistical analyses exceeded the initial estimate of 2400. Importantly, our sample size also met the ‘corridor of stability’ criterion regarding small effects (Lakens & Evers, 2014), according to which testing more than 252 participants per condition should yield effects that are close to the ‘true’ effect sizes for the population being tested (in our case, UK participants) and should thus produce findings that are informative for policymakers who aim to apply a given intervention to this population.

### Participants, design and procedure

Out of 3014 participants who were initially recruited for Part 1, 2863 participants (1442 from the UK and 1421 from the US) completed both parts of the study (males = 1401, females = 1456, other = 6, *M*_age_ = 45.744). They were recruited via Prolific.co using their representative sample (i.e., a sample that reflects the demographics of a country concerning age, gender and ethnicity) functionality. We used a between-subjects design consisting of five levels (Condition: *Control*, *Letter*, *Meaningful Activity*, *Economy* and *Information*). The study received ethical approval from the Research Ethics Committee of the university of the first author and was conducted between 8 and 17 April 2020.

In Part 1 of the study, all participants first read the consent form, and after agreeing to participate received the questions measuring moderators and covariates (see the ‘Measures’ section below). All moderators were measured before the interventions were administered rather than thereafter, given that these variables should capture individual differences that determine the effectiveness of the interventions rather than situational states impacted by the interventions (Hayes, 2018). Thereafter, participants were randomly allocated to the five study conditions. A stratified randomization procedure was implemented, which means that randomization was performed separately within the UK and US samples to achieve a comparable distribution of participants from these two countries across conditions. After being subjected to different manipulations pertaining to each condition (see the ‘Behavioural interventions’ section above and Supplementary Materials, pp. 3–14), all participants completed the items measuring eight mediator variables (see the ‘Mediators’ section below) and were then given the opportunity to write their comments regarding the study, after which Part 1 was finished.

For Part 2, participants were contacted on the second day after finishing Part 1. They again read the consent form and were then given the questions measuring 11 dependent variables relevant to COVID-19 (see the ‘Dependent variables’ section below). Finally, after being given the opportunity to write their comments regarding the study, they were debriefed and asked whether they agreed to their responses being used in our scientific analyses (see the ‘Exclusion criteria’ section below), which was required for ethical purposes.

### Measures

#### Dependent variables

We tested nine continuous and two categorical (dichotomous) dependent variables that tackled various behaviours aimed at reducing the spread of COVID-19, from social distancing to hygiene. All of the behaviours were measured regarding ‘yesterday’ (in relation to Part 2 of the study) rather than regarding a longer period to minimize the potential confounding effects of forgetting on participants’ self-reports (Murre & Dros, 2015).

The continuous dependent variables involved *general distancing* (i.e., the extent to which participants practised social distancing); *going out times* (i.e., how many times people left their house to do any activities except for essential ones, defined as buying food or obtaining medication, going to the doctor or working if considered an ‘essential worker’ according to their country's guidelines); *going out hours* (i.e., for how many hours they left their house to do any activities except for essential ones); *physical fitness times* (i.e., how many times people left their house to maintain their physical health); *physical fitness hours* (i.e., for how many hours people left their house to maintain their physical health); *keeping distance* (i.e., whether people kept the recommended distance of at least 1.5–2.0 metres or 5–7 feet between themselves and other people if they left the house); *relative hand washing* (i.e., whether people washed their hands more than they would usually wash them before the COVID-19 crisis); *disinfect* (i.e., whether people were disinfecting any packages or foods they brought into the house); and *hand washing times* (i.e., how many times approximately they washed their hands). *General distancing* was measured on a scale from 1 (Not at all) to 5 (Extremely). *Going out times* and *physical fitness times* were measured on a scale from 0 (Staying at home all the time) to 11 (More than 10 times) in increments of 1 time. *Going out hours* and *physical fitness hours* were measured on a scale from 0 (Staying at home all the time) to 11 (More than 10 hours) in increments of 1 hour. *Keeping distance*, *disinfect* and *relative hand washing* were measured on a scale from 1 (Strongly disagree) to 7 (Strongly agree): for the first two variables, a response option ‘Does not apply to me’ was also allowed, and participants who selected it were not included in statistical analyses involving these variables. Finally, *hand washing times* was measured on a scale from 0 (Never) to 21 (More than 20 times) in increments of 1 time.

The categorical dependent variables involved *out family friends* (i.e., whether people left their house to meet their family members or friends) and *social gatherings* (i.e., whether people allowed their family members, friends or other people who do not live with them to visit them). Both variables were measured on a dichotomous response scale 0 (No) and 1 (Yes).

The exact questions and response options for all dependent variables are available in the Supplementary Materials (pp. 15–18).

#### Moderators

Three moderator variables were assessed. *Distancing history* (i.e., how many days ago people first started practising social distancing) was measured on a scale from 0 (I do not practise social distancing) to 101 (More than 100 days ago) in increments of 1 day. *Living situation* (i.e., whether people's living situation allows them to sufficiently self-distance if necessary) and *economic reasons* (i.e., the extent to which people cannot afford to practise self-distancing for economic reasons) were both assessed on a scale from 1 (Strongly disagree) to 7 (Strongly agree). The exact questions and response options for all moderator variables can be found in the Supplementary Materials (pp. 19–20).

#### Mediators

To get an insight into the mechanism behind any potential main and moderated effects of our interventions, we measured several different mediators that are conceptually linked to the interventions and/or have been identified as potential drivers of COVID-19-related behaviours in previous research (e.g., Li *et al.*, 2020; Oosterhoff & Palmer, 2020; Pfattheicher *et al.*, 2020). These mediators are *serious disease* (i.e., to what extent participants think that COVID-19 poses a serious risk for all humans), *health concern* (i.e., to what extent they think that they could be severely affected if they were to catch COVID-19), *concern close others* (i.e., concern for their close ones who are vulnerable and could get COVID-19), *concern vulnerable others* (i.e., concern for anyone who is vulnerable and could get COVID-19), *economic concern* (i.e., being concerned about how COVID-19 may impact the economy), *meaningful time* (i.e., to what extent people feel that the time they will spend at home throughout this period will be meaningful), *knowledge* (i.e., how they would rate their current knowledge regarding COVID-19) and *future intentions* (i.e., to what extent they are intending to undertake behaviours that could reduce the spread of COVID-19 going forward). *Serious disease*, *health concern*, *concern close ones*, *concern vulnerable ones*, *economic reasons*, *meaningful time* and *future intentions* were assessed on a scale from 1 (Strongly disagree) to 7 (Strongly agree), whereas *knowledge* was assessed on a scale from 1 (Not knowledgeable at all) to 5 (Extremely knowledgeable).

#### Covariates

The following covariates were measured in the present study: *household income* (i.e., participants’ feelings about their own household income these days); *education* (i.e., highest education level); *prior home* (i.e., how many days per week participants typically spent at home prior to the COVID-19 crisis); *household* (i.e., how many people, in addition to the participant, currently live in their household), *property* (i.e., the size of the property in which they live), *garden* (i.e., whether participants have access to an outdoor space they can use without being in danger of encountering other people), *key worker* (i.e., whether the participant can be considered a key worker as defined by their home country), *gender* (i.e., male versus female versus other); *country* (i.e., whether participants were from the UK or US sample); and *on time* (i.e., whether participants completed the survey for Part 2 on time – on the second day after they completed Part 1). The exact questions and response options for the covariates are presented in the Supplementary Materials (pp. 24–26), given the space restriction.

#### Exclusion criteria

To determine which participants should be excluded from the statistical analyses, we used two *instructed-response items* (Meade & Craig, 2012; Thomas & Clifford, 2017; Kung *et al.*, 2018), one measured in Part 1 and one in Part 2, and two *seriousness checks* (Aust *et al.*, 2013), one per each part. In addition, at the end of the study (Part 2), participants were asked regarding their *agreement* for their data to be used in our scientific analyses. Only participants who successfully completed both the instructed response items and seriousness checks and gave consent to use their data were included in the analyses. The exact questions and response options for all exclusion criteria items are available in the Supplementary Materials (pp. 27–28).

## Results

### Preliminary analyses

#### Excluded data

Out of 2863 participants who completed both parts of the study, 2637 were included in the statistical analyses (control condition: 550; letter condition: 478; meaningful activity condition: 525; economy condition: 539; information condition: 545) after the exclusion criteria were applied.

#### General compliance with behaviours to reduce the spread of COVID-19

To understand the general level of compliance with social distancing and other behaviours aimed at reducing the spread of COVID-19, we computed the percentage of participants who selected a particular response option for each of the behavioural dependent variables (Table 1). As can be seen from Table 1, most participants were highly compliant with social distancing. Roughly 76% of them responded with 5 for *general social distancing*, which indicates extreme compliance. Moreover, between 62% and 71% of participants responded with 0 for *going out times*, *going out hours*, *physical fitness times* and *physical fitness hours*, which means that they generally stayed at home. In addition, most participants did not leave their house to meet family members or friends (*out family friends*; 96%) and did not allow others to visit them (*social gatherings*; 97%). Finally, responses for *distancing*, *relative hand washing*, *hand washing times* and *disinfect* indicate that people largely tried to keep a distance of 1.5–2.0 metres between themselves and others when outside and to maintain appropriate hygiene.

**Table 1.** The percentage of participants who selected a response option for each of the 11 behavioural dependent variables.

Response option	A	B	C	D	E	F	G	H	I	J	K
0		70.99	71.44	62.76	62.42	96.40	96.89			0.11	
1	0.38	23.28	20.93	31.74	30.91	3.60	3.11	0.63	1.37	0.57	9.44
2	0.83	4.21	5.38	4.10	5.54			0.95	4.25	2.05	18.37
3	2.96	0.72	0.99	0.64	0.72			0.95	3.53	4.13	7.41
4	20.17	0.19	0.38	0.49	0.27			1.52	6.79	7.62	7.80
5	75.65	0.34	0.23	0.15	0.04			5.71	14.33	10.54	12.83
6		0.08	0.08	–	0.08			23.10	32.92	12.67	18.49
7		–	–	0.04	–			67.13	36.82	6.83	25.66
8		0.08	0.23	–	–					9.94	
9		–	0.15	0.04	–					3.00	
10		0.04	–	0.04	0.04					19.04	
11		0.08	0.19	–	–					0.83	
12										6.03	
13										0.61	
14										1.33	
15										5.95	
16										0.68	
17										0.34	
18										0.53	
19										0.11	
20										2.39	
21										4.70	
Total *n*	2637	2637	2637	2637	2637	2637	2636	1576	2637	2637	1769

*Notes:* ‘Response option’ refers to a response option that participants could select for a dependent variable (see the ‘Dependent variables’ section, as well as the Supplementary Materials, pp. 15–18, for specifics regarding the response options).

Columns A–K correspond to each of the 11 dependent variables as follows: A = general distancing; B = going out times; C = going out hours; D = physical fitness times; E = physical fitness hours; F = out family friends; G = social gatherings; H = keeping distance; I = relative hand washing; J = hand washing times; K = disinfect.

*Numbers for each variable* indicate the percentage (%) of participants who answered with the corresponding response option.

*Light grey shading* indicates the range of possible response options for each variable, and *dark grey cells* indicate the highest desirable response option (e.g., for *general distancing*, response option 5 indicates the highest possible compliance with social distancing).

‘Total *n*’ at the bottom of the table corresponds to the total number of participants who responded for each variable.

### Main analyses

We tested both main and moderated effects of the interventions on the dependent variables. Our general analytic approach was to first test all of the main effects of the interventions (versus control) and the moderated effects for each moderator. Then, we applied the FDR correction of Benjamini and Hochberg (1995) to probe whether the significant effects we identified remained significant despite multiple comparisons. Finally, for all of the effects that remained significant after the FDR correction, we tested whether they would remain significant when controlling for covariates. Therefore, for an effect to be identified as robust, it had to both pass the FDR correction (Benjamini & Hochberg, 1995) and remain significant despite covariates. For all of the effects we identified as robust, we then conducted mediation analyses (for main effects) or moderated mediation analyses (for moderated effects) to identify whether the mediators we measured could provide further insights into the mechanism behind the effects. Although based on previous research we expected that the distribution of our continuous dependent variables may be skewed (Barari *et al.*, 2020), we used linear regression for all analyses involving these variables, given that for sample sizes larger than 500 this technique is highly robust regarding most extreme violations of normality and is preferred over non-parametric tests (Lumley *et al.*, 2002). Descriptive statistics and zero-order correlations between all continuous variables tested in the study can be seen in the Supplementary Materials.

### Testing main effects

For each of the nine continuous dependent variables, we performed a multiple linear regression analysis, and for each of the two dichotomous dependent variables, we performed a logistic regression analysis. In each regression analysis, four dummy variables were included as predictors: one for the letter condition, one for the meaningful activity condition, one for the economy condition and one for the information condition. Therefore, 44 effects in total were tested across 11 regression analyses (11 analyses × 4 effects corresponding to each of the 4 conditions per analysis). Only two effects were significant. More specifically, the letter condition decreased the number of hours participants spent outside compared to the control condition, *b* = –0.126, 95% confidence interval (CI) = –0.247 to –0.005, *t*(2632) = –2.041, p = 0.041, whereas the information condition made participants less likely to allow their family members, friends or other people to visit them compared to the control condition, odds ratio = 0.423, 95% CI = 0.192–0.932, Wald = 4.556, p = 0.033. However, after the FDR correction (Benjamini & Hochberg, 1995) that considered all 44 of the significance tests concerning the main effects was implemented, no effects remained significant. Therefore, we concluded that no interventions had a robust influence on one or more dependent variables. We thus present all 11 regression analyses that probed the main effects in the Supplementary Materials (pp. 32–36) due to space restrictions.

### Testing moderated effects

#### Distancing history

To test the effect of the intervention conditions (versus control) on the dependent variables as moderated by *distancing history*, we performed 11 multiple regression analyses – nine linear regressions for the continuous dependent variables and two logistic regressions for the categorical dependent variables. In each regression analysis, four interaction effects (one between each condition and distancing history) were computed, thus amounting to 44 interaction effects in total across the 11 regression analyses. Seven significant interaction effects were identified (see the Supplementary Materials, pp. 37–51). However, after the FDR correction (Benjamini & Hochberg, 1995) that considered all 44 of the significance tests concerning the interaction effects was implemented, three of these effects remained significant. These were the interactions between the information condition and distancing history for *general distancing*, *going out times* and *going out hours* as dependent variables. Linear regression analyses involving these interaction effects are presented in Tables 2, 3 and 4, respectively.

**Table 2.** Multiple linear regression for the influence of the interactions between the intervention conditions and distancing history on general distancing.

	95% CI for *b*	
Condition	*b*	SE	*β*	*t*	p	Lower	Upper
Constant	4.367	0.065		66.977	<0.001	4.239	4.495
Letter	0.078	0.097	0.050	0.801	0.423	–0.113	0.269
Meaningful activity	0.113	0.089	0.075	1.276	0.202	–0.061	0.287
Economy	0.155	0.093	0.103	1.675	0.094	–0.026	0.336
Information	0.259	0.089	0.174	2.925	0.003	0.086	0.433
Distancing history	0.016	0.003	0.251	5.584	<0.001	0.010	0.022
Int. 1	–0.005	0.004	–0.074	–1.168	0.243	–0.013	0.003
Int. 2	–0.006	0.004	–0.092	–1.512	0.131	–0.013	0.002
Int. 3	–0.008	0.004	–0.125	–1.967	0.049*^a^*	–0.016	0.000
Int. 4	–0.012	0.004	–0.193	–3.056	0.002	–0.019	–0.004

*Notes:* Model *R*^2^ = 0.027.

Control condition is the reference category.

*^a^*Initially significant interaction effects that stopped being significant after the false-discovery rate correction was applied.

CI = confidence interval; Int. 1 = interaction between *letter* and *distancing history*; Int. 2 = interaction between *meaningful activity* and *distancing history*; Int. 3 = interaction between *economy* and *distancing history*; Int. 4 = interaction between *information* and *distancing history*.

**Table 3.** Multiple linear regression for the influence of the interactions between the intervention conditions and distancing history on going out times.

	95% CI for *b*	
Condition	*b*	SE	*β*	*t*	p	Lower	Upper
Constant	0.793	0.087		9.151	<0.001	0.623	0.963
Letter	–0.243	0.129	–0.117	–1.881	0.060	–0.497	0.010
Meaningful activity	–0.203	0.118	–0.102	–1.725	0.085	–0.434	0.028
Economy	–0.236	0.123	–0.119	–1.917	0.055	–0.477	0.005
Information	–0.479	0.118	–0.243	–4.065	<0.001	–0.710	–0.248
Distancing history	–0.018	0.004	–0.206	–4.560	<0.001	–0.025	–0.010
Int. 1	0.008	0.006	0.094	1.482	0.139	–0.003	0.020
Int. 2	0.008	0.005	0.091	1.488	0.137	–0.002	0.018
Int. 3	0.008	0.005	0.100	1.564	0.118	–0.002	0.019
Int. 4	0.021	0.005	0.262	4.126	<0.001	0.011	0.031

*Notes:* Model *R*^2^ = 0.016.

Control condition is the reference category.

CI = confidence interval; Int. 1 = interaction between *letter* and *distancing history*; Int. 2 = interaction between *meaningful activity* and *distancing history*; Int. 3 = interaction between *economy* and *distancing history*; Int. 4 = interaction between *information* and *distancing history*.

**Table 4.** Multiple linear regression for the influence of the interactions between the intervention conditions and distancing history on going out hours.

	95% CI for *b*	
Condition	*b*	SE	*β*	*t*	p	Lower	Upper
Constant	0.896	0.107		8.366	<0.001	0.686	1.106
Letter	–0.447	0.160	–0.175	–2.794	0.005	–0.760	–0.133
Meaningful activity	–0.290	0.146	–0.117	–1.986	0.047	–0.575	–0.004
Economy	–0.241	0.152	–0.099	–1.584	0.113	–0.539	0.057
Information	–0.518	0.146	–0.213	–3.553	<0.001	–0.804	–0.232
Distancing history	–0.019	0.005	–0.183	–4.037	<0.001	–0.029	–0.010
Int. 1	0.016	0.007	0.141	2.204	0.028*^a^*	0.002	0.029
Int. 2	0.011	0.006	0.108	1.748	0.081	–0.001	0.024
Int. 3	0.009	0.007	0.081	1.274	0.203	–0.005	0.022
Int. 4	0.020	0.006	0.206	3.236	0.001	0.008	0.033

*Notes:* Model *R*^2^ = 0.011.

Control condition is the reference category.

*^a^*Initially significant interaction effects that stopped being significant after the false-discovery rate correction was applied.

CI = confidence interval; Int. 1 = interaction between *letter* and *distancing history*; Int. 2 = interaction between *meaningful activity* and *distancing history*; Int. 3 = interaction between *economy* and *distancing history*; Int. 4 = interaction between *information* and *distancing history*.

**Figure 1.**
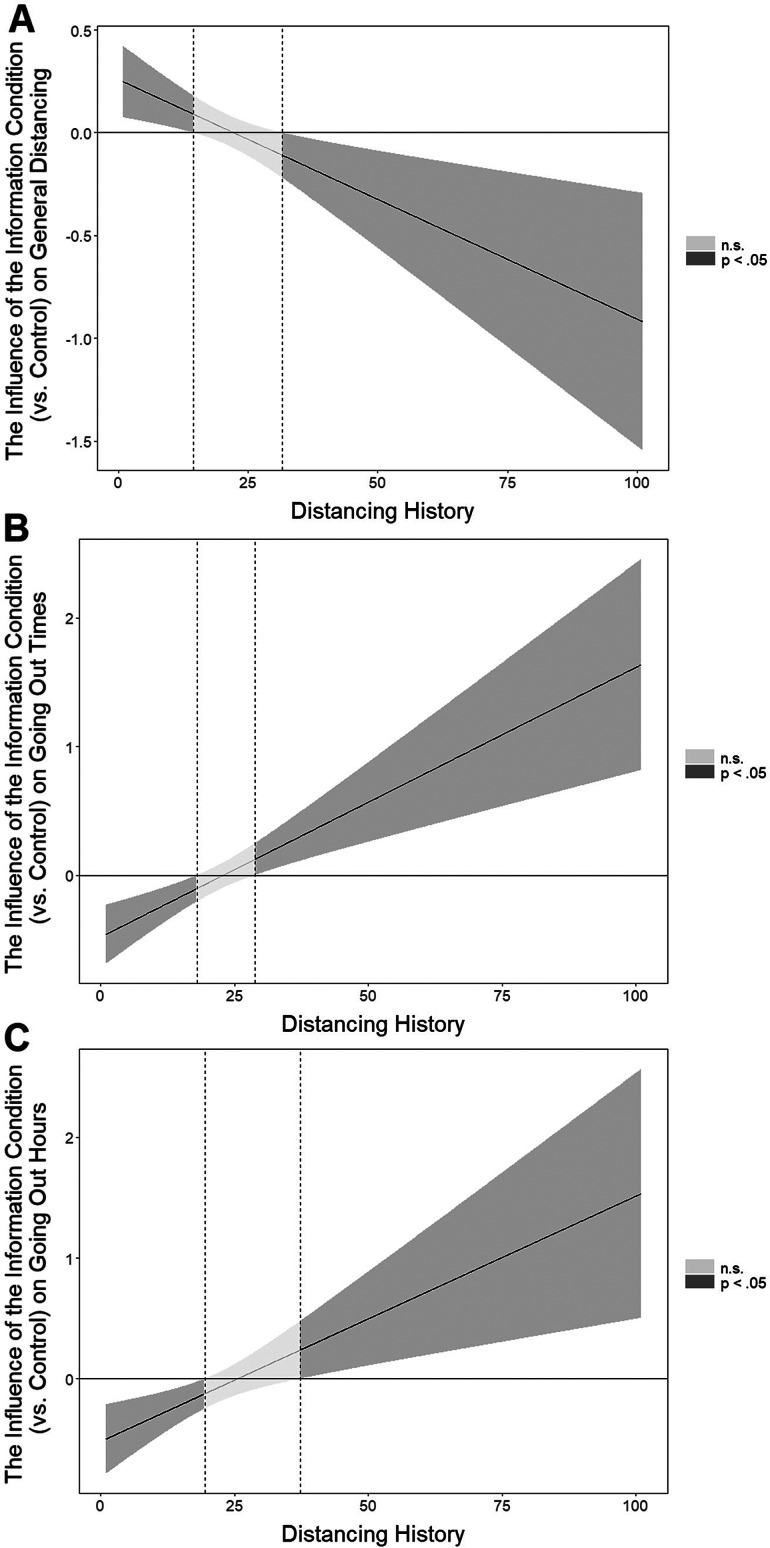
The influence of the information (versus control) condition on (A) general distancing, (B) going out times and (C) going out hours at different levels of distancing history, which corresponds to how many days before the intervention participants first started practising social distancing. The mean value of distancing history is 21.

To further probe the pattern of these three interactions, we used the Johnson–Neyman technique (Johnson & Fay, 1950; Bauer & Curran, 2005; Long, 2019). Given that this technique involves computing the effect of conditions on a dependent variable for many different levels of the moderator, a FDR correction by Esarey and Sumner (2018) was used to minimize the chance of false-positive findings. Although the Johnson–Neyman technique is the most recommended approach when probing interaction patterns (e.g., Spiller *et al.*, 2013), the exact number of days that the analysis outputs should not be taken too literally by policymakers, and they should interpret the interaction patterns more generally (e.g., by focusing on how the intervention impacted people who started practising social distancing early versus recently, in line with how we explain the findings). As can be seen from Figure 1A, the information condition (versus control) positively impacted general distancing for participants who started practising social distancing recently (14 or fewer days ago), whereas it had a negative effect for participants who started practising it relatively early (32 or more days ago). Similarly, the information condition (versus control) decreased the number of times people went out for those who started practising social distancing more recently (18 or fewer days ago), whereas it increased it for people who started distancing relatively early (29 or more days ago; Figure 1B). Finally, the information condition (versus control) decreased the number of hours people spent outside for those who started practising social distancing recently (up to 19 days ago), whereas it increased it for people who started practising social distancing earlier (37 or more days ago; Figure 1C).

To further demonstrate the robustness of the three significant interaction effects, we computed the same analyses testing these effects as described above, but this time with covariates included in the regression models (Supplementary Materials, pp. 83–90).

The interaction between the information (versus control) condition and distancing history was again significant in influencing general distancing, *b* = –0.011, 95% CI = –0.018 to –0.004, *t*(2600) = –3.050, p = 0.002. The pattern of the interaction also remained similar (Supplementary Materials, pp. 83–85). Moreover, the interaction between the information (versus control) condition and distancing history also remained significant in influencing going out times, *b* = 0.021, 95% CI = 0.011–0.031, *t*(2600) = 4.164, p < 0.001, and the pattern of the interaction remained similar (Supplementary Materials, pp. 85–87). Finally, the interaction between the information (versus control) condition and distancing history was again significant in influencing going out hours, *b* = 0.021, 95% CI = 0.009–0.033, *t*(2600) = 3.398, p = 0.001. The pattern of the interaction was also similar (Supplementary Materials, pp. 88–90). Therefore, the results with and without the covariates included in the regression models were almost identical, thus demonstrating the robustness of the findings.

Considering that all three of the interaction effects remained significant despite the FDR corrections (Benjamini & Hochberg, 1995) or covariate testing and were therefore robust, we finally conducted a moderated mediation analysis for each interaction effect. This analysis allows probing of whether some of the mediators we measured can explain the influence of the information (versus control) condition on *general distancing*, *going out times* or *going out hours* at different levels of the moderator (distancing history) for which these effects were significant. All of the moderated mediation analyses were conducted using the *Process* package (Model 8) of Hayes (2018) implemented in *SPSS*. The percentile bootstrapping procedure with 10,000 resamples was used. All eight mediators were included in a moderated mediation analysis in parallel, which means that the analysis accounted for the correlations between them when computing the mediated effects. The analyses showed that none of the moderated mediation effects were significant at p < 0.05, given that the 95% CI of the Index of Moderated Mediation for each mediator contained 0 (Hayes, 2018). Overall, given that these analyses did not detect a mechanism behind the influence of the information condition on the dependent variables at different levels of distancing history, based on the present research we cannot reliably speculate as to whether this influence would generalize to some other policy interventions that may share a similar mechanism.

#### Living situation

To test the effect of the intervention conditions (versus control) on the dependent variables as moderated by *living situation*, we computed the same analyses as for the previous moderator. More specifically, we conducted 11 multiple regression analyses – nine linear regressions for the continuous dependent variables and two logistic regressions for the categorical dependent variables. In each regression analysis, four interaction effects (one between each condition and living situation) were computed, thus resulting in 44 interaction effects in total. Nine significant interactions were identified, which generally showed that our interventions tended to improve different behaviours aimed at reducing the spread of COVID-19 only for participants whose living situation did not allow them to sufficiently self-distance from others if necessary. However, after the FDR correction (Benjamini & Hochberg, 1995) that considered all 44 of the significance tests concerning the interaction effects was implemented, none of the effects remained significant. We therefore present the analyses for all of the interactions between the intervention (versus control) conditions and living situation in the Supplementary Materials (pp. 52–68) for informative purposes.

#### Economic reasons

To test the impact of the interactions between the intervention conditions (versus control) and *economic reasons* on the dependent variables, we again conducted 11 multiple regression analyses – nine linear regressions for the continuous dependent variables and two logistic regressions for the categorical dependent variables. In each regression analysis, four interaction effects (one between each condition and economic reasons) were computed, which resulted in 44 interaction effects. Five significant interactions were identified, which generally indicated that our interventions tended to improve behaviours aimed at reducing the spread of COVID-19 only for participants who could not easily afford to practice self-distancing due to economic reasons. However, after applying the FDR correction (Benjamini & Hochberg, 1995) that considered all 44 of the significance tests concerning the interaction effects, none of the effects remained significant. We therefore present the analyses for *economic reasons* as a moderator in the Supplementary Materials (pp. 69–82) for informative purposes.

## General discussion

The present research sought to investigate the impact of four behavioural interventions (versus control) on respondents’ self-reported social distancing and protective behaviours against COVID-19 a day after the intervention. We found indications that the letter condition (versus control) decreased the number of hours participants spent outside and that the information condition (versus control) made participants less likely to allow their family members, friends or other people to visit them. However, these effects did not survive corrections for multiple comparisons. Therefore, like other previous research (e.g., Barari *et al.*, 2020; Everett *et al.*, 2020), we had to conclude that behavioural interventions did not have a direct, robust influence on behaviours that could reduce the spread of COVID-19.

Where our research adds new insights, however, is in showing that there is a subgroup of people who do seem to benefit from behavioural interventions during the COVID-19 pandemic. Specifically, for people who started practising social distancing only recently, the information (versus control) condition improved general distancing and made them go outside less, whereas it had an undesirable effect regarding these variables for those who had been practising social distancing for longer. These effects remained significant even after controlling for a wide range of demographic variables. Considering that the correlations between distancing history and various behaviours aimed at reducing the spread of COVID-19 that we measured generally indicated that people who had been practising social distancing for longer were also more likely to comply with these behaviours (Supplementary Materials, p. 31), our findings suggest that the interventions worked for less disciplined individuals for whom there was space for further improvement. Although the significant interactions between our interventions and living situation or economic reasons did not withstand corrections for multiple comparisons, the patterns of these interactions did point in the same direction that behavioural interventions work better for people who have more trouble complying with social distancing measures (Supplementary Materials, pp. 52–82).

These results have various policy implications. For one, we have confirmed what other researchers have found as well: that a blanket approach to implementing behavioural interventions in a situation where many people already comply may not be meaningful. However, our findings indicate that certain behavioural interventions may work for subgroups of individuals, specifically for those who started practising social distancing only recently – an insight that may more generally apply to individuals who have trouble complying with social distancing. The reason for why this effect occurs remains to be further explored, given that none of the eight mediating variables we tested yielded significant effects. One possible clue might lie in Wise *et al.*'s (2020) work showing that the ‘personal risk of infection’ is one of the strongest drivers of social distancing and handwashing. We did not explicitly test for this narrow mediator, but it is plausible that our information condition may have created the behavioural change by influencing people's perceived personal risk of getting infected with COVID-19. Regardless, our research suggests that targeting people who are late adopters and potentially those who have trouble self-distancing with information-driven behavioural interventions can be fruitful. Practically, this may mean that governments would want to share more information or conduct a social inoculation game with people they find to be transgressing or in areas where social distancing is known to be difficult.

Another interesting finding was that the information intervention could backfire for those who practised social distancing for longer periods. This would suggest that the ‘behavioural fatigue’ explanation does not hold, at least not to the extent that people who have been distancing for a long time already need extra encouragement to continue to adhere to the guidelines.^1^ What the ‘backfiring’ result might indicate is a psychological reactance effect (Brehm, 1966; Rains, 2013), meaning that participants who already were convinced of the need to distance themselves might have become irritated by being told to comply even more and therefore ‘lashed out’ by complying less. Alternatively, these people may have been the ones who took COVID-19 very seriously from the very start, perhaps even over-catastrophizing the situation. It is possible that the information condition corrected their understanding in making them more realistic about the situation in the opposite direction from those who started distancing only recently, thus leading them to see the disease as a bit less of a risk for themselves than they previously thought. More specifically, ‘Scenario 6’ of the information condition where more concrete numbers were given regarding the risk of contracting COVID-19 could have had this effect.

Given that the information intervention was generally most effective in the present research, it is important to additionally discuss this insight in relation to previous behavioural science literature. Typically, it has been argued that the potential for information-based interventions is limited, and that various interventions that change the context in which people act and target automatic processes (e.g., defaults, norms, etc.) are preferred (Marteau *et al.*, 2012; Sanders *et al.*, 2018). Our research, however, indicates that information-based interventions may be more promising than assumed, and that investigating techniques that could make such interventions more effective may be a fruitful area for future research. For example, given that our intervention leveraged the inoculation theory (Van der Linden *et al.*, 2017), this theory may produce valuable insights regarding how to use information in policymaking to instigate behavioural change.

Finally, in order to understand the implications of our research, it is also important to understand its limitations. First, we investigated an online sample of participants. Although we tested a representative sample in terms of age, gender and ethnicity, we cannot exclude the possibility that respondents on Prolific.co are more used to computer work at home or otherwise different from the ‘average’ UK or US citizen. Moreover, we did not have the opportunity to replicate our findings, which would in a normal research scenario be the preferred route of action (although we did conduct a highly powered study with many participants). Finally, given that we tested the intervention effects over a relatively short period of time to minimize potential confounding effects of forgetting on behavioural self-reports, we cannot ascertain whether the effects we demonstrated would also occur over a longer period.

## Conclusion

Overall, this research found that behavioural science can inform meaningful interventions in times of COVID-19, and that the specific circumstances in which people find themselves matter concerning the impact of these interventions. An information-based intervention increased compliance with social distancing and reduced the number of times and hours people went outside for non-essential errands, but only for those who had started practising distancing relatively late, whereas it backfired for those who had been practising social distancing for a long time. Future research might look to replicate this effect, as well as explore further mechanisms and moderator variables that may help inform policymakers concerning whom to target with behavioural interventions in order to reduce the spread of COVID-19.

## Footnotes

1 Because based on previous research it is not known how much time it takes for behavioural fatigue effects to occur, we acknowledge that it is possible that the fatigue had not yet set in at the time when we conducted the study. Therefore, our conclusions about behavioural fatigue in the context of the present research should be taken with caution.

## Supplementary material

For supplementary material accompanying this paper visit http://dx.doi.org/10.1017/bpp.2020.48. click here to view supplementary material
