# Supplementary material for: When behavioural science can make a difference in times of COVID-19
Source: Behav Public Policy. 2020 Sep 1:1–27. doi: 10.1017/bpp.2020.48 (PMC7503042; doi:10.1017/bpp.2020.48)
Supplement: Supplementary file 1 [file S2398063X20000482sup001.docx]

**Supplementary Materials: Table of Contents**

[**List and Description of Variables from the Present Research** 3](#_Toc48374580)

[**INDEPENDENT VARIABLE** 3](#_Toc48374581)

[**DEPENDENT VARIABLES** (all dependent variables were measured in Part 2 of the study) 15](#_Toc48374582)

[**MODERATORS** (all moderators were measured in Part 1 of the study) 19](#_Toc48374583)

[**MEDIATORS** (all mediators were measured in Part 1 of the study) 21](#_Toc48374584)

[**COVARIATES** (all covariates were measured in Part 1 of the study) 24](#_Toc48374585)

[**EXCLUSION CRITERIA ITEMS** 27](#_Toc48374586)

[**Descriptive Statistics** 29](#_Toc48374587)

[**Zero-order Correlations Between All Continuous and Dichotomous Categorical Variables** 31](#_Toc48374588)

[**Main Effects: The Influence of the Intervention Conditions on the Dependent Variables** 32](#_Toc48374589)

[**Moderated Effects: The Influence of the Intervention Conditions on the Dependent Variables as Moderated by Distancing History** 37](#_Toc48374590)

[**Moderated Effects: The Influence of the Intervention Conditions on the Dependent Variables as Moderated by Living Situation** 52](#_Toc48374591)

[**Moderated Effects: The Influence of the Intervention Conditions on the Dependent Variables as Moderated by Economic Reasons** 69](#_Toc48374592)

[**Covariate Testing for the Interaction Effects That Remained Significant After the False Discovery Rate (FDR) Correction Was Applied** 83](#_Toc48374593)

[**References** 91](#_Toc48374594)

# **List and Description of Variables from the Present Research**

## **INDEPENDENT VARIABLE**

Intervention (experimentally manipulated in Part 1 of the Study)*^[[1]](#footnote-1)^

This is a between-subjects variable consisting of five conditions: Control (0); Letter – Intervention 1 (1), Meaningful Activity – Intervention 2 (2), Economy – Intervention 3 (3); Information – Intervention 4 (4)

- In the Control Condition, participants received no experimental manipulation.
- In the Letter Condition, all participants received the following experimental manipulation:

Think about **a person vulnerable to COVID-19 you know and who means a lot to you** (e.g. your grandparent, parent, partner, friend, relative, neighbour, etc.). 
Please write down who that person is - you don't need to write their name, you can just state their relationship to you (e.g. grandparent, parent, partner, friend, etc.).

________________________________________________________________

Now, please write a letter to that person explaining that you will do **anything that is necessary to stop the spread of COVID-19 and ensure they survive the crisis**.
You can describe the specific actions you will undertake (including the recommended actions which include staying at home unless you absolutely need to go out for essential reasons, washing your hands, keeping a distance of at least 1.5-2 meters or 5-7 feet from others when you need to leave the house, etc.). Also, you can explain why this person means a lot to you and why it is important to you that they stay alive.

________________________________________________________________

________________________________________________________________

________________________________________________________________

________________________________________________________________

________________________________________________________________

- In the Meaningful activity Condition, all participants received the following experimental manipulation:

This time of COVID-19 pandemic is a good time to focus on personal growth and development. It gives time to do something you have always wanted to do that you find meaningful. 
**Take a moment to reflect and think about something you find meaningful** and that you could realistically do under the current circumstances, while COVID-19 pandemic is ongoing (it would need to be something that you can do at home). This should be a meaningful activity that would help you to enjoy staying at home and make it less tempting for you to go out unless absolutely necessary.  
Please state what this activity is and briefly describe it.

________________________________________________________________

________________________________________________________________

________________________________________________________________

________________________________________________________________

________________________________________________________________

Now, please **formulate a clear plan of how you will start doing this activity tomorrow**. Please describe the following:

- - Which exact steps you need to take to make sure you are ready to start doing this activity tomorrow.
  - When exactly tomorrow you will do this activity and for how long.
  - ​​​How you will overcome any obstacles that may prevent you from doing this activity.
  - ​​​​How this activity will make it easier for you to stay home and practise social distancing.

________________________________________________________________

________________________________________________________________

________________________________________________________________

________________________________________________________________

________________________________________________________________

- In the Economy Condition, all participants received the following experimental manipulation:

**The Hammer and the Dance**

A strategy that can most effectively mitigate negative economic consequences of the COVID-19 crisis is called “The Hammer and the Dance”.

In the “hammer” stage, it is important to act quickly and aggressively. People need to comply with strict social distancing measures. They need to stay at home unless it is absolutely necessary that they leave the house (e.g. to buy food). When they do need to go out, they need to keep the distance of at least 1.5-2 meters or 5-7 feet between themselves and other people. They need to wash their hands frequently, 20 seconds at a time. They need to disinfect any packages or food items that they bring into their homes. This hammer stage would last several weeks.

After COVID-19 has been “hammered”, in the “dance” stage the virus would be kept under control until there’s a vaccine.

If people comply with the strict social distancing measures over several weeks and “hammer” the virus, our economy can survive this brief “shock” and again prosper in the long run.

**If people don’t comply with the strict social distancing measures, the period of isolation and distancing will need to be prolonged to stop the virus. This will destroy the economy and may lead to an unprecedented economic crisis.**

If you want to save the economy in the long run and make sure people keep their jobs, take social distancing measures seriously NOW!

If you have read this text, please press "Yes" and go to the next page.

- No, I have not read the text
- Yes, I have read the text
- In the Information Condition, all participants received the following experimental manipulation:

You will be shown a number of scenarios and you will be asked a question related to each. You will receive immediate results after answering each question.

**Scenario 1**

You have to go out to run an important errand and buy groceries for your home. While out you should maintain “social distancing” with the elderly and vulnerable people.
Would this approach reduce the risk of contracting the virus?

- No
- Yes

Display This Question:

If You have to go out to run an important errand and buy groceries for your home. While out you shou... = No

Correct, “social distancing” should be exercised with everyone.


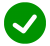


Display This Question:

If You have to go out to run an important errand and buy groceries for your home. While out you shou... = Yes

Incorrect, while the elderly have statistically higher mortality rates of the virus, younger people are equally likely to contract the virus. Additionally, “social distancing” should be exercised with everyone since the chance of contracting the virus is equally spread across all nationalities and ethnicities.


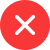


**Scenario 2**

Your next door neighbours who you have known and been good friends with for years invite you for a coffee to catch up. You have been going for a walk with them on a daily basis or your kids have been having playing dates almost every day. You also know that your neighbours have been compliant with staying at home. In the end, visiting them would not entail leaving the building since their door is a few meters away from yours. 
When social distancing is called for by the government going forward, would you still go for a walk with them and/ or invite their kids over?

- No
- Yes

Display This Question:

If Your next door neighbours who you have known and been good friends with for years invite you for... = No

Correct! Even though your neighbours appear to be staying at home, they might have contracted the virus via another visitor to their place, or by going to the grocery store.


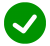


Display This Question:

If Your next door neighbours who you have known and been good friends with for years invite you for... = Yes

Incorrect, this still imposes a risk for contracting the virus. Even if they might have not left their home, they might have been exposed to others (e.g. visitors, delivery individuals, etc.)


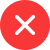


**Scenario 3**

It is the family tradition to gather on the weekend for lunch with family and/ or friends. You plan a family or friend dinner and you want to be socially responsible by checking with family/ friends members^[[2]](#footnote-2)^ whom you plan to invite to see whether they have any symptoms of COVID-19 before telling them about your dinner plan. 
Does inviting them to dinner impose risk of contracting the virus?

- No
- Yes

Display This Question:

If It is the family tradition to gather on the weekend for lunch with family and/ or friends. You pl... = No

Incorrect, even though they are ‘family’ and have no reported symptoms so far, you cannot guarantee that for those who do not reside in the same household as you are not carrying the virus. Symptoms might take upwards of two weeks to surface.


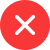


Display This Question:

If It is the family tradition to gather on the weekend for lunch with family and/ or friends. You pl... = Yes

Correct! COVID-19 symptoms might not show for a period reaching up to two weeks and therefore one cannot ensure that the person does not carry the virus.


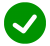


**Scenario 4**

You are a regular visitor to a local facility (e.g. gym) and you have been constantly using this facility for a long time and made several visits since the first Corona case was announced in your country. However, you are still completely healthy and you notice that the management of the facility is stepping up the efforts to clean and disinfect surfaces.
When social distancing is called for by the government going forward, would you still visit facilities that have stepped up their efforts to clean and disinfect their buildings. Do you agree?^[[3]](#footnote-3)^

- No
- Yes

Display This Question:

If You are a regular visitor to a local facility (e.g. gym) and you have been constantly using this... = No

Q52 Correct! stepping up cleaning and disinfection in a facility does not rule out coming into direct contact with individuals who might have contracted the virus. You might have been going to the gym in the last couple of weeks, and still feel healthy today. The spread of the virus is increasing on a daily basis and the situation is not comparable to previous days. Precautions should be always taken by staying at home and avoiding going to the gym or crowded areas.


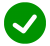


Display This Question:

If You are a regular visitor to a local facility (e.g. gym) and you have been constantly using this... = Yes

Q53 Incorrect, stepping up cleaning and disinfection in a facility does not rule out coming into direct contact with individuals who might have contracted the virus. You might have been going to the gym in the last couple of weeks, and still feel healthy today. The spread of the virus is increasing on a daily basis and the situation is not comparable to previous days. Precautions should be always taken by staying at home and avoiding going to the gym.


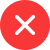


**Scenario 5**

If you are a healthy person who takes all important precautions and abides by the guidelines issued by the Ministry of Health and the World Health Organization, then you impose no risk to yourself and your family.
Do you agree with this statement?

- No
- Yes

Display This Question:

If If you are a healthy person who takes all important precautions and abides by the guidelines issu... = No

Correct! One is always at risk of contracting the virus and transmitting it to those around even if no symptoms are reported.


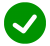


Display This Question:

If If you are a healthy person who takes all important precautions and abides by the guidelines issu... = Yes

Incorrect. While the preventative measures do reduce the likelihood of contracting the virus, you are still at risk of becoming a carrier of the virus and infecting those around you even if you might not exhibit serious symptoms.


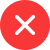


**Scenario 6**

Out of the total number of Corona cases worldwide, roughly 1 in every 4 people who have contracted the virus have recovered while many more are on their way to recovery. In fact, 96% of currently active cases are not considered to be serious or critical. This means that “it is just a flu” and contracting it only improves your immunity.
Do you agree with this statement?

- No
- Yes

Display This Question:

If Out of the total number of Corona cases worldwide, roughly 1 in every 4 people who have contracte... = No

Correct. First, the death rate is a moving average and researchers still do not have a full understanding of the COVID19 and its implications. Second, each family has vulnerable and elderly members who are at high risk. Even if the rate of recoveries is high and a large majority of cases are considered mild. However, it is still important to take precautions even if you are not part of the ‘vulnerable’ group as you can still suffer from days or weeks of illness and could also infect those who are vulnerable.


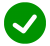


Display This Question:

If Out of the total number of Corona cases worldwide, roughly 1 in every 4 people who have contracte... = Yes

Incorrect! First, the death rate is a moving average and researchers still do not have a full understanding of the COVID19 and its implications. Second, each family has vulnerable and elderly members who are at high risk. Even if the rate of recoveries is high and a large majority of cases are considered mild. However, it is still important to take precautions even if you are not part of the ‘vulnerable’ group as you can still suffer from days or weeks of illness and could also infect those who are vulnerable.


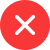


## **DEPENDENT VARIABLES** (all dependent variables were measured in Part 2 of the study)

- General Distancing

To what extent did you practise social distancing yesterday? Social distancing can involve any of the following:

- - working from home, where possible (e.g., if you are not considered an "essential worker" in your country);
  - avoiding large gatherings and gatherings in smaller public spaces such as cinemas, restaurants, etc.;
  - avoiding non-essential use of public transport;
  - avoiding gatherings with friends and family, unless you live together with them;
  - self-isolating because you are experiencing symptoms of COVID-19 or because you are among the vulnerable population;
  - leaving your house only for essential activities (shopping for groceries or medications; or work if you are considered an "essential worker" in your country).

Response options were: Not at all (1); A little (2); Moderately (3); Quite a bit (4); Extremely (5)

- Going Out Times

Yesterday, how many times did you leave your house to do any activities EXCEPT FOR the following ones: buying food, buying medication, going to the doctor, or working (if you are considered an "essential worker" in your country)?

Response options were: 0 (I stayed at home all the time except when I had to buy food or medication, go to the doctor, or go to work if I am considered an "essential worker") (0); 1 - Once(1); … ; 11 - More than ten times (11)

- Going Out Hours

Yesterday, for how many hours did you leave your house to do any activities EXCEPT FOR the following ones: buying food, buying medication, going to the doctor, or working (if you are considered an "essential worker" in your country)?

Response options were: 0 (I stayed at home all the time except when I had to buy food or medication, go to the doctor, or go to work if I am considered an "essential worker") (0); 1 - Up to one hour (1); … ; 11 - More than ten hours (11)

- Physical Fitness Times

Yesterday, how many times did you leave your house to maintain your physical health (e.g. going for a walk, exercising, etc.)?

Response options were: 0 (I stayed at home all the time except when I had to go out to do essential activities) (0); 1 - Once (1); … ; 11 - More than ten times (11)

- Physical Fitness Hours

Yesterday, for how many hours did you leave your house to maintain your physical health (e.g. going for a walk, exercising, etc.)?

Response options were: 0 (I stayed at home all the time except when I had to go out to do essential activities) (0); 1 - Up to one hour (1); … ; 11 - More than ten hours (11)

- Out Family Friends*

Yesterday, did you leave your house to meet your family members or friends?

Response options were: No (0); Yes (1)

- Social Gatherings*

Yesterday, did you allow your family members, friends, or other people (who do not live with you) to visit you?

No (0); Yes (1)

- Keeping Distance

To what extent do you agree or disagree with the following statement: "Yesterday, whenever I left the house, I was making sure to keep the recommended distance (e.g. at least 1.5-2 meters or 5-7 feet) between myself and other people."

Response options were: Does not apply to me (I did not leave my house yesterday) (8); Strongly disagree (1); Disagree (2); Somewhat disagree (3); Neither agree nor disagree (4); Somewhat agree (5); Agree (6); Strongly agree (7)

- Relative Hand Washing

To what extent do you agree or disagree with the following statement: "Yesterday, I washed my hands more than I would usually wash them before the COVID-19 crisis started."

Response options were: Strongly disagree (1); Disagree (2); Somewhat disagree (3); Neither agree nor disagree (4); Somewhat agree (5); Agree (6); Strongly agree (7)

- Disinfect

To what extent do you agree or disagree with the following statement: "Yesterday, I made sure to disinfect any packages, foods, etc. that I brought into my house."

Response options were: Does not apply to me (I did not leave my house yesterday) (8); Strongly disagree (1); Disagree (2); Somewhat disagree (3); Neither agree nor disagree (4); Somewhat agree (5); Agree (6); Strongly agree (7)

- Hand Washing Times

Yesterday, how many times approximately did you wash your hands?

Response options ranged from “Never (0)” to “More than 20 times (21)”

## **MODERATORS** (all moderators were measured in Part 1 of the study)

- Distancing History

How many days ago did you first start practising social distancing? Please select the appropriate number below. Social distancing can involve any of the following:

- - working from home, where possible (e.g., if you are not considered an "essential worker" in your country);
  - avoiding large gatherings and gatherings in smaller public spaces such as cinemas, restaurants, etc.;
  - avoiding non-essential use of public transport;
  - avoiding gatherings with friends and family, unless you live together with them;
  - self-isolating because you are experiencing symptoms of COVID-19 or because you are among the vulnerable population;
  - leaving your house only for essential activities (shopping for groceries or medications; or work if you are considered an "essential worker" in your country)

Response options were: 0 (I do not practise social distancing) (0); 1 day ago (1); … ; More than 100 days ago (101)

- Living Situation

To what extent do you agree or disagree with the following statement: "My living situation allows me to sufficiently self-distance from others if necessary".

Response options were: Strongly disagree (1); Disagree (2); Somewhat disagree (3); Neither agree nor disagree (4); Somewhat agree (5); Agree (6); Strongly agree (7)

- Economic Reasons

To what extent do you agree or disagree with the following statement: "I cannot afford to practise self-distancing for economic reasons."

Response options were: Strongly disagree (1); Disagree (2); Somewhat disagree (3); Neither agree nor disagree (4); Somewhat agree (5); Agree (6); Strongly agree (7)

## **MEDIATORS** (all mediators were measured in Part 1 of the study)

- Serious Disease

To what extent do you agree or disagree with the following statement: "COVID-19 poses a serious risk for all humans."

Response options were: Strongly disagree (1); Disagree (2); Somewhat disagree (3); Neither agree nor disagree (4); Somewhat agree (5); Agree (6); Strongly agree (7)

- Health Concern

To what extent do you agree or disagree with the following statement: "My health could be severely affected if I were to catch COVID-19".

Response options were: Strongly disagree (1); Disagree (2); Somewhat disagree (3); Neither agree nor disagree (4); Somewhat agree (5); Agree (6); Strongly agree (7)

- Concern Close Ones

To what extent do you agree or disagree with the following statement: "I feel concerned for my close ones who are vulnerable and could get COVID-19".

Response options were: Strongly disagree (1); Disagree (2); Somewhat disagree (3); Neither agree nor disagree (4); Somewhat agree (5); Agree (6); Strongly agree (7)

- Concern Vulnerable Others

To what extent do you agree or disagree with the following statement: "I feel concerned for anyone who is vulnerable and could get COVID-19".

Response options were: Strongly disagree (1); Disagree (2); Somewhat disagree (3); Neither agree nor disagree (4); Somewhat agree (5); Agree (6); Strongly agree (7)

- Economic Concern

To what extent do you agree or disagree with the following statement: "I feel concerned about how COVID-19 will impact our economy".

Response options were: Strongly disagree (1); Disagree (2); Somewhat disagree (3); Neither agree nor disagree (4); Somewhat agree (5); Agree (6); Strongly agree (7)

- Meaningful Time

To what extent do you agree or disagree with the following statement: "Going forward, I feel that the time that I spend at home during the period of COVID-19 pandemic will be meaningful".

Response options were: Strongly disagree (1); Disagree (2); Somewhat disagree (3); Neither agree nor disagree (4); Somewhat agree (5); Agree (6); Strongly agree (7)

- Knowledge

How would you rate your current knowledge of COVID-19?

Response options were: Not knowledgeable at all (1); Slightly knowledgeable (2); Moderately knowledgeable (3); Very knowledgeable (4); Extremely knowledgeable (5)

- Future Intentions

To what extent do you agree or disagree with the following statement: "Going forward, I am intending to undertake behaviours that could reduce the spread of COVID-19 (e.g., social distancing, hand washing, keeping appropriate distance from others when I am outside, etc.).

Response options were: Strongly disagree (1); Disagree (2); Somewhat disagree (3); Neither agree nor disagree (4); Somewhat agree (5); Agree (6); Strongly agree (7)

## **COVARIATES** (all covariates were measured in Part 1 of the study)

- Household Income ^[[4]](#footnote-4)^

Which one of these phrases comes closest to your own feelings about your household's income these days?

Response options were: Finding it very difficult on present income (1); Finding it difficult on present income (2); Getting by on present income (3); Living comfortably on present income (4); Living very comfortably on present income (5)

- Education*

Which option below best describes your highest education level?

Response options were: No formal qualifications (0); Secondary Education (1); Undergraduate degree (2); Graduate degree (3); Doctoral degree (4); Professional qualification (5)

- Prior Home

Prior to the COVID-19 crisis, how many days per week did you typically spend at home (working from home or otherwise)?

Response options were: Before COVID-19 crisis, I would rarely spend a full day at home (0); Before COVID-19 crisis, I would typically spend 1 day a week at home (1); … ; Before COVID-19 crisis, I would typically spend the full week at home (7)

- Household

How many people, in addition to you, currently live in your household?

Response options were: 0 (I live on my own) (0); 1 (1); … ; More than 10 (11)

- Property*

Which option below best describes your living situation?

Response options were: I live in a single room in shared accommodation (0); I live in a studio flat (1); I live in a one bedroom property (2); I live in a two bedroom property (3); I live in a three bedroom property (4); I live in a property that has more than three bedrooms (5)

- Garden*

Do you have access to an outdoor space (e.g. garden) that you can use whenever you want without being in danger of coming in contact with other people?

Response options were: No (0); Yes (1)

- Key Worker*

Are you considered a key worker (as defined by your country)?

Response options were: No (0); Yes (1)

- Gender*

What is your gender?

Response options were: Male (0); Female (1); Other (2)

- Age

What is your age in years (please write the appropriate number)?

- Country*

UK (0); US = (1)

- On Time*

In this variable we computed whether participants completed the survey for Part 2 “On Time” (on the second day after they completed Part 1) or not (i.e. later).

Yes (0); No (1)

## **EXCLUSION CRITERIA ITEMS**

- Instructed Response Check 1 (measured in Part 1 of the Study)

Please select "Strongly disagree" for this item.

Response options were: Strongly disagree (1); Disagree (0); Somewhat disagree (0); Neither agree nor disagree (0); Somewhat agree (0); Agree (0); Strongly agree (0)

- Instructed Response Check 2 (measured in Part 2 of the Study)

Please select "Somewhat agree" for this item.

Response options were: Strongly disagree (1); Disagree (2); Somewhat disagree (3); Neither agree nor disagree (4); Somewhat agree (5); Agree (6); Strongly agree (7)

- Seriousness check (measured in both Part 1 and 2 of the study)

It would be very helpful if you could tell us at this point whether you have taken part in this study seriously, so that we can use your answers for our scientific analysis, or you were just clicking through without reading the instructions and survey questions? Please answer honestly.

Response options were: I have taken part seriously. (1); I have not taken part seriously, please throw my data away. (0)

- Agreement (measured in part 2 of the study)

Please confirm if you agree for your responses in this study to be used in our scientific analyses.

Response options were: No, I do not agree (1); Yes, I agree for my responses to be used in your scientific analyses (2)

- Captcha (participants had to accurately answer this question at the end of both Part 1 and 2 in order to finish the survey)

To finish the survey, please complete the captcha below to show that you are not a robot.


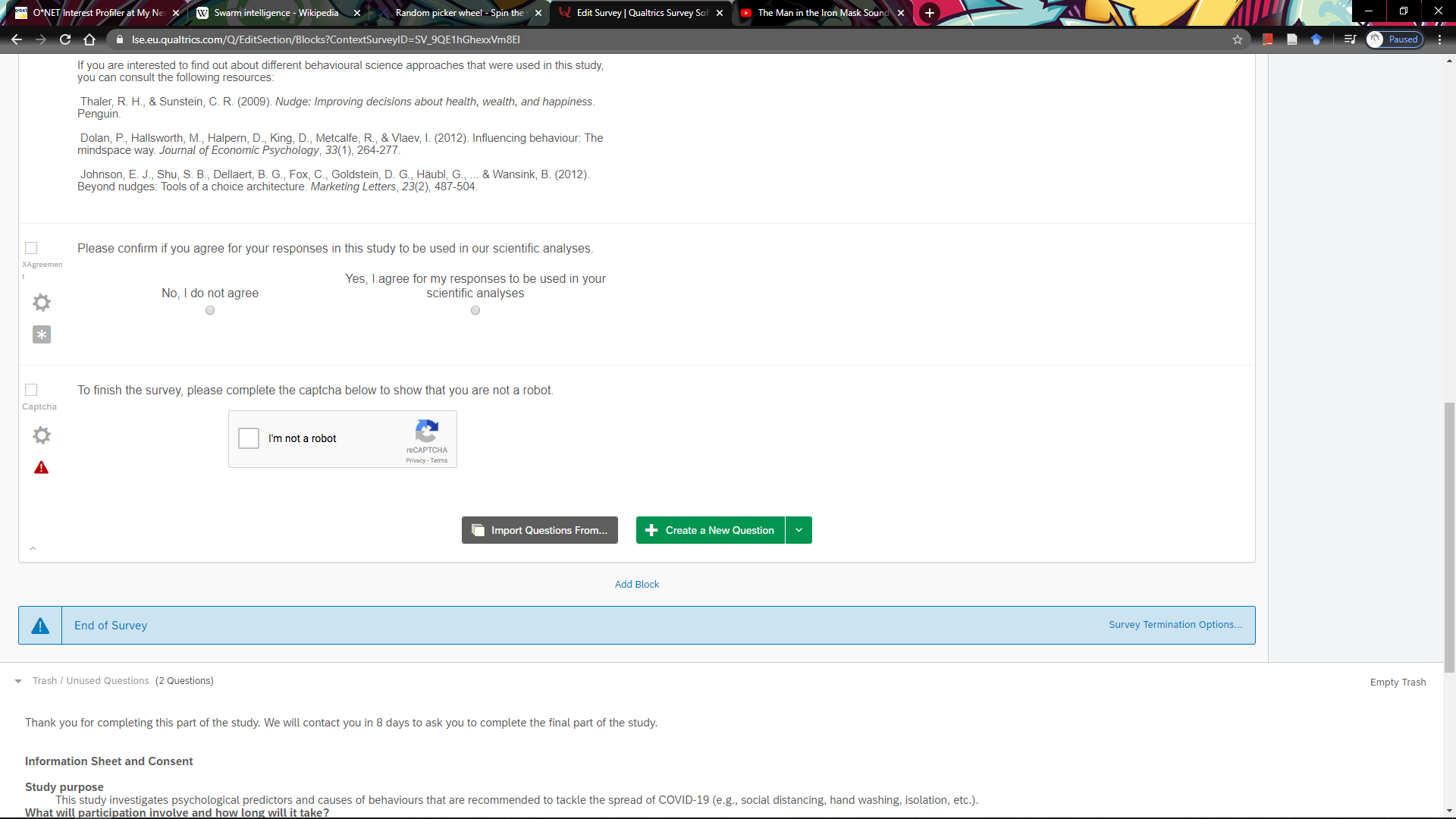


# **Descriptive Statistics**

Table S1

*Descriptive Statistics for All Variables (Dependent Variables, Moderators, Mediators, and Covariates) Measured in the Present Study*

| **Variable** | **N of observations** | **Minimum** | **Maximum** | **Mean** | **Std. Deviation** |
| --- | --- | --- | --- | --- | --- |
| 1 | 2637 | 1.0 | 5.0 | 4.699 | 0.604 |
| 2 | 2637 | 0.0 | 11.0 | 0.386 | 0.799 |
| 3 | 2637 | 0.0 | 11.0 | 0.430 | 0.986 |
| 4 | 2637 | 0.0 | 10.0 | 0.456 | 0.732 |
| 5 | 2637 | 0.0 | 10.0 | 0.462 | 0.706 |
| 6 | 2637 | 0.0 | 1.0 | 0.036 | 0.186 |
| 7 | 2636 | 0.0 | 1.0 | 0.030 | 0.174 |
| 8 | 1576 | 1.0 | 7.0 | 6.490 | 0.975 |
| 9 | 2637 | 1.0 | 7.0 | 5.745 | 1.434 |
| 10 | 1769 | 1.0 | 7.0 | 4.540 | 2.132 |
| 11 | 2637 | 0.0 | 21.0 | 8.931 | 4.707 |
| 12 | 2635 | 0.0 | 101.0 | 21.130 | 9.398 |
| 13 | 2637 | 1.0 | 7.0 | 5.671 | 1.469 |
| 14 | 2637 | 1.0 | 7.0 | 2.290 | 1.470 |
| 15 | 2637 | 1.0 | 7.0 | 6.092 | 1.228 |
| 16 | 2637 | 1.0 | 7.0 | 5.763 | 1.312 |
| 17 | 2637 | 1.0 | 7.0 | 6.439 | 0.944 |
| 18 | 2637 | 1.0 | 7.0 | 6.378 | 0.863 |
| 19 | 2637 | 1.0 | 7.0 | 6.246 | 1.026 |
| 20 | 2635 | 1.0 | 7.0 | 5.550 | 1.344 |
| 21 | 2637 | 1.0 | 5.0 | 3.418 | 0.706 |
| 22 | 2637 | 1.0 | 7.0 | 6.651 | 0.705 |
| 23 | 2637 | 1.0 | 5.0 | 3.096 | 0.997 |
| 24 | 2637 | 0.0 | 5.0 | 2.058 | 1.092 |
| 25 | 2634 | 0.0 | 7.0 | 3.120 | 2.446 |
| 26 | 2637 | 0.0 | 11.0 | 2.043 | 1.551 |
| 27 | 2637 | 0.0 | 5.0 | 3.670 | 1.125 |
| 28 | 2637 | 0.0 | 1.0 | 0.833 | 0.373 |
| 29 | 2637 | 0.0 | 1.0 | 0.224 | 0.417 |
| 30 | 2637 | 0.0 | 2.0 | 0.520 | 0.503 |
| 31 | 2635 | 18.0 | 87.0 | 45.59 | 15.659 |
| 32 | 2637 | 0.0 | 1.0 | 0.489 | 0.500 |
| 33 | 2637 | 0.0 | 1.0 | 0.111 | 0.314 |
| *Note.* The variables to which the numbers correspond are as follows: **DEPENDENT VARIABLES**: 1 – General Distancing; 2 – Going Out Times; 3 – Going Out Hours; 4 – Physical Fitness Times; 5 – Physical Fitness Hours; 6 – Out Family Friends; 7 – Social Gatherings; 8 – Keeping Distance; 9 – Relative Hand Washing; 10 – Disinfect; 11 – Hand Washing Times; **MODERATORS:** 12 – Distancing History; 13 – Living Situation; 14 – Economic Reasons; **MEDIATORS**: 15 – Serious Disease; 16 – Health Concern; 17 – Concern Close Ones; 18 – Concern Vulnerable Others; 19 – Economic Concern; 20 – Meaningful Time; 21 – Knowledge; 22 – Future Intentions; **COVARIATES**: 23 – Household Income; 24 – Education (No formal qualifications = 0; Secondary Education = 1; Undergraduate degree = 2; Graduate degree = 3; Doctoral degree = 4; Professional qualification = 5); 25 – Prior Home; 26 – Household; 27 – Property (I live in a single room in shared accommodation = 0; I live in a studio flat = 1; I live in a one bedroom property = 2; I live in a two bedroom property = 3 = I live in a three bedroom property = 4; I live in a property that has more than three bedrooms = 5); 28 – Garden (No = 0; Yes = 1); 29 – Key Worker (No = 0; Yes = 1); 30 – Gender (Male = 0; Female = 1; Other = 2); 31 – Age; 32 – Country (UK = 0; US = 1); 33 – On Time (0 = Yes; 1 = No). | | | | | |

# **Zero-order Correlations Between All Continuous and Dichotomous Categorical Variables**

Table S2

*Zero-order Pearson Correlations Between All Continuous and Dichotomous Categorical Variables (Dependent Variables, Moderators, Mediators, and Covariates) Measured in the Present Study*

|  | | | | | | | | | | | | | | | | | | | | | | | | | | | | | | | | | | | | | | | | | | | | | | | | | | | | | | | | | | | | | |  |
| --- | --- | --- | --- | --- | --- | --- | --- | --- | --- | --- | --- | --- | --- | --- | --- | --- | --- | --- | --- | --- | --- | --- | --- | --- | --- | --- | --- | --- | --- | --- | --- | --- | --- | --- | --- | --- | --- | --- | --- | --- | --- | --- | --- | --- | --- | --- | --- | --- | --- | --- | --- | --- | --- | --- | --- | --- | --- | --- | --- | --- | --- | --- |
|  | | **1** | | **2** | | **3** | | **4** | | **5** | | **6** | | **7** | | **8** | | **9** | | **10** | | **11** | | **12** | | **13** | | **14** | | **15** | | **16** | | **17** | | **18** | | **19** | | **20** | | **21** | | **22** | | **23** | | **24** | | **25** | | **26** | | **27** | | **28** | | **29** | | **30** | | **31** |
| **1** |  | — |  |  |  |  |  |  |  |  |  |  |  |  |  |  |  |  |  |  |  |  |  |  |  |  |  |  |  |  |  |  |  |  |  |  |  |  |  |  |  |  |  |  |  |  |  |  |  |  |  |  |  |  |  |  |  |  |  |  |  |  |
| **2** |  | -0.251 | *** | — |  |  |  |  |  |  |  |  |  |  |  |  |  |  |  |  |  |  |  |  |  |  |  |  |  |  |  |  |  |  |  |  |  |  |  |  |  |  |  |  |  |  |  |  |  |  |  |  |  |  |  |  |  |  |  |  |  |  |
| **3** |  | -0.253 | *** | 0.595 | *** | — |  |  |  |  |  |  |  |  |  |  |  |  |  |  |  |  |  |  |  |  |  |  |  |  |  |  |  |  |  |  |  |  |  |  |  |  |  |  |  |  |  |  |  |  |  |  |  |  |  |  |  |  |  |  |  |  |
| **4** |  | -0.146 | *** | 0.484 | *** | 0.299 | *** | — |  |  |  |  |  |  |  |  |  |  |  |  |  |  |  |  |  |  |  |  |  |  |  |  |  |  |  |  |  |  |  |  |  |  |  |  |  |  |  |  |  |  |  |  |  |  |  |  |  |  |  |  |  |  |
| **5** |  | -0.133 | *** | 0.493 | *** | 0.377 | *** | 0.753 | *** | — |  |  |  |  |  |  |  |  |  |  |  |  |  |  |  |  |  |  |  |  |  |  |  |  |  |  |  |  |  |  |  |  |  |  |  |  |  |  |  |  |  |  |  |  |  |  |  |  |  |  |  |  |
| **6** |  | -0.230 | *** | 0.215 | *** | 0.248 | *** | 0.055 | ** | 0.078 | *** | — |  |  |  |  |  |  |  |  |  |  |  |  |  |  |  |  |  |  |  |  |  |  |  |  |  |  |  |  |  |  |  |  |  |  |  |  |  |  |  |  |  |  |  |  |  |  |  |  |  |  |
| **7** |  | -0.131 | *** | 0.088 | *** | 0.088 | *** | 0.047 | * | 0.040 | * | 0.270 | *** | — |  |  |  |  |  |  |  |  |  |  |  |  |  |  |  |  |  |  |  |  |  |  |  |  |  |  |  |  |  |  |  |  |  |  |  |  |  |  |  |  |  |  |  |  |  |  |  |  |
| **8** |  | 0.432 | *** | -0.137 | *** | -0.147 | *** | -0.004 |  | 0.003 |  | -0.209 | *** | -0.120 | *** | — |  |  |  |  |  |  |  |  |  |  |  |  |  |  |  |  |  |  |  |  |  |  |  |  |  |  |  |  |  |  |  |  |  |  |  |  |  |  |  |  |  |  |  |  |  |  |
| **9** |  | 0.119 | *** | -0.001 |  | 0.029 |  | 0.056 | ** | 0.047 | * | -0.047 | * | -0.055 | ** | 0.248 | *** | — |  |  |  |  |  |  |  |  |  |  |  |  |  |  |  |  |  |  |  |  |  |  |  |  |  |  |  |  |  |  |  |  |  |  |  |  |  |  |  |  |  |  |  |  |
| **10** |  | 0.213 | *** | -0.109 | *** | -0.082 | *** | -0.044 |  | -0.067 | ** | -0.102 | *** | -0.050 | * | 0.242 | *** | 0.365 | *** | — |  |  |  |  |  |  |  |  |  |  |  |  |  |  |  |  |  |  |  |  |  |  |  |  |  |  |  |  |  |  |  |  |  |  |  |  |  |  |  |  |  |  |
| **11** |  | 0.118 | *** | -0.046 | * | -0.040 | * | -0.027 |  | -0.018 |  | -0.064 | *** | -0.054 | ** | 0.121 | *** | 0.290 | *** | 0.253 | *** | — |  |  |  |  |  |  |  |  |  |  |  |  |  |  |  |  |  |  |  |  |  |  |  |  |  |  |  |  |  |  |  |  |  |  |  |  |  |  |  |  |
| **12** |  | 0.151 | *** | -0.091 | *** | -0.074 | *** | -0.055 | ** | -0.072 | *** | -0.057 | ** | -0.009 |  | 0.142 | *** | 0.063 | ** | 0.174 | *** | 0.090 | *** | — |  |  |  |  |  |  |  |  |  |  |  |  |  |  |  |  |  |  |  |  |  |  |  |  |  |  |  |  |  |  |  |  |  |  |  |  |  |  |
| **13** |  | 0.056 | ** | -0.049 | * | -0.000 |  | -0.008 |  | 0.006 |  | -0.036 |  | -0.021 |  | 0.096 | *** | 0.016 |  | 0.055 | * | -0.015 |  | 0.054 | ** | — |  |  |  |  |  |  |  |  |  |  |  |  |  |  |  |  |  |  |  |  |  |  |  |  |  |  |  |  |  |  |  |  |  |  |  |  |
| **14** |  | -0.188 | *** | 0.072 | *** | 0.098 | *** | 0.016 |  | 0.004 |  | 0.078 | *** | 0.069 | *** | -0.189 | *** | -0.087 | *** | -0.048 | * | -0.054 | ** | -0.064 | *** | -0.291 | *** | — |  |  |  |  |  |  |  |  |  |  |  |  |  |  |  |  |  |  |  |  |  |  |  |  |  |  |  |  |  |  |  |  |  |  |
| **15** |  | 0.199 | *** | -0.112 | *** | -0.096 | *** | -0.089 | *** | -0.095 | *** | -0.081 | *** | -0.085 | *** | 0.264 | *** | 0.261 | *** | 0.280 | *** | 0.125 | *** | 0.077 | *** | 0.043 | * | -0.111 | *** | — |  |  |  |  |  |  |  |  |  |  |  |  |  |  |  |  |  |  |  |  |  |  |  |  |  |  |  |  |  |  |  |  |
| **16** |  | 0.140 | *** | -0.102 | *** | -0.100 | *** | -0.080 | *** | -0.081 | *** | -0.083 | *** | -0.046 | * | 0.202 | *** | 0.252 | *** | 0.253 | *** | 0.119 | *** | 0.080 | *** | 0.028 |  | -0.081 | *** | 0.545 | *** | — |  |  |  |  |  |  |  |  |  |  |  |  |  |  |  |  |  |  |  |  |  |  |  |  |  |  |  |  |  |  |
| **17** |  | 0.185 | *** | -0.107 | *** | -0.093 | *** | -0.091 | *** | -0.076 | *** | -0.086 | *** | -0.065 | *** | 0.218 | *** | 0.244 | *** | 0.224 | *** | 0.151 | *** | 0.055 | ** | -0.016 |  | -0.097 | *** | 0.481 | *** | 0.442 | *** | — |  |  |  |  |  |  |  |  |  |  |  |  |  |  |  |  |  |  |  |  |  |  |  |  |  |  |  |  |
| **18** |  | 0.196 | *** | -0.102 | *** | -0.087 | *** | -0.074 | *** | -0.068 | *** | -0.092 | *** | -0.094 | *** | 0.247 | *** | 0.220 | *** | 0.216 | *** | 0.164 | *** | 0.067 | *** | 0.039 | * | -0.114 | *** | 0.466 | *** | 0.346 | *** | 0.584 | *** | — |  |  |  |  |  |  |  |  |  |  |  |  |  |  |  |  |  |  |  |  |  |  |  |  |  |  |
| **19** |  | 0.079 | *** | -0.009 |  | 0.012 |  | -0.002 |  | -0.012 |  | 0.019 |  | -0.032 |  | 0.112 | *** | 0.122 | *** | 0.073 | ** | 0.039 | * | 0.001 |  | 0.054 | ** | 0.015 |  | 0.151 | *** | 0.145 | *** | 0.155 | *** | 0.246 | *** | — |  |  |  |  |  |  |  |  |  |  |  |  |  |  |  |  |  |  |  |  |  |  |  |  |
| **20** |  | 0.112 | *** | -0.083 | *** | -0.032 |  | -0.052 | ** | -0.050 | * | -0.037 |  | -0.052 | ** | 0.140 | *** | 0.138 | *** | 0.176 | *** | 0.076 | *** | 0.057 | ** | 0.121 | *** | -0.138 | *** | 0.279 | *** | 0.199 | *** | 0.253 | *** | 0.306 | *** | 0.109 | *** | — |  |  |  |  |  |  |  |  |  |  |  |  |  |  |  |  |  |  |  |  |  |  |
| **21** |  | 0.024 |  | -0.024 |  | -0.007 |  | 0.020 |  | 0.025 |  | 0.018 |  | 0.005 |  | 0.037 |  | 0.128 | *** | 0.149 | *** | 0.048 | * | 0.071 | *** | 0.073 | *** | -0.016 |  | 0.090 | *** | 0.097 | *** | 0.081 | *** | 0.131 | *** | 0.087 | *** | 0.120 | *** | — |  |  |  |  |  |  |  |  |  |  |  |  |  |  |  |  |  |  |  |  |
| **22** |  | 0.356 | *** | -0.157 | *** | -0.112 | *** | -0.088 | *** | -0.081 | *** | -0.133 | *** | -0.098 | *** | 0.399 | *** | 0.256 | *** | 0.217 | *** | 0.182 | *** | 0.159 | *** | 0.065 | *** | -0.191 | *** | 0.385 | *** | 0.312 | *** | 0.407 | *** | 0.398 | *** | 0.131 | *** | 0.251 | *** | 0.096 | *** | — |  |  |  |  |  |  |  |  |  |  |  |  |  |  |  |  |  |  |
| **23** |  | 0.064 | ** | 0.047 | * | 0.013 |  | 0.081 | *** | 0.096 | *** | -0.013 |  | -0.061 | ** | 0.060 | * | 0.025 |  | -0.071 | ** | -0.013 |  | -0.033 |  | 0.183 | *** | -0.359 | *** | -0.040 | * | -0.070 | *** | -0.016 |  | -0.024 |  | -0.041 | * | 0.078 | *** | 0.024 |  | 0.039 | * | — |  |  |  |  |  |  |  |  |  |  |  |  |  |  |  |  |
| **24** |  | 0.043 | * | -0.048 | * | -0.047 | * | -0.040 | * | -0.041 | * | 0.006 |  | 0.031 |  | 0.043 |  | -0.007 |  | 0.039 |  | 0.053 | ** | 0.134 | *** | 0.115 | *** | -0.154 | *** | 0.001 |  | 0.102 | *** | -0.018 |  | -0.016 |  | -0.020 |  | -0.012 |  | 0.015 |  | 0.005 |  | -0.015 |  | — |  |  |  |  |  |  |  |  |  |  |  |  |  |  |
| **25** |  | -0.054 | ** | 0.000 |  | -0.005 |  | -0.034 |  | -0.041 | * | 0.006 |  | 0.047 | * | -0.059 | * | -0.061 | ** | 0.038 |  | 0.021 |  | -0.012 |  | -0.240 | *** | 0.127 | *** | -0.014 |  | -0.109 | *** | 0.005 |  | 0.022 |  | -0.040 | * | 0.033 |  | -0.020 |  | -0.071 | *** | -0.037 |  | -0.124 | *** | — |  |  |  |  |  |  |  |  |  |  |  |  |
| **26** |  | 0.018 |  | -0.003 |  | -0.010 |  | 0.049 | * | 0.044 | * | -0.012 |  | 0.027 |  | 0.048 |  | 0.047 | * | 0.035 |  | 0.020 |  | -0.046 | * | 0.138 | *** | -0.152 | *** | -0.005 |  | 0.022 |  | 0.025 |  | 0.038 |  | 0.023 |  | 0.033 |  | 0.007 |  | 0.010 |  | 0.111 | *** | 0.066 | *** | 0.146 | *** | — |  |  |  |  |  |  |  |  |  |  |
| **27** |  | -0.164 | *** | 0.028 |  | 0.038 |  | 0.016 |  | 0.022 |  | 0.047 | * | -0.002 |  | -0.102 | *** | -0.017 |  | -0.066 | ** | -0.006 |  | -0.090 | *** | -0.056 | ** | 0.163 | *** | -0.046 | * | -0.047 | * | -0.013 |  | -0.012 |  | -0.041 | * | -0.003 |  | 0.020 |  | -0.037 |  | 0.006 |  | -0.271 | *** | 0.086 | *** | 0.051 | ** | — |  |  |  |  |  |  |  |  |
| **28** |  | 0.126 | *** | -0.099 | *** | -0.102 | *** | -0.099 | *** | -0.086 | *** | -0.058 | ** | -0.006 |  | 0.123 | *** | 0.026 |  | 0.053 | * | 0.203 | *** | 0.065 | *** | -0.001 |  | -0.066 | *** | 0.144 | *** | 0.048 | * | 0.117 | *** | 0.159 | *** | 0.078 | *** | 0.084 | *** | 0.016 |  | 0.176 | *** | -0.006 |  | 0.074 | *** | 0.006 |  | 0.047 | * | -0.026 |  | — |  |  |  |  |  |  |
| **29** |  | 0.094 | *** | -0.033 |  | -0.028 |  | -0.016 |  | -0.017 |  | -0.035 |  | -0.030 |  | 0.150 | *** | 0.144 | *** | 0.075 | ** | 0.115 | *** | 0.001 |  | 0.152 | *** | -0.196 | *** | 0.057 | ** | 0.260 | *** | 0.018 |  | 0.083 | *** | 0.125 | *** | 0.054 | ** | 0.080 | *** | 0.108 | *** | 0.043 | * | 0.309 | *** | -0.296 | *** | 0.212 | *** | -0.110 | *** | 0.019 |  | — |  |  |  |  |
| **30** |  | -0.160 | *** | -0.015 |  | 0.004 |  | 0.007 |  | -0.023 |  | 0.055 | ** | 0.078 | *** | -0.126 | *** | -0.042 | * | 0.131 | *** | -0.105 | *** | 0.159 | *** | 0.101 | *** | 0.081 | *** | -0.030 |  | -0.028 |  | -0.079 | *** | -0.052 | ** | 0.004 |  | 0.011 |  | 0.107 | *** | -0.104 | *** | -0.096 | *** | 0.072 | *** | 0.028 |  | -0.010 |  | 0.006 |  | -0.008 |  | -0.031 |  | — |  |  |
| **31** |  | -0.052 | ** | 0.005 |  | 0.048 | * | -0.023 |  | -0.003 |  | 0.029 |  | 0.020 |  | -0.079 | ** | -0.053 | ** | -0.023 |  | -0.007 |  | 0.004 |  | -0.021 |  | 0.045 | * | 0.000 |  | -0.016 |  | 0.006 |  | -0.002 |  | -0.018 |  | 0.023 |  | -0.009 |  | -0.028 |  | 0.001 |  | -0.024 |  | 0.030 |  | -0.027 |  | 0.045 | * | 0.028 |  | -0.146 | *** | 0.104 | *** | — |
|  | | | | | | | | | | | | | | | | | | | | | | | | | | | | | | | | | | | | | | | | | | | | | | | | | | | | | | | | | | | | | |  |
| *Note.* * p < .05, ** p < .01, *** p < .001. All variables are expressed as numbers for formatting purposes. The variables to which the numbers correspond are: **DEPENDENT VARIABLES**: 1 – General Distancing; 2 – Going Out Times; 3 – Going Out Hours; 4 – Physical Fitness Times; 5 – Physical Fitness Hours; 6 – Out Family Friends; 7 – Social Gatherings; 8 – Keeping Distance; 9 – Relative Hand Washing; 10 – Disinfect; 11 – Hand Washing Times; **MODERATORS:** 12 – Distancing History; 13 – Living Situation; 14 – Economic Reasons; **MEDIATORS**: 15 – Serious Disease; 16 – Health Concern; 17 – Concern Close Ones; 18 – Concern Vulnerable Others; 19 – Economic Concern; 20 – Meaningful Time; 21 – Knowledge; 22 – Future Intentions; **COVARIATES**: 23 – Household Income; 24 – Prior Home; 25 – Household; 26 – Garden (No = 0; Yes = 1); 27 – Key Worker (No = 0; Yes = 1); 28 – Gender (Male = 0; Female = 1; There were only five participants who responded with “Other” and their responses were not used in the correlation analyses); 29 – Age; 30 – Country (UK = 0; US = 1); 31 – On Time (0 = Yes; 1 = No). | | | | | | | | | | | | | | | | | | | | | | | | | | | | | | | | | | | | | | | | | | | | | | | | | | | | | | | | | | | | | |  |

# **Main Effects: The Influence of the Intervention Conditions on the Dependent Variables**

In the tables below, we present regression analyses in which we tested the effect of the four intervention conditions (Letter, Meaningful Activity, Economy, and Information) compared to the control condition on the 11 dependent variables measured in this study: general distancing, going out times, going out hours, physical fitness times, physical fitness hours, out family friends, social gatherings, keeping distance, relative hand washing, disinfect, and hand washing times.

Table S3

*Multiple Linear Regression for the Influence of the Intervention Conditions on General Distancing*

|  | | | | | | **95% CI for *b*** | |
| --- | --- | --- | --- | --- | --- | --- | --- |
| **Condition** | ***b*** | **Std. Error** | ***β*** | ***t*** | ***p*** | **Lower** | **Upper** |
| (Constant) | 4.702 | 0.026 |  | 182.353 | <.001 | 4.651 | 4.752 |
| Letter | -0.022 | 0.038 | -0.014 | -0.579 | .562 | -0.096 | 0.052 |
| Meaningful Activity | -0.007 | 0.037 | -0.004 | -0.178 | .858 | -0.079 | 0.066 |
| Economy | -0.010 | 0.037 | -0.007 | -0.267 | .789 | -0.082 | 0.062 |
| Information | 0.021 | 0.037 | 0.014 | 0.578 | .563 | -0.051 | 0.093 |
| *Note*. Model *R*^2^ = 0.001. Control condition is the reference category. | | | | | | | |

Table S4

*Multiple Linear Regression for the Influence of the Intervention Conditions on Going Out Times*

|  | | | | | | **95% CI for *b*** | |
| --- | --- | --- | --- | --- | --- | --- | --- |
| **Condition** | ***b*** | **Std. Error** | ***β*** | ***t*** | ***p*** | **Lower** | **Upper** |
| (Constant) | 0.429 | .034 |  | 12.590 | <.001 | 0.362 | 0.496 |
| Letter | -0.071 | .050 | -0.034 | -1.428 | .154 | -0.169 | 0.027 |
| Meaningful Activity | -0.044 | .049 | -0.022 | -0.909 | .363 | -0.140 | 0.051 |
| Economy | -0.062 | .048 | -0.031 | -1.275 | .203 | -0.157 | 0.033 |
| Information | -0.042 | .048 | -0.021 | -0.868 | .385 | -0.137 | 0.053 |
| *Note*. Model *R*^2^ = 0.001. Control condition is the reference category. | | | | | | | |

Table S5

*Multiple Linear Regression for the Influence of the Intervention Conditions on Going Out Hours*

|  | | | | | | **95% CI for *b*** | |
| --- | --- | --- | --- | --- | --- | --- | --- |
| **Condition** | ***b*** | **Std. Error** | ***β*** | ***t*** | ***p*** | **Lower** | **Upper** |
| (Constant) | 0.498 | 0.042 |  | 11.856 | <.001 | 0.416 | 0.581 |
| Letter | -0.126 | 0.062 | -0.049 | -2.041 | 0.041† | -0.247 | -0.005 |
| Meaningful Activity | -0.058 | 0.060 | -0.024 | -0.968 | 0.333 | -0.176 | 0.060 |
| Economy | -0.066 | 0.060 | -0.027 | -1.103 | 0.270 | -0.183 | 0.051 |
| Information | -0.096 | 0.060 | -0.040 | -1.618 | 0.106 | -0.213 | 0.020 |
| *Note*. Model *R*^2^ = 0.002. Control condition is the reference category. Symbol † indicates the initially significant results that stopped being significant after the false discovery rate (FDR) correction was applied. | | | | | | | |

Table S6

*Multiple Linear Regression for the Influence of the Intervention Conditions on Physical Fitness Times*

|  | | | | | | **95% CI for *b*** | |
| --- | --- | --- | --- | --- | --- | --- | --- |
| **Condition** | ***b*** | **Std. Error** | ***β*** | ***t*** | ***p*** | **Lower** | **Upper** |
| (Constant) | 0.493 | 0.031 |  | 15.787 | <.001 | 0.432 | 0.554 |
| Letter | -0.041 | 0.046 | -0.022 | -0.892 | .372 | -0.131 | 0.049 |
| Meaningful Activity | -0.045 | 0.045 | -0.025 | -1.010 | .313 | -0.133 | 0.042 |
| Economy | -0.081 | 0.044 | -0.045 | -1.823 | .068 | -0.168 | 0.006 |
| Information | -0.019 | 0.044 | -0.011 | -0.437 | .662 | -0.106 | 0.067 |
| *Note*. Model *R*^2^ = 0.001. Control condition is the reference category. | | | | | | | |

Table S7

*Multiple Linear Regression for the Influence of the Intervention Conditions on Physical Fitness Hours*

|  | | | | | | **95% CI for *b*** | |
| --- | --- | --- | --- | --- | --- | --- | --- |
| **Condition** | ***b*** | **Std. Error** | ***β*** | ***t*** | ***p*** | **Lower** | **Upper** |
| (Constant) | 0.504 | 0.030 |  | 16.716 | <.001 | 0.445 | 0.563 |
| Letter | -0.048 | 0.044 | -0.026 | -1.077 | .282 | -0.134 | 0.039 |
| Meaningful Activity | -0.035 | 0.043 | -0.020 | -0.813 | .416 | -0.120 | 0.049 |
| Economy | -0.060 | 0.043 | -0.034 | -1.406 | .160 | -0.144 | 0.024 |
| Information | -0.065 | 0.043 | -0.037 | -1.524 | .128 | -0.149 | 0.019 |
| *Note*. Model *R*^2^ = 0.001. Control condition is the reference category. | | | | | | | |

Table S8

*Multiple Logistic Regression for the Influence of the Intervention Conditions on Out Family Friends*

|  | | | | | | **95% CI for Odds Ratio** | |
| --- | --- | --- | --- | --- | --- | --- | --- |
| **Condition** | ***b*** | **Std. Error** | **Wald** | ***p*** | **Odds Ratio** | **Lower** | **Upper** |
| (Constant) | -3.178 | 0.218 | 213.313 | <.001 | 0.042 |  |  |
| Letter | -0.185 | 0.335 | 0.305 | .581 | 0.831 | 0.431 | 1.602 |
| Meaningful Activity | -0.160 | 0.324 | 0.244 | .621 | 0.852 | 0.452 | 1.607 |
| Economy | 0.067 | 0.305 | 0.049 | .825 | 1.070 | 0.589 | 1.943 |
| Information | -0.320 | 0.334 | 0.918 | .338 | 0.726 | 0.377 | 1.398 |
| *Note*. Model *R*^2^ = 0.003 (Nagelkerke), Model *χ*^2^ (4) = 1.772, *p* = .778. Control condition is the reference category. | | | | | | | |

Table S9

*Multiple Logistic Regression for the Influence of the Intervention Conditions on Social Gatherings*

|  | | | | | | **95% CI for Odds Ratio** | |
| --- | --- | --- | --- | --- | --- | --- | --- |
| **Condition** | ***b*** | **Std. Error** | **Wald** | ***p*** | **Odds Ratio** | **Lower** | **Upper** |
| (Constant) | -3.226 | 0.223 | 210.265 | <.001 | 0.040 |  |  |
| Letter | -0.274 | 0.351 | 0.612 | .434 | 0.760 | 0.382 | 1.512 |
| Meaningful Activity | -0.713 | 0.389 | 3.358 | .067 | 0.490 | 0.229 | 1.051 |
| Economy | 0.322 | 0.295 | 1.192 | .275 | 1.380 | 0.774 | 2.462 |
| Information | -0.860 | 0.403 | 4.556 | .033† | 0.423 | 0.192 | 0.932 |
| *Note*. Model *R*^2^ = 0.023 (Nagelkerke), Model *χ*^2^ (4) = 14.935, *p* = .005. Control condition is the reference category. Symbol † indicates the initially significant results that stopped being significant after the false discovery rate (FDR) correction was applied. | | | | | | | |

Table S10

*Multiple Linear Regression for the Influence of the Intervention Conditions on Keeping Distance*

|  | | | | | | **95% CI for *b*** | |
| --- | --- | --- | --- | --- | --- | --- | --- |
| **Condition** | ***b*** | **Std. Error** | ***β*** | ***t*** | ***p*** | **Lower** | **Upper** |
| (Constant) | 6.497 | 0.052 |  | 124.311 | <.001 | 6.395 | 6.600 |
| Letter | -0.006 | 0.078 | -0.002 | -0.074 | .941 | -0.158 | 0.146 |
| Meaningful Activity | -0.068 | 0.076 | -0.028 | -0.900 | .368 | -0.217 | 0.080 |
| Economy | -0.063 | 0.077 | -0.026 | -0.826 | .409 | -0.214 | 0.087 |
| Information | 0.075 | 0.076 | 0.031 | 0.990 | .322 | -0.073 | 0.223 |
| *Note*. Model *R*^2^ < 0.001. Control condition is the reference category. | | | | | | | |

Table S11

*Multiple Linear Regression for the Influence of the Intervention Conditions on Relative Hand Washing*

|  | | | | | | **95% CI for *b*** | |
| --- | --- | --- | --- | --- | --- | --- | --- |
| **Condition** | ***b*** | **Std. Error** | ***β*** | ***t*** | ***p*** | **Lower** | **Upper** |
| (Constant) | 5.789 | 0.061 |  | 94.654 | <.001 | 5.669 | 5.909 |
| Letter | -0.051 | 0.090 | -0.014 | -0.564 | .573 | -0.226 | 0.125 |
| Meaningful Activity | -0.090 | 0.088 | -0.025 | -1.029 | .304 | -0.262 | 0.082 |
| Economy | -0.114 | 0.087 | -0.032 | -1.309 | .191 | -0.284 | 0.057 |
| Information | 0.031 | 0.087 | 0.009 | 0.359 | .720 | -0.139 | 0.201 |
| *Note*. Model *R*^2^ < 0.001. Control condition is the reference category. | | | | | | | |

Table S12

*Multiple Linear Regression for the Influence of the Intervention Conditions on Disinfect*

|  | | | | | | **95% CI for *b*** | |
| --- | --- | --- | --- | --- | --- | --- | --- |
| **Condition** | ***b*** | **Std. Error** | ***β*** | ***t*** | ***p*** | **Lower** | **Upper** |
| (Constant) | 4.408 | 0.109 |  | 40.299 | <.001 | 4.193 | 4.622 |
| Letter | 0.078 | 0.161 | 0.014 | 0.487 | .626 | -0.237 | 0.394 |
| Meaningful Activity | 0.238 | 0.158 | 0.044 | 1.501 | .134 | -0.073 | 0.548 |
| Economy | 0.189 | 0.157 | 0.036 | 1.206 | .228 | -0.118 | 0.496 |
| Information | 0.179 | 0.158 | 0.033 | 1.133 | .258 | -0.131 | 0.488 |
| *Note*. Model *R*^2^ = 0.002. Control condition is the reference category. | | | | | | | |

Table S13

*Multiple Linear Regression for the Influence of the Intervention Conditions on Hand Washing Times*

|  | | | | | | **95% CI for *b*** | |
| --- | --- | --- | --- | --- | --- | --- | --- |
| **Condition** | ***b*** | **Std. Error** | ***β*** | ***t*** | ***p*** | **Lower** | **Upper** |
| (Constant) | 8.958 | 0.201 |  | 44.634 | <.001 | 8.565 | 9.352 |
| Letter | -0.234 | 0.294 | -0.019 | -0.796 | .426 | -0.811 | 0.343 |
| Meaningful Activity | -0.221 | 0.287 | -0.019 | -0.770 | .442 | -0.784 | 0.342 |
| Economy | 0.046 | 0.285 | 0.004 | 0.160 | .873 | -0.514 | 0.605 |
| Information | 0.244 | 0.284 | 0.021 | 0.856 | .392 | -0.314 | 0.802 |
| *Note*. Model *R*^2^ = 0.001. Control condition is the reference category. | | | | | | | |

# **Moderated Effects: The Influence of the Intervention Conditions on the Dependent Variables as Moderated by Distancing History**

Below we present the regression analyses in which we tested the interaction effects between the four intervention conditions (Letter, Meaningful Activity, Economy, and Information) versus control and distancing history for each of the 11 dependent variables measured in this study. We also present the results of the analyses probing the patterns of significant interactions using the Johnson-Neyman technique (Johnson and Fay, 1950) implemented via the *interactions* package in R (Long, 2019). For informative purposes, these analyses are presented even for the interaction effects that were initially significant but stopped being significant after the false discovery rate (FDR) correction (Benjamini and Hochberg, 1995) was applied.

*General Distancing*

Table S14

*Multiple Linear Regression for the Influence of the Interactions Between the Intervention Conditions and Distancing History on General Distancing*

|  | | | | | | **95% CI for *b*** | |
| --- | --- | --- | --- | --- | --- | --- | --- |
| **Condition** | ***b*** | **Std. Error** | ***β*** | ***t*** | ***p*** | **Lower** | **Upper** |
| (Constant) | 4.367 | 0.065 |  | 66.977 | <.001 | 4.239 | 4.495 |
| Letter | 0.078 | 0.097 | 0.050 | .801 | .423 | -0.113 | 0.269 |
| Meaningful Activity | 0.113 | 0.089 | 0.075 | 1.276 | .202 | -0.061 | 0.287 |
| Economy | 0.155 | 0.093 | 0.103 | 1.675 | .094 | -0.026 | 0.336 |
| Information | 0.259 | 0.089 | 0.174 | 2.925 | .003 | 0.086 | 0.433 |
| Distancing History | 0.016 | 0.003 | 0.251 | 5.584 | <.001 | 0.010 | 0.022 |
| Int. 1 | -0.005 | 0.004 | -0.074 | -1.168 | .243 | -0.013 | 0.003 |
| Int. 2 | -0.006 | 0.004 | -0.092 | -1.512 | .131 | -0.013 | 0.002 |
| Int. 3 | -0.008 | 0.004 | -0.125 | -1.967 | .049† | -0.016 | 0.000 |
| Int. 4 | -0.012 | 0.004 | -0.193 | -3.056 | .002 | -0.019 | -0.004 |
| *Note*. Model *R*^2^ = 0.027. Control condition is the reference category. Symbol † indicates the initially significant interaction effects that stopped being significant after the false discovery rate (FDR) correction was applied. Int. 1 = Interaction between *Letter* and *Distancing History*; Int. 2 = Interaction between *Meaningful Activity* and *Distancing History*; Int. 3 = Interaction between *Economy* and *Distancing History*; Int. 4 = Interaction between *Information* and *Distancing History*. | | | | | | | |

Output of the Johnson-Neyman Analysis probing the pattern of the Interaction Between the *Economy* Condition and *Distancing History*

**JOHNSON-NEYMAN INTERVAL**

The influence of the Economy (vs. Control) condition on General Distancing is not significant (p > .05) for any levels of Distancing History.

Interval calculated using false discovery rate adjusted t = 4.06261097

The figure below corresponds to the visual depiction of the results regarding the pattern of the interaction obtained via the Johnson-Neyman analysis.


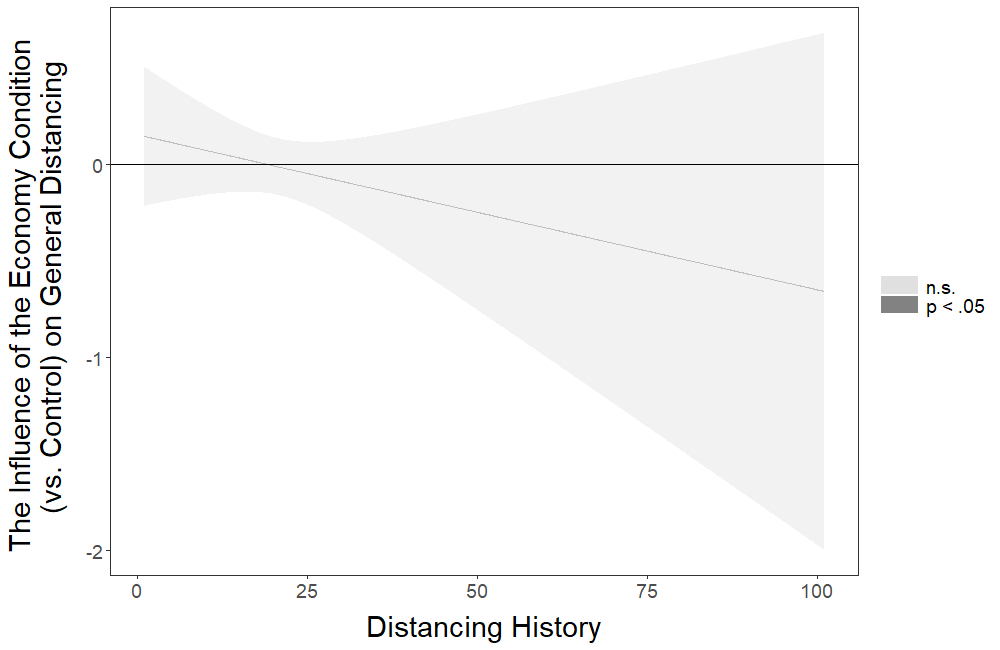


*Figure 1S.* The influence of the economy (vs. control) condition on general distancing at different levels of distancing history. Mean value of distancing history is 21.134.

Output of the Johnson-Neyman Analysis probing the pattern of the Interaction Between the *Information* Condition and *Distancing History*

**JOHNSON-NEYMAN INTERVAL**

When Distancing History is below 14.47230248, the Information (vs. Control) condition has a positive influence on General Distancing (p < .05). When Distancing History is above 31.60822813, the Information (vs. Control) condition has a negative influence on General Distancing (p < .05).

*Note: The range of observed values of Distancing History is* [0.00000000, 101.00000000]

Interval calculated using false discovery rate adjusted t = 2.03891556

The figure below corresponds to the visual depiction of the results regarding the pattern of the interaction obtained via the Johnson-Neyman analysis.


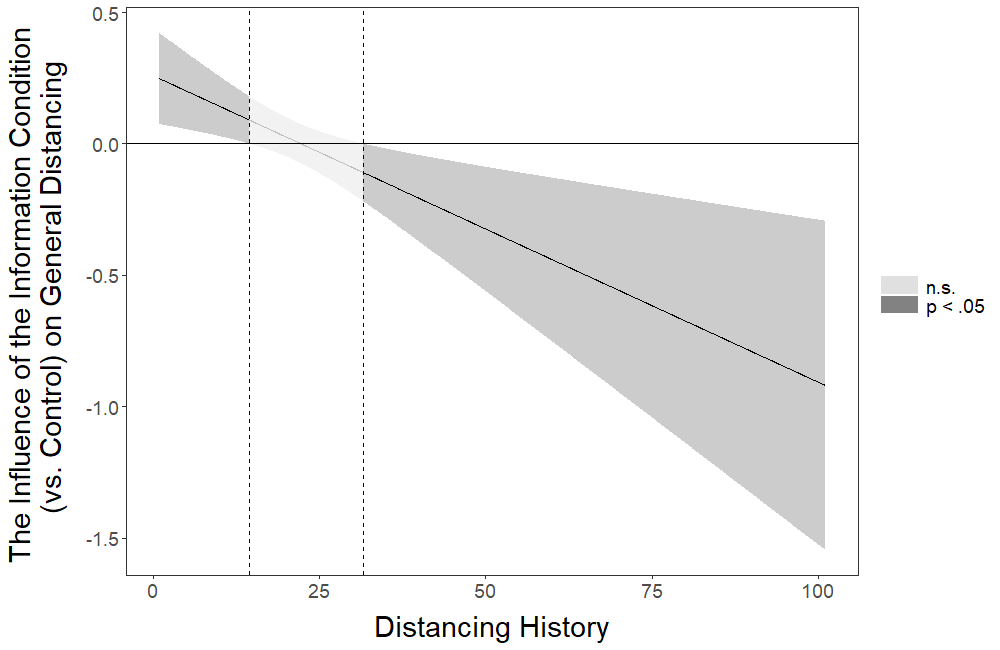


*Figure 2S.* The influence of the information (vs. control) condition on general distancing at different levels of distancing history. Mean value of distancing history is 21.134.

*Going Out Times*

Table S15

*Multiple Linear Regression for the Influence of the Interactions Between the Intervention Conditions and Distancing History on Going Out Times*

|  | | | | | | **95% CI for *b*** | |
| --- | --- | --- | --- | --- | --- | --- | --- |
| **Condition** | ***b*** | **Std. Error** | ***β*** | ***t*** | ***p*** | **Lower** | **Upper** |
| (Constant) | 0.793 | 0.087 |  | 9.151 | <.001 | 0.623 | 0.963 |
| Letter | -0.243 | 0.129 | -0.117 | -1.881 | .060 | -0.497 | 0.010 |
| Meaningful Activity | -0.203 | 0.118 | -0.102 | -1.725 | .085 | -0.434 | 0.028 |
| Economy | -0.236 | 0.123 | -0.119 | -1.917 | .055 | -0.477 | 0.005 |
| Information | -0.479 | 0.118 | -0.243 | -4.065 | <.001 | -0.710 | -0.248 |
| Distancing History | -0.018 | 0.004 | -0.206 | -4.560 | <.001 | -0.025 | -0.010 |
| Int. 1 | 0.008 | 0.006 | 0.094 | 1.482 | .139 | -0.003 | 0.020 |
| Int. 2 | 0.008 | 0.005 | 0.091 | 1.488 | .137 | -0.002 | 0.018 |
| Int. 3 | 0.008 | 0.005 | 0.100 | 1.564 | .118 | -0.002 | 0.019 |
| Int. 4 | 0.021 | 0.005 | 0.262 | 4.126 | <.001 | 0.011 | 0.031 |
| *Note*. Model *R*^2^ = 0.016. Control condition is the reference category. Int. 1 = Interaction between *Letter* and *Distancing History*; Int. 2 = Interaction between *Meaningful Activity* and *Distancing History*; Int. 3 = Interaction between *Economy* and *Distancing History*; Int. 4 = Interaction between *Information* and *Distancing History*. | | | | | | | |

Output of the Johnson-Neyman Analysis probing the pattern of the Interaction Between the *Information* Condition and *Distancing History*

**JOHNSON-NEYMAN INTERVAL**

When Distancing History is below 18.01617226, the Information (vs. Control) condition has a negative influence on Going Out Times (p < .05). When Distancing History is above 28.78473444, the Information (vs. Control) condition has a positive influence on Going Out Times (p < .05).

*Note: The range of observed values of Distancing History is* [0.00000000, 101.00000000]

Interval calculated using false discovery rate adjusted t = 2.00837891

The figure below corresponds to the visual depiction of the results regarding the pattern of the interaction obtained via the Johnson-Neyman analysis.


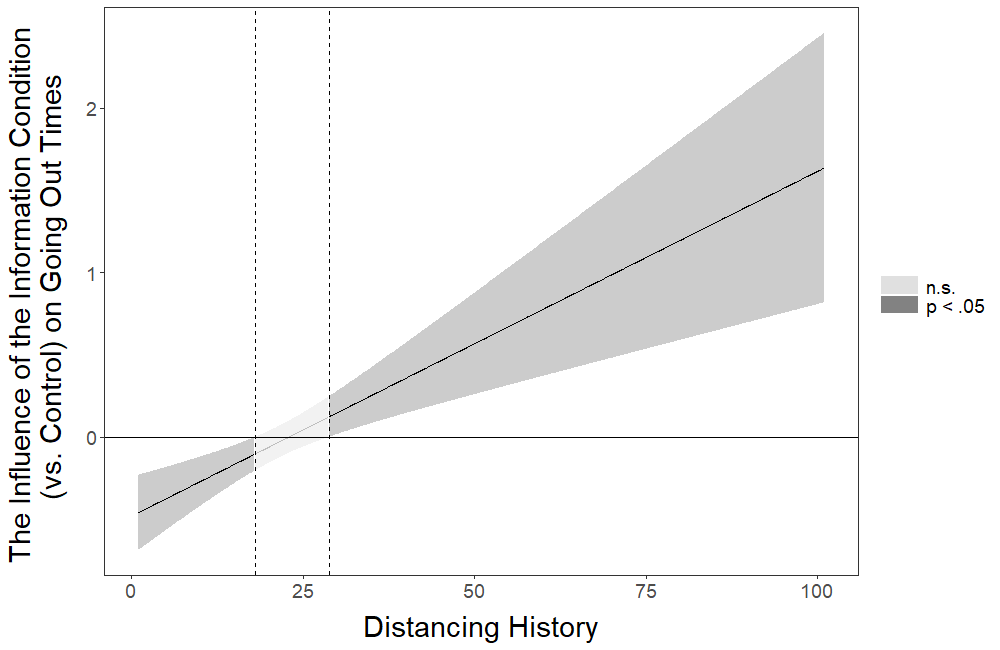


*Figure 3S.* The influence of the information (vs. control) condition on going out times at different levels of distancing history. Mean value of distancing history is 21.134.

*Going Out Hours*

Table S16

*Multiple Linear Regression for the Influence of the Interactions Between the Intervention Conditions and Distancing History on Going Out Hours*

|  | | | | | | **95% CI for *b*** | |
| --- | --- | --- | --- | --- | --- | --- | --- |
| **Condition** | ***b*** | **Std. Error** | ***β*** | ***t*** | ***p*** | **Lower** | **Upper** |
| (Constant) | 0.896 | 0.107 |  | 8.366 | <.001 | 0.686 | 1.106 |
| Letter | -0.447 | 0.160 | -0.175 | -2.794 | .005 | -0.760 | -0.133 |
| Meaningful Activity | -0.290 | 0.146 | -0.117 | -1.986 | .047 | -0.575 | -0.004 |
| Economy | -0.241 | 0.152 | -0.099 | -1.584 | .113 | -0.539 | 0.057 |
| Information | -0.518 | 0.146 | -0.213 | -3.553 | <.001 | -0.804 | -0.232 |
| Distancing History | -0.019 | 0.005 | -0.183 | -4.037 | <.001 | -0.029 | -0.010 |
| Int. 1 | 0.016 | 0.007 | 0.141 | 2.204 | .028† | 0.002 | 0.029 |
| Int. 2 | 0.011 | 0.006 | 0.108 | 1.748 | .081 | -0.001 | 0.024 |
| Int. 3 | 0.009 | 0.007 | 0.081 | 1.274 | .203 | -0.005 | 0.022 |
| Int. 4 | 0.020 | 0.006 | 0.206 | 3.236 | .001 | 0.008 | 0.033 |
| *Note*. Model *R*^2^ = 0.011. Control condition is the reference category. Symbol † indicates the initially significant interaction effects that stopped being significant after the false discovery rate (FDR) correction was applied. Int. 1 = Interaction between *Letter* and *Distancing History*; Int. 2 = Interaction between *Meaningful Activity* and *Distancing History*; Int. 3 = Interaction between *Economy* and *Distancing History*; Int. 4 = Interaction between *Information* and *Distancing History*. | | | | | | | |

Output of the Johnson-Neyman Analysis probing the pattern of the Interaction Between the *Letter* Condition and *Distancing History*

**JOHNSON-NEYMAN INTERVAL**

When Distancing History is below 17.68586447, the Letter (vs. Control) condition has a negative influence on Going Out Hours (p < .05). When Distancing History is above this value, the influence of the Letter (vs. Control) condition on Going Out Hours is not significant (p > .05).

*Note: The range of observed values of Distancing History is* [0.00000000, 101.00000000]

Interval calculated using false discovery rate adjusted t = 2.62203245

The figure below corresponds to the visual depiction of the results regarding the pattern of the interaction obtained via the Johnson-Neyman analysis.


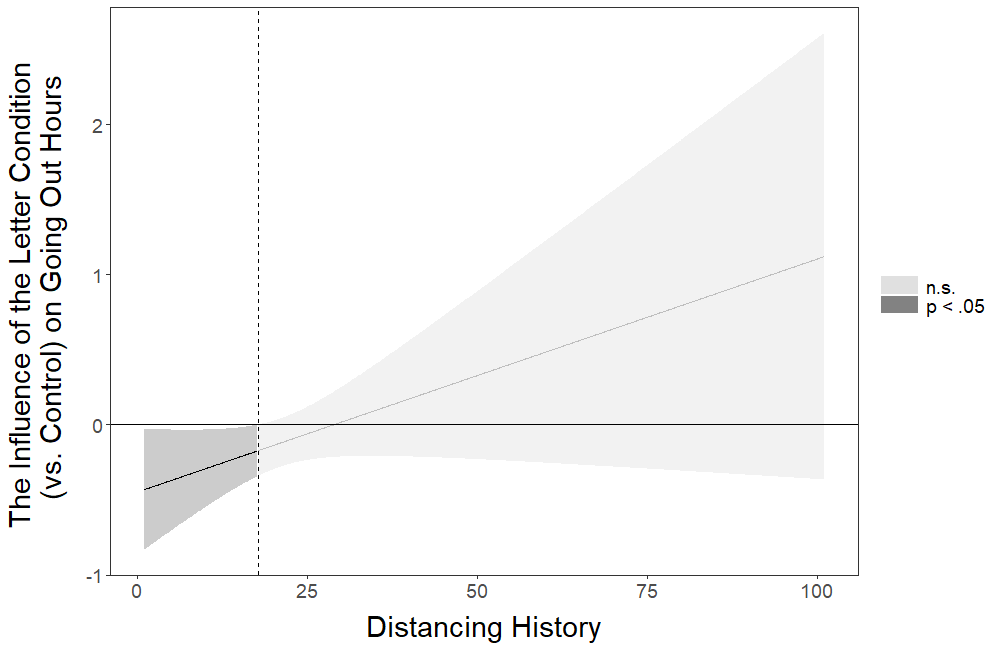


*Figure 4S.* The influence of the letter (vs. control) condition on going out hours at different levels of distancing history. Mean value of distancing history is 21.134.

Output of the Johnson-Neyman Analysis probing the pattern of the Interaction Between the *Information* Condition and *Distancing History*

**JOHNSON-NEYMAN INTERVAL**

When Distancing History is below 19.41481621, the Information (vs. Control) condition has a negative influence on Going Out Hours (p < .05). When Distancing History is above 37.26880824, the Information (vs. Control) condition has a positive influence on Going Out Hours (p < .05).

*Note: The range of observed values of Distancing History is* [0.00000000, 101.00000000]

Interval calculated using false discovery rate adjusted t = 2.04242823

The figure below corresponds to the visual depiction of the results regarding the pattern of the interaction obtained via the Johnson-Neyman analysis.


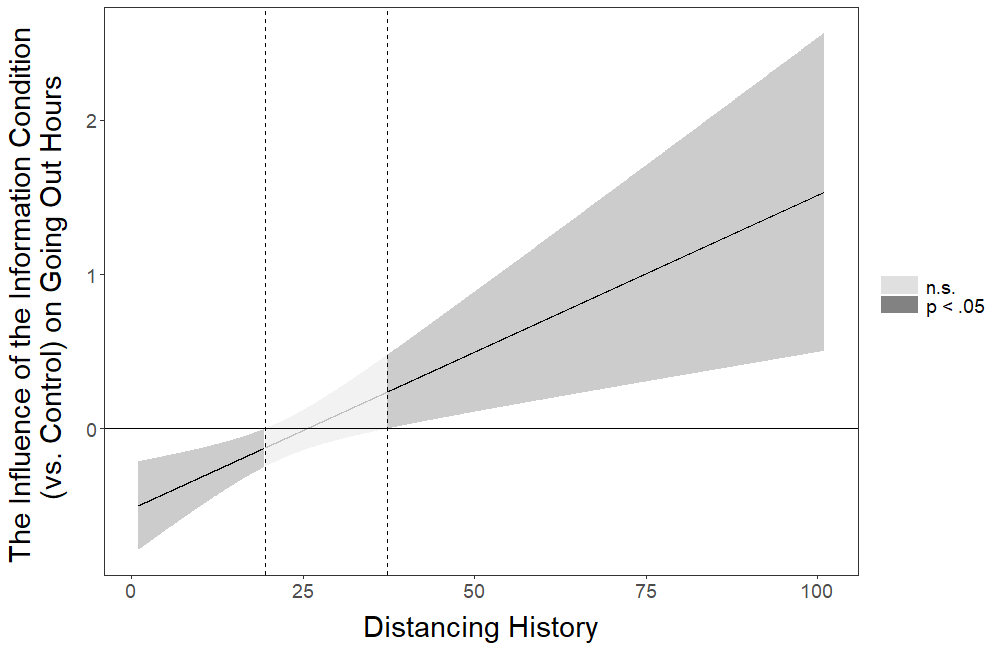


*Figure 5S.* The influence of the information (vs. control) condition on going out hours at different levels of distancing history. Mean value of distancing history is 21.134.

*Physical Fitness Times*

Table S17

*Multiple Linear Regression for the Influence of the Interactions Between the Intervention Conditions and Distancing History on Physical Fitness Times*

|  | | | | | | **95% CI for *b*** | |
| --- | --- | --- | --- | --- | --- | --- | --- |
| **Condition** | ***b*** | **Std. Error** | ***β*** | ***t*** | ***p*** | **Lower** | **Upper** |
| (Constant) | 0.584 | 0.079 |  | 7.352 | <.001 | 0.428 | 0.740 |
| Letter | 0.097 | 0.118 | 0.051 | 0.819 | .413 | -0.135 | 0.329 |
| Meaningful Activity | -0.128 | 0.108 | -0.070 | -1.182 | .237 | -0.340 | 0.084 |
| Economy | 0.006 | 0.113 | 0.003 | 0.054 | .957 | -0.215 | 0.227 |
| Information | -0.102 | 0.108 | -0.057 | -0.944 | .345 | -0.314 | 0.110 |
| Distancing History | -0.004 | 0.004 | -0.057 | -1.247 | .213 | -0.011 | 0.003 |
| Int. 1 | -0.006 | 0.005 | -0.079 | -1.235 | .217 | -0.017 | 0.004 |
| Int. 2 | 0.004 | 0.005 | 0.048 | 0.777 | .437 | -0.006 | 0.013 |
| Int. 3 | -0.004 | 0.005 | -0.053 | -0.824 | .410 | -0.014 | 0.006 |
| Int. 4 | 0.004 | 0.005 | 0.054 | 0.852 | .394 | -0.005 | 0.013 |
| *Note*. Model *R*^2^ = 0.007. Control condition is the reference category. Int. 1 = Interaction between *Letter* and *Distancing History*; Int. 2 = Interaction between *Meaningful Activity* and *Distancing History*; Int. 3 = Interaction between *Economy* and *Distancing History*; Int. 4 = Interaction between *Information* and *Distancing History*. | | | | | | | |

*Note*: Johnson-Neyman analysis was not conducted for Physical Fitness Times because no significant interaction effects were identified.

*Physical Fitness Hours*

Table S18

*Multiple Linear Regression for the Influence of the Interactions Between the Intervention Conditions and Distancing History on Physical Fitness Hours*

|  | | | | | | **95% CI for *b*** | |
| --- | --- | --- | --- | --- | --- | --- | --- |
| **Condition** | ***b*** | **Std. Error** | ***β*** | ***t*** | ***p*** | **Lower** | **Upper** |
| (Constant) | 0.717 | 0.076 |  | 9.395 | <.001 | 0.567 | 0.866 |
| Letter | 0.001 | 0.114 | 0.001 | 0.009 | .993 | -0.222 | 0.224 |
| Meaningful Activity | -0.173 | 0.104 | -0.099 | -1.670 | .095 | -0.377 | 0.030 |
| Economy | -0.217 | 0.108 | -0.125 | -2.001 | .046 | -0.429 | -0.004 |
| Information | -0.269 | 0.104 | -0.155 | -2.589 | .010 | -0.472 | -0.065 |
| Distancing History | -0.010 | 0.003 | -0.138 | -3.035 | .002 | -0.017 | -0.004 |
| Int. 1 | -0.002 | 0.005 | -0.027 | -0.423 | .673 | -0.012 | 0.008 |
| Int. 2 | 0.006 | 0.005 | 0.086 | 1.392 | .164 | -0.003 | 0.015 |
| Int. 3 | 0.008 | 0.005 | 0.101 | 1.585 | .113 | -0.002 | 0.017 |
| Int. 4 | 0.010 | 0.004 | 0.140 | 2.192 | .028† | 0.001 | 0.019 |
| *Note*. Model *R*^2^ = 0.010. Control condition is the reference category. Symbol † indicates the initially significant interaction effects that stopped being significant after the false discovery rate (FDR) correction was applied. Int. 1 = Interaction between *Letter* and *Distancing History*; Int. 2 = Interaction between *Meaningful Activity* and *Distancing History*; Int. 3 = Interaction between *Economy* and *Distancing History*; Int. 4 = Interaction between *Information* and *Distancing History*. | | | | | | | |

Output of the Johnson-Neyman Analysis probing the pattern of the Interaction Between the *Information* Condition and *Distancing History*

**JOHNSON-NEYMAN INTERVAL**

The influence of the Information (vs. Control) condition on Physical Fitness Hours is not significant (p > .05) for any levels of Distancing History.

Interval calculated using false discovery rate adjusted t = 4.06261097

The figure below corresponds to the visual depiction of the results regarding the pattern of the interaction obtained via the Johnson-Neyman analysis.


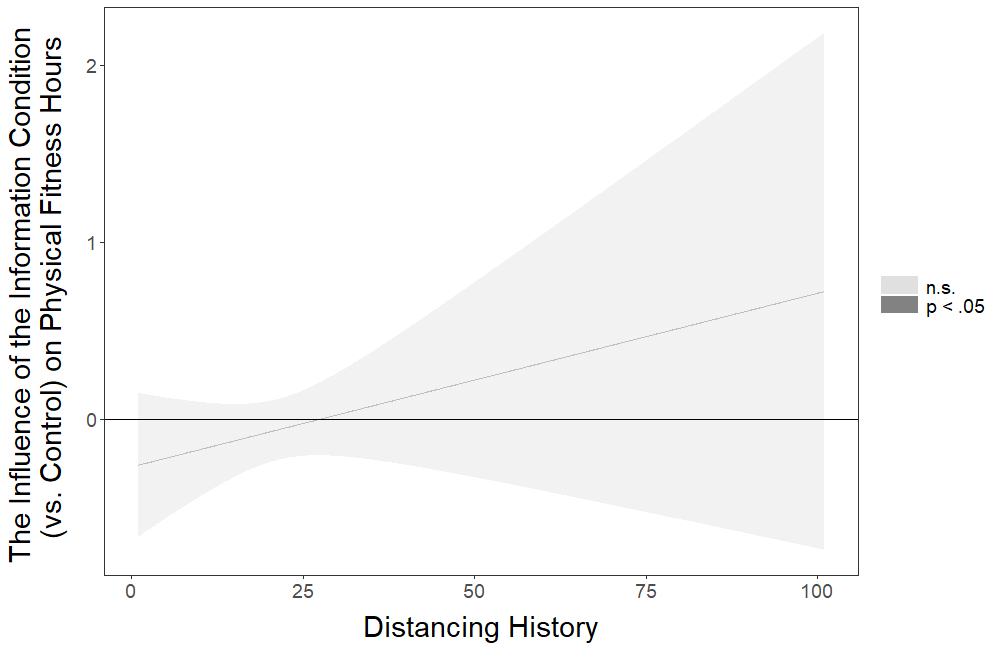


*Figure 6S.* The influence of the information (vs. control) condition on physical fitness hours at different levels of distancing history. Mean value of distancing history is 21.134.

*Out Family Friends*

Table S19

*Multiple Logistic Regression for the Influence of the Interactions Between the Intervention Conditions and Distancing History on Out Family Friends*

|  | | | | | | **95% CI for Odds Ratio** | |
| --- | --- | --- | --- | --- | --- | --- | --- |
| **Condition** | ***b*** | **Std. Error** | **Wald** | ***p*** | **Odds Ratio** | **Lower** | **Upper** |
| (Constant) | -2.107 | 0.626 | 11.315 | .001 | 0.122 |  |  |
| Letter | -0.377 | 1.017 | 0.137 | .711 | 0.686 | 0.094 | 5.035 |
| Meaningful Activity | 0.561 | 0.908 | 0.381 | .537 | 1.752 | 0.296 | 10.388 |
| Economy | -0.603 | 0.875 | 0.474 | .491 | 0.547 | 0.098 | 3.042 |
| Information | -0.409 | 1.025 | 0.159 | .690 | 0.664 | 0.089 | 4.957 |
| Distancing History | -0.056 | 0.033 | 2.914 | .088 | 0.946 | 0.887 | 1.008 |
| Int. 1 | 0.012 | 0.052 | 0.051 | .822 | 1.012 | 0.914 | 1.119 |
| Int. 2 | -0.042 | 0.050 | 0.709 | .400 | 0.959 | 0.870 | 1.057 |
| Int. 3 | 0.036 | 0.044 | 0.680 | .409 | 1.037 | 0.952 | 1.129 |
| Int. 4 | 0.007 | 0.052 | 0.020 | .888 | 1.007 | 0.910 | 1.115 |
| *Note*. Model *R*^2^ = 0.023 (Nagelkerke), Model *χ*^2^ (9) = 16.043, *p* = .066. Control condition is the reference category. Int. 1 = Interaction between *Letter* and *Distancing History*; Int. 2 = Interaction between *Meaningful Activity* and *Distancing History*; Int. 3 = Interaction between *Economy* and *Distancing History*; Int. 4 = Interaction between *Information* and *Distancing History*. | | | | | | | |

*Note*: Johnson-Neyman analysis was not conducted for Out Family Friends because no significant interaction effects were identified.

*Social Gatherings*

Table S20

*Multiple Logistic Regression for the Influence of the Interactions Between the Intervention Conditions and Distancing History on Social Gatherings*

|  | | | | | | **95% CI for Odds Ratio** | |
| --- | --- | --- | --- | --- | --- | --- | --- |
| **Condition** | ***b*** | **Std. Error** | **Wald** | ***p*** | **Odds Ratio** | **Lower** | **Upper** |
| (Constant) | -3.403 | 0.533 | 40.782 | <.001 | 0.033 |  |  |
| Letter | 0.124 | 0.948 | 0.017 | .896 | 1.132 | 0.177 | 7.254 |
| Meaningful Activity | -0.235 | 1.002 | 0.055 | .814 | 0.790 | 0.111 | 5.629 |
| Economy | 0.682 | 0.750 | 0.827 | .363 | 1.978 | 0.455 | 8.610 |
| Information | -0.563 | 0.997 | 0.320 | .572 | 0.569 | 0.081 | 4.015 |
| Distancing History | 0.008 | 0.023 | 0.138 | .711 | 1.008 | 0.965 | 1.054 |
| Int. 1 | -0.019 | 0.042 | 0.201 | .654 | 0.981 | 0.903 | 1.066 |
| Int. 2 | -0.023 | 0.045 | 0.256 | .613 | 0.977 | 0.894 | 1.068 |
| Int. 3 | -0.017 | 0.033 | 0.272 | .602 | 0.983 | 0.921 | 1.049 |
| Int. 4 | -0.014 | 0.043 | 0.106 | .745 | 0.986 | 0.907 | 1.072 |
| *Note*. Model *R*^2^ = 0.024 (Nagelkerke), Model *χ*^2^ (9) = 15.429, *p* = .080. Control condition is the reference category. Int. 1 = Interaction between *Letter* and *Distancing History*; Int. 2 = Interaction between *Meaningful Activity* and *Distancing History*; Int. 3 = Interaction between *Economy* and *Distancing History*; Int. 4 = Interaction between *Information* and *Distancing History*. | | | | | | | |

*Note*: Johnson-Neyman analysis was not conducted for Social Gatherings because no significant interaction effects were identified.

*Keeping Distance*

Table S21

*Multiple Linear Regression for the Influence of the Interactions Between the Intervention Conditions and Distancing History on Keeping Distance*

|  | | | | | | **95% CI for *b*** | |
| --- | --- | --- | --- | --- | --- | --- | --- |
| **Condition** | ***b*** | **Std. Error** | ***β*** | ***t*** | ***p*** | **Lower** | **Upper** |
| (Constant) | 6.021 | 0.134 |  | 45.019 | <.001 | 5.759 | 6.284 |
| Letter | 0.054 | 0.239 | 0.022 | 0.227 | .821 | -0.415 | 0.523 |
| Meaningful Activity | 0.125 | 0.178 | 0.051 | 0.703 | .482 | -0.224 | 0.475 |
| Economy | -0.059 | 0.211 | -0.024 | -0.281 | .779 | -0.473 | 0.354 |
| Information | 0.415 | 0.179 | 0.171 | 2.315 | .021 | 0.063 | 0.766 |
| Distancing History | 0.024 | 0.006 | 0.219 | 3.857 | <.001 | 0.012 | 0.036 |
| Int. 1 | -0.003 | 0.011 | -0.027 | -0.286 | .775 | -0.025 | 0.019 |
| Int. 2 | -0.010 | 0.008 | -0.097 | -1.244 | .214 | -0.026 | 0.006 |
| Int. 3 | 0.000 | 0.010 | -0.004 | -0.045 | .964 | -0.020 | 0.019 |
| Int. 4 | -0.018 | 0.008 | -0.180 | -2.229 | .026† | -0.033 | -0.002 |
| *Note*. Model *R*^2^ = 0.026. Control condition is the reference category. Symbol † indicates the initially significant interaction effects that stopped being significant after the false discovery rate (FDR) correction was applied. Int. 1 = Interaction between *Letter* and *Distancing History*; Int. 2 = Interaction between *Meaningful Activity* and *Distancing History*; Int. 3 = Interaction between *Economy* and *Distancing History*; Int. 4 = Interaction between *Information* and *Distancing History*. | | | | | | | |

Output of the Johnson-Neyman Analysis probing the pattern of the Interaction Between the *Information* Condition and *Distancing History*

**JOHNSON-NEYMAN INTERVAL**

The influence of the Information (vs. Control) condition on Keeping Distance is not significant (p > .05) for any levels of Distancing History.

Interval calculated using false discovery rate adjusted t = 4.06720170

The figure below corresponds to the visual depiction of the results regarding the pattern of the interaction obtained via the Johnson-Neyman analysis.


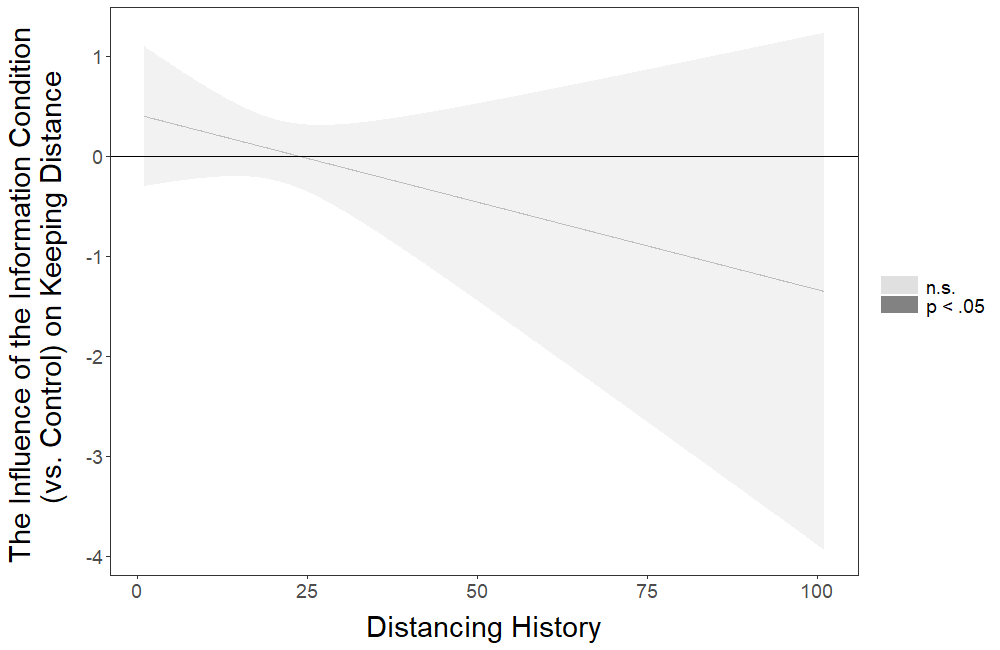


*Figure 7S.* The influence of the information (vs. control) condition on keeping distance at different levels of distancing history. Mean value of distancing history is 21.134.

*Relative Hand Washing*

Table S22

*Multiple Linear Regression for the Influence of the Interactions Between the Intervention Conditions and Distancing History on Relative Hand Washing*

|  | | | | | | **95% CI for *b*** | |
| --- | --- | --- | --- | --- | --- | --- | --- |
| **Condition** | ***b*** | **Std. Error** | ***β*** | ***t*** | ***p*** | **Lower** | **Upper** |
| (Constant) | 5.536 | 0.156 |  | 35.396 | <.001 | 5.230 | 5.843 |
| Letter | 0.023 | 0.233 | 0.006 | 0.098 | .922 | -0.434 | 0.480 |
| Meaningful Activity | -0.145 | 0.213 | -0.040 | -0.683 | .495 | -0.563 | 0.272 |
| Economy | 0.096 | 0.222 | 0.027 | 0.433 | .665 | -0.339 | 0.531 |
| Information | 0.082 | 0.213 | 0.023 | 0.387 | .699 | -0.335 | 0.500 |
| Distancing History | 0.012 | 0.007 | 0.080 | 1.756 | .079 | -0.001 | 0.026 |
| Int. 1 | -0.004 | 0.010 | -0.023 | -0.359 | .719 | -0.024 | 0.016 |
| Int. 2 | 0.002 | 0.009 | 0.016 | 0.252 | .801 | -0.016 | 0.021 |
| Int. 3 | -0.010 | 0.010 | -0.066 | -1.035 | .301 | -0.029 | 0.009 |
| Int. 4 | -0.003 | 0.009 | -0.021 | -0.326 | .745 | -0.021 | 0.015 |
| *Note*. Model *R*^2^ = 0.006. Control condition is the reference category. Int. 1 = Interaction between *Letter* and *Distancing History*; Int. 2 = Interaction between *Meaningful Activity* and *Distancing History*; Int. 3 = Interaction between *Economy* and *Distancing History*; Int. 4 = Interaction between *Information* and *Distancing History*. | | | | | | | |

*Note*: Johnson-Neyman analysis was not conducted for Relative Hand Washing because no significant interaction effects were identified.

*Disinfect*

Table S23

*Multiple Linear Regression for the Influence of the Interactions Between the Intervention Conditions and Distancing History on Disinfect*

|  | | | | | | **95% CI for *b*** | |
| --- | --- | --- | --- | --- | --- | --- | --- |
| **Condition** | ***b*** | **Std. Error** | ***β*** | ***t*** | ***p*** | **Lower** | **Upper** |
| (Constant) | 3.681 | 0.287 |  | 12.842 | <.001 | 3.119 | 4.244 |
| Letter | -0.504 | 0.460 | -0.092 | -1.097 | .273 | -1.406 | 0.398 |
| Meaningful Activity | 0.494 | 0.385 | 0.092 | 1.283 | .200 | -0.261 | 1.249 |
| Economy | -0.113 | 0.412 | -0.021 | -0.274 | .784 | -0.921 | 0.695 |
| Information | -0.138 | 0.385 | -0.026 | -0.359 | .720 | -0.894 | 0.617 |
| Distancing History | 0.036 | 0.013 | 0.151 | 2.735 | .006 | 0.010 | 0.061 |
| Int. 1 | 0.027 | 0.021 | 0.110 | 1.294 | .196 | -0.014 | 0.068 |
| Int. 2 | -0.014 | 0.017 | -0.061 | -0.799 | .425 | -0.047 | 0.020 |
| Int. 3 | 0.014 | 0.019 | 0.059 | 0.741 | .459 | -0.023 | 0.050 |
| Int. 4 | 0.012 | 0.017 | 0.057 | 0.726 | .468 | -0.021 | 0.045 |
| *Note*. Model *R*^2^ = 0.035. Control condition is the reference category. Int. 1 = Interaction between *Letter* and *Distancing History*; Int. 2 = Interaction between *Meaningful Activity* and *Distancing History*; Int. 3 = Interaction between *Economy* and *Distancing History*; Int. 4 = Interaction between *Information* and *Distancing History*. | | | | | | | |

*Note*: Johnson-Neyman analysis was not conducted for Disinfect because no significant interaction effects were identified.

*Hand Washing Times*

Table S24

*Multiple Linear Regression for the Influence of the Interactions Between the Intervention Conditions and Distancing Hand Washing Times*

|  | | | | | | **95% CI for *b*** | |
| --- | --- | --- | --- | --- | --- | --- | --- |
| **Condition** | ***b*** | **Std. Error** | ***β*** | ***t*** | ***p*** | **Lower** | **Upper** |
| (Constant) | 7.635 | 0.512 |  | 14.918 | <.001 | 6.631 | 8.639 |
| Letter | -0.113 | 0.763 | -0.009 | -0.148 | .882 | -1.610 | 1.384 |
| Meaningful Activity | 0.853 | 0.696 | 0.072 | 1.225 | .221 | -0.512 | 2.218 |
| Economy | 0.732 | 0.726 | 0.063 | 1.008 | .314 | -0.692 | 2.156 |
| Information | 0.147 | 0.696 | 0.013 | 0.211 | .833 | -1.219 | 1.512 |
| Distancing History | 0.064 | 0.023 | 0.127 | 2.809 | .005 | 0.019 | 0.108 |
| Int. 1 | -0.007 | 0.034 | -0.013 | -0.203 | .839 | -0.073 | 0.059 |
| Int. 2 | -0.053 | 0.030 | -0.106 | -1.727 | .084 | -0.112 | 0.007 |
| Int. 3 | -0.033 | 0.032 | -0.067 | -1.043 | .297 | -0.096 | 0.029 |
| Int. 4 | 0.001 | 0.030 | 0.003 | 0.045 | .964 | -0.057 | 0.060 |
| *Note*. Model *R*^2^ = 0.011. Control condition is the reference category. Int. 1 = Interaction between *Letter* and *Distancing History*; Int. 2 = Interaction between *Meaningful Activity* and *Distancing History*; Int. 3 = Interaction between *Economy* and *Distancing History*; Int. 4 = Interaction between *Information* and *Distancing History*. | | | | | | | |

*Note*: Johnson-Neyman analysis was not conducted for Hand Washing Times because no significant interaction effects were identified.

# **Moderated Effects: The Influence of the Intervention Conditions on the Dependent Variables as Moderated by Living Situation**

Below we present the regression analyses in which we tested the interaction effects between the four intervention conditions (Letter, Meaningful Activity, Economy, and Information) versus control and living situation for each of the 11 dependent variables measured in this study. We also present the results of the analyses probing the patterns of significant interactions using the Johnson-Neyman technique (Johnson and Fay, 1950) implemented via the *interactions* package in R (Long, 2019). For informative purposes, these analyses are presented even for the interaction effects that were initially significant but stopped being significant after the false discovery rate (FDR) correction (Benjamini and Hochberg, 1995) was applied.

*General Distancing*

Table S25

*Multiple Linear Regression for the Influence of the Interactions Between the Intervention Conditions and Living Situation on General Distancing*

|  | | | | | | **95% CI for *b*** | |
| --- | --- | --- | --- | --- | --- | --- | --- |
| **Condition** | ***b*** | **Std. Error** | ***β*** | ***t*** | ***p*** | **Lower** | **Upper** |
| (Constant) | 4.286 | 0.111 |  | 38.564 | <.001 | 4.068 | 4.504 |
| Letter | 0.388 | 0.151 | 0.247 | 2.571 | .010 | 0.092 | 0.684 |
| Meaningful Activity | 0.234 | 0.152 | 0.155 | 1.536 | .125 | -0.065 | 0.532 |
| Economy | 0.457 | 0.151 | 0.305 | 3.031 | .002 | 0.161 | 0.753 |
| Information | 0.283 | 0.153 | 0.190 | 1.848 | .065 | -0.017 | 0.583 |
| Living Situation | 0.072 | 0.019 | 0.176 | 3.844 | <.001 | 0.035 | 0.109 |
| Int. 1 | -0.071 | 0.026 | -0.269 | -2.781 | .005† | -0.122 | -0.021 |
| Int. 2 | -0.041 | 0.026 | -0.159 | -1.580 | .114 | -0.092 | 0.010 |
| Int. 3 | -0.081 | 0.026 | -0.320 | -3.171 | .002† | -0.132 | -0.031 |
| Int. 4 | -0.045 | 0.026 | -0.180 | -1.748 | .081 | -0.096 | 0.006 |
| *Note*. Model *R*^2^ = 0.008. Control condition is the reference category. Symbol † indicates the initially significant interaction effects that stopped being significant after the false discovery rate (FDR) correction was applied. Int. 1 = Interaction between *Letter* and *Living Situation*; Int. 2 = Interaction between *Meaningful Activity* and *Living Situation*; Int. 3 = Interaction between *Economy* and *Living Situation*; Int. 4 = Interaction between *Information* and *Living Situation*. | | | | | | | |

Output of the Johnson-Neyman Analysis probing the pattern of the Interaction Between the *Letter* Condition and *Living Situation*

**JOHNSON-NEYMAN INTERVAL**

The influence of the Letter (vs. Control) condition on General Distancing is not significant (p > .05) for any levels of Living Situation.

Interval calculated using false discovery rate adjusted t = 4.06260582

The figure below corresponds to the visual depiction of the results regarding the pattern of the interaction obtained via the Johnson-Neyman analysis.


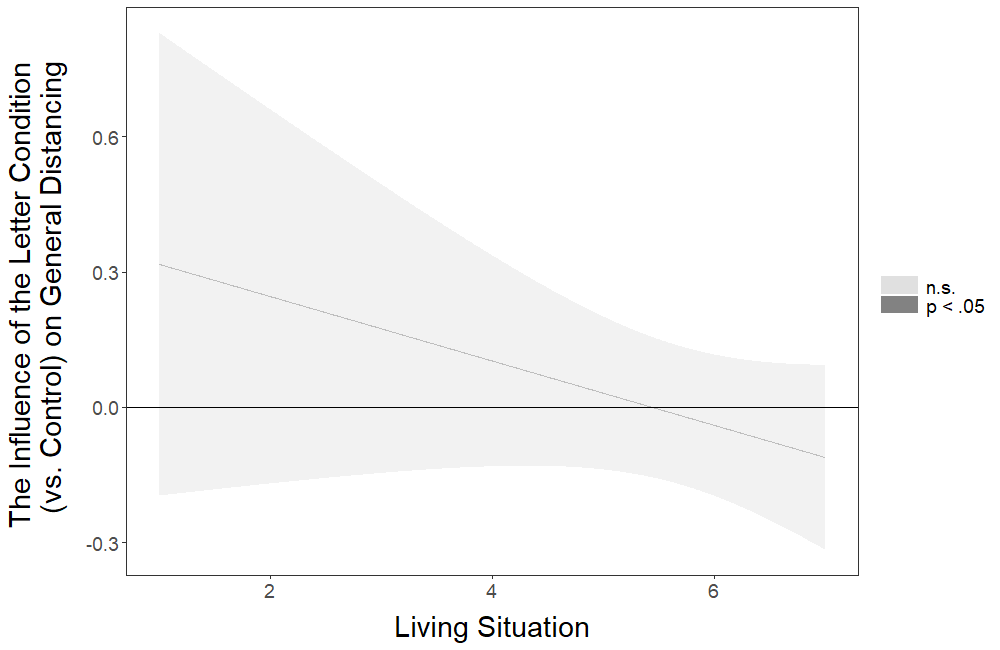


*Figure 8S.* The influence of the letter (vs. control) condition on general distancing at different levels of living situation. Mean value of living situation is 5.671.

Output of the Johnson-Neyman Analysis probing the pattern of the Interaction Between the *Economy* Condition and *Living Situation*

**JOHNSON-NEYMAN INTERVAL**

When Living Situation is below 4.13576465, the Economy (vs. Control) condition has a positive influence on General Distancing (p < .05). When Living Situation is above 6.92940372, the Economy (vs. Control) condition has a negative influence on General Distancing (p < .05).

*Note: The range of observed values of Living Situation is* [1.00000000, 7.00000000]

Interval calculated using false discovery rate adjusted t = 2.21678275

The figure below corresponds to the visual depiction of the results regarding the pattern of the interaction obtained via the Johnson-Neyman analysis.


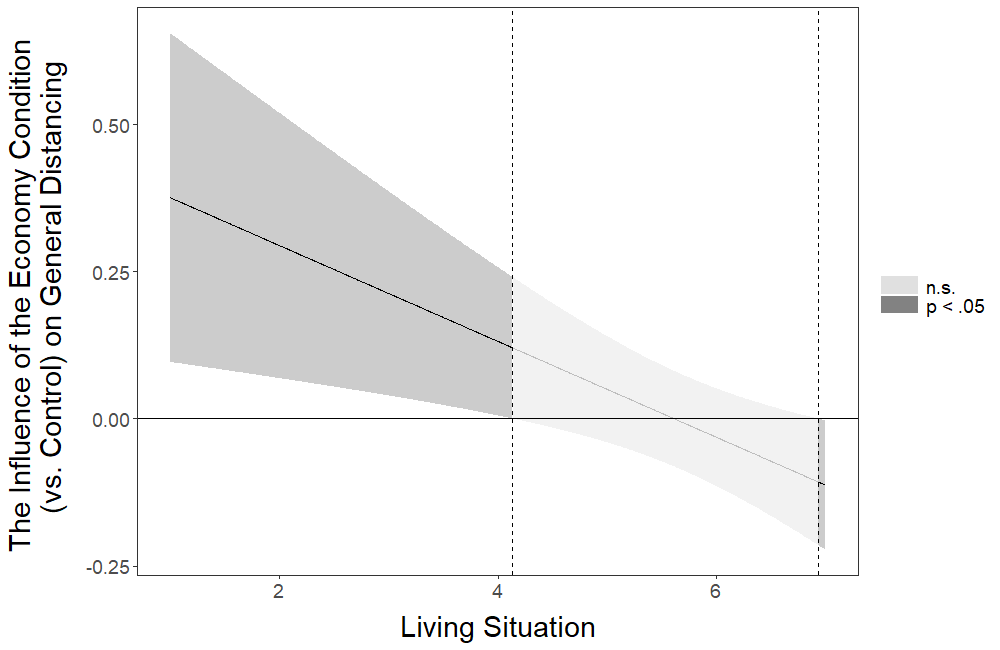


*Figure 9S.* The influence of the economy (vs. control) condition on general distancing at different levels of living situation. Mean value of living situation is 5.671.

*Going Out Times*

Table S26

*Multiple Linear Regression for the Influence of the Interactions Between the Intervention Conditions and Living Situation on Going Out Times*

|  | | | | | | **95% CI for *b*** | |
| --- | --- | --- | --- | --- | --- | --- | --- |
| **Condition** | ***b*** | **Std. Error** | ***β*** | ***t*** | ***p*** | **Lower** | **Upper** |
| (Constant) | 0.932 | 0.147 |  | 6.341 | <.001 | 0.644 | 1.221 |
| Letter | -0.543 | 0.200 | -0.262 | -2.718 | .007 | -0.934 | -0.151 |
| Meaningful Activity | -0.514 | 0.201 | -0.257 | -2.552 | .011 | -0.909 | -0.119 |
| Economy | -0.381 | 0.200 | -0.192 | -1.909 | .056 | -0.772 | 0.010 |
| Information | -0.490 | 0.203 | -0.248 | -2.418 | .016 | -0.887 | -0.093 |
| Living Situation | -0.088 | 0.025 | -0.161 | -3.518 | <.001 | -0.136 | -0.039 |
| Int. 1 | 0.082 | 0.034 | 0.233 | 2.415 | .016† | 0.015 | 0.148 |
| Int. 2 | 0.082 | 0.034 | 0.238 | 2.372 | .018† | 0.014 | 0.149 |
| Int. 3 | 0.055 | 0.034 | 0.163 | 1.619 | .106 | -0.012 | 0.122 |
| Int. 4 | 0.078 | 0.034 | 0.234 | 2.268 | .023† | 0.011 | 0.145 |
| *Note*. Model *R*^2^ = 0.006. Control condition is the reference category. Symbol † indicates the initially significant interaction effects that stopped being significant after the false discovery rate (FDR) correction was applied. Int. 1 = Interaction between *Letter* and *Living Situation*; Int. 2 = Interaction between *Meaningful Activity* and *Living Situation*; Int. 3 = Interaction between *Economy* and *Living Situation*; Int. 4 = Interaction between *Information* and *Living Situation*. | | | | | | | |

Output of the Johnson-Neyman Analysis probing the pattern of the Interaction Between the *Letter* Condition and *Living Situation*

**JOHNSON-NEYMAN INTERVAL**

When Living Situation is below 5.29736328, the Letter (vs. Control) condition has a negative influence on Going Out Times (p < .05). When Living Situation is above 5.29736328, the Letter (vs. Control) condition does not influence Going Out Times (p > .05).

*Note: The range of observed values of Living Situation is* [1.00000000, 7.00000000]

Interval calculated using false discovery rate adjusted t = 2.10011120

The figure below corresponds to the visual depiction of the results regarding the pattern of the interaction obtained via the Johnson-Neyman analysis.


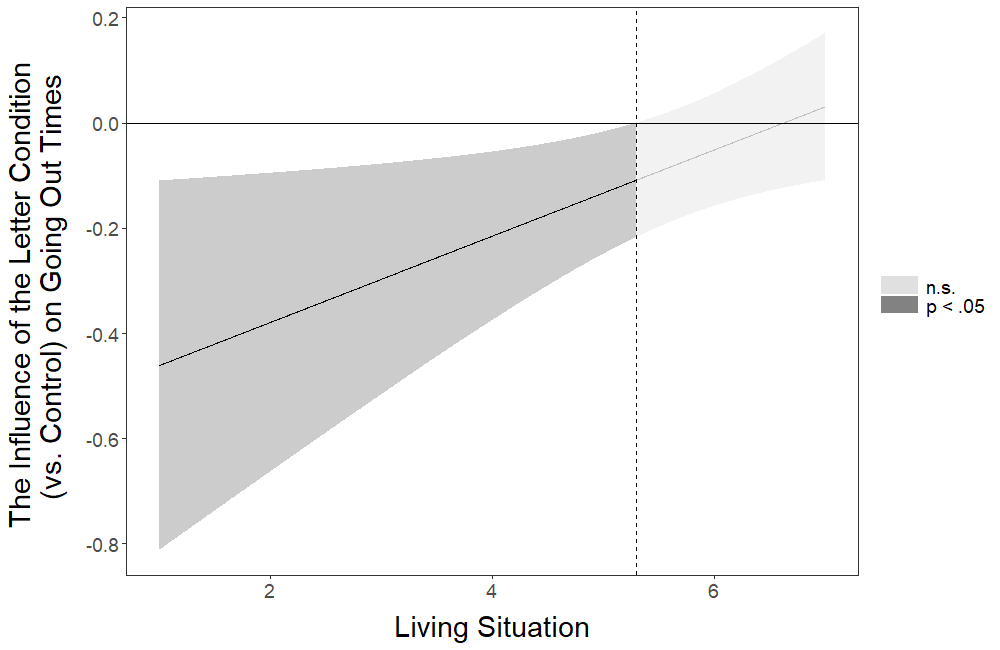


*Figure 10S.* The influence of the letter (vs. control) condition on going out times at different levels of living situation. Mean value of living situation is 5.671.

Output of the Johnson-Neyman Analysis probing the pattern of the Interaction Between the *Meaningful Activity* Condition and *Living Situation*

**JOHNSON-NEYMAN INTERVAL**

When Living Situation is below 4.77169897, the Meaningful Activity (vs. Control) condition has a negative influence on Going Out Times (p < .05). When Living Situation is above 4.77169897, the Meaningful Activity (vs. Control) condition does not influence Going Out Times (p > .05).

*Note: The range of observed values of Living Situation is* [1.00000000, 7.00000000]

Interval calculated using false discovery rate adjusted t = 2.15288401

The figure below corresponds to the visual depiction of the results regarding the pattern of the interaction obtained via the Johnson-Neyman analysis.


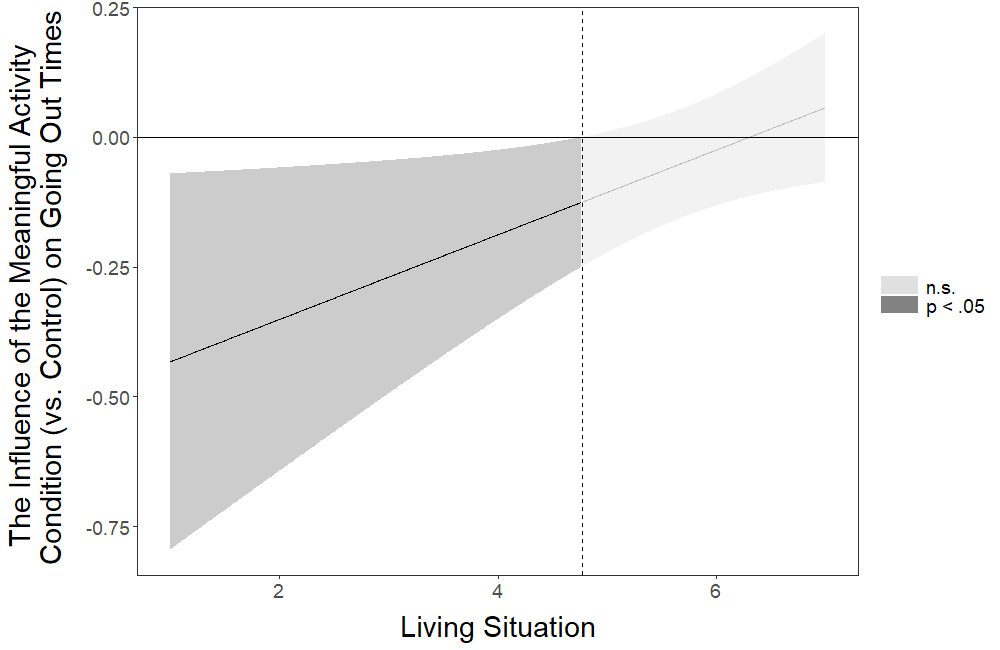


*Figure 11S.* The influence of the meaningful activity (vs. control) condition on going out times at different levels of living situation. Mean value of living situation is 5.671.

Output of the Johnson-Neyman Analysis probing the pattern of the Interaction Between the *Information* Condition and *Living Situation*

**JOHNSON-NEYMAN INTERVAL**

When Living Situation is below 4.47818152, the Information (vs. Control) condition has a negative influence on Going Out Times (p < .05). When Living Situation is above 4.47818152, the Information (vs. Control) condition does not influence Going Out Times (p > .05).

*Note: The range of observed values of Living Situation is* [1.00000000, 7.00000000]

Interval calculated using false discovery rate adjusted t = 2.18508020

The figure below corresponds to the visual depiction of the results regarding the pattern of the interaction obtained via the Johnson-Neyman analysis.


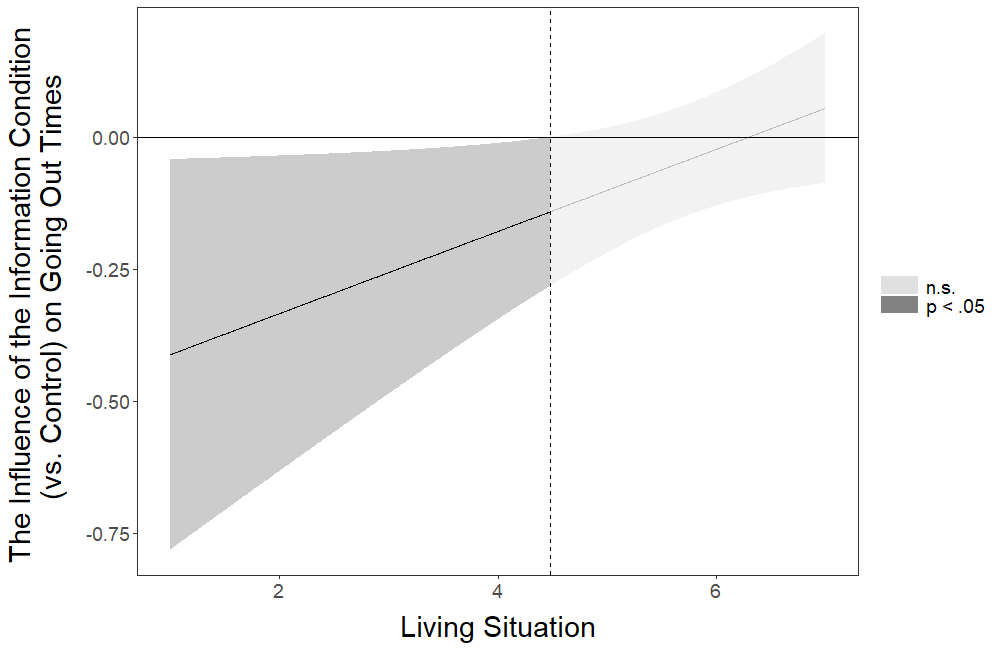


*Figure 12S.* The influence of the information (vs. control) condition on going out times at different levels of living situation. Mean value of living situation is 5.671.

*Going Out Hours*

Table S27

*Multiple Linear Regression for the Influence of the Interactions Between the Intervention Conditions and Living Situation on Going Out Hours*

|  | | | | | | **95% CI for *b*** | |
| --- | --- | --- | --- | --- | --- | --- | --- |
| **Condition** | ***b*** | **Std. Error** | ***β*** | ***t*** | ***p*** | **Lower** | **Upper** |
| (Constant) | 0.830 | 0.182 |  | 4.568 | <.001 | 0.474 | 1.186 |
| Letter | -0.517 | 0.247 | -0.202 | -2.097 | .036 | -1.001 | -0.034 |
| Meaningful Activity | -0.468 | 0.249 | -0.190 | -1.879 | .060 | -0.956 | 0.020 |
| Economy | -0.476 | 0.247 | -0.195 | -1.931 | .054 | -0.960 | 0.007 |
| Information | -0.480 | 0.250 | -0.197 | -1.917 | .055 | -0.970 | 0.011 |
| Living Situation | -0.058 | 0.031 | -0.086 | -1.877 | .061 | -0.118 | 0.003 |
| Int. 1 | 0.068 | 0.042 | 0.158 | 1.630 | .103 | -0.014 | 0.150 |
| Int. 2 | 0.072 | 0.043 | 0.170 | 1.684 | .092 | -0.012 | 0.155 |
| Int. 3 | 0.072 | 0.042 | 0.173 | 1.707 | .088 | -0.011 | 0.154 |
| Int. 4 | 0.067 | 0.042 | 0.163 | 1.574 | .116 | -0.016 | 0.150 |
| *Note*. Model *R*^2^ = 0.003. Control condition is the reference category. Int. 1 = Interaction between *Letter* and *Living Situation*; Int. 2 = Interaction between *Meaningful Activity* and *Living Situation*; Int. 3 = Interaction between *Economy* and *Living Situation*; Int. 4 = Interaction between *Information* and *Living Situation*. | | | | | | | |

*Note*: Johnson-Neyman analysis was not conducted for Going Out Hours because no significant interaction effects were identified.

*Physical Fitness Times*

Table S28

*Multiple Linear Regression for the Influence of the Interactions Between the Intervention Conditions and Living Situation on Physical Fitness Times*

|  | | | | | | **95% CI for *b*** | |
| --- | --- | --- | --- | --- | --- | --- | --- |
| **Condition** | ***b*** | **Std. Error** | ***β*** | ***t*** | ***p*** | **Lower** | **Upper** |
| (Constant) | 0.818 | 0.135 |  | 6.063 | <.001 | 0.553 | 1.082 |
| Letter | -0.419 | 0.183 | -0.220 | -2.287 | .022 | -0.778 | -0.060 |
| Meaningful Activity | -0.281 | 0.185 | -0.154 | -1.523 | .128 | -0.644 | 0.081 |
| Economy | -0.466 | 0.183 | -0.257 | -2.545 | .011 | -0.825 | -0.107 |
| Information | -0.483 | 0.186 | -0.267 | -2.603 | .009 | -0.847 | -0.119 |
| Living Situation | -0.057 | 0.023 | -0.113 | -2.476 | .013 | -0.101 | -0.012 |
| Int. 1 | 0.066 | 0.031 | 0.205 | 2.120 | .034† | 0.005 | 0.127 |
| Int. 2 | 0.041 | 0.032 | 0.130 | 1.291 | .197 | -0.021 | 0.103 |
| Int. 3 | 0.067 | 0.031 | 0.218 | 2.156 | .031† | 0.006 | 0.128 |
| Int. 4 | 0.081 | 0.031 | 0.266 | 2.570 | .010† | 0.019 | 0.143 |
| *Note*. Model *R*^2^ = 0.005. Control condition is the reference category. Symbol † indicates the initially significant interaction effects that stopped being significant after the false discovery rate (FDR) correction was applied. Int. 1 = Interaction between *Letter* and *Living Situation*; Int. 2 = Interaction between *Meaningful Activity* and *Living Situation*; Int. 3 = Interaction between *Economy* and *Living Situation*; Int. 4 = Interaction between *Information* and *Living Situation*. | | | | | | | |

Output of the Johnson-Neyman Analysis probing the pattern of the Interaction Between the *Letter* Condition and *Living Situation*

**JOHNSON-NEYMAN INTERVAL**

The influence of the Letter (vs. Control) condition on Physical Fitness Times is not significant (p > .05) for any levels of Living Situation.

Interval calculated using false discovery rate adjusted t = 4.06260582

The figure below corresponds to the visual depiction of the results regarding the pattern of the interaction obtained via the Johnson-Neyman analysis.


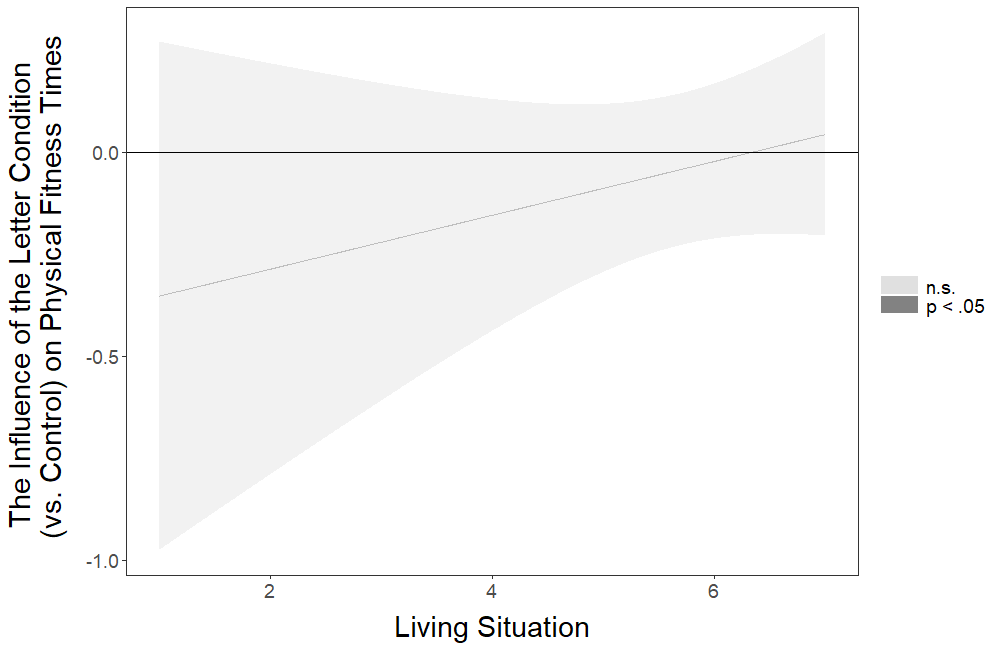


*Figure 13S.* The influence of the letter (vs. control) condition on physical fitness times at different levels of living situation. Mean value of living situation is 5.671.

Output of the Johnson-Neyman Analysis probing the pattern of the Interaction Between the *Economy* Condition and *Living Situation*

**JOHNSON-NEYMAN INTERVAL**

When Living Situation is below 5.55505044, the Economy (vs. Control) condition has a negative influence on Physical Fitness Times (p < .05). When Living Situation is above 5.55505044, the Economy (vs. Control) condition does not influence Physical Fitness Times (p > .05).

*Note: The range of observed values of Living Situation is* [1.00000000, 7.00000000]

Interval calculated using false discovery rate adjusted t = 2.07631295

The figure below corresponds to the visual depiction of the results regarding the pattern of the interaction obtained via the Johnson-Neyman analysis.


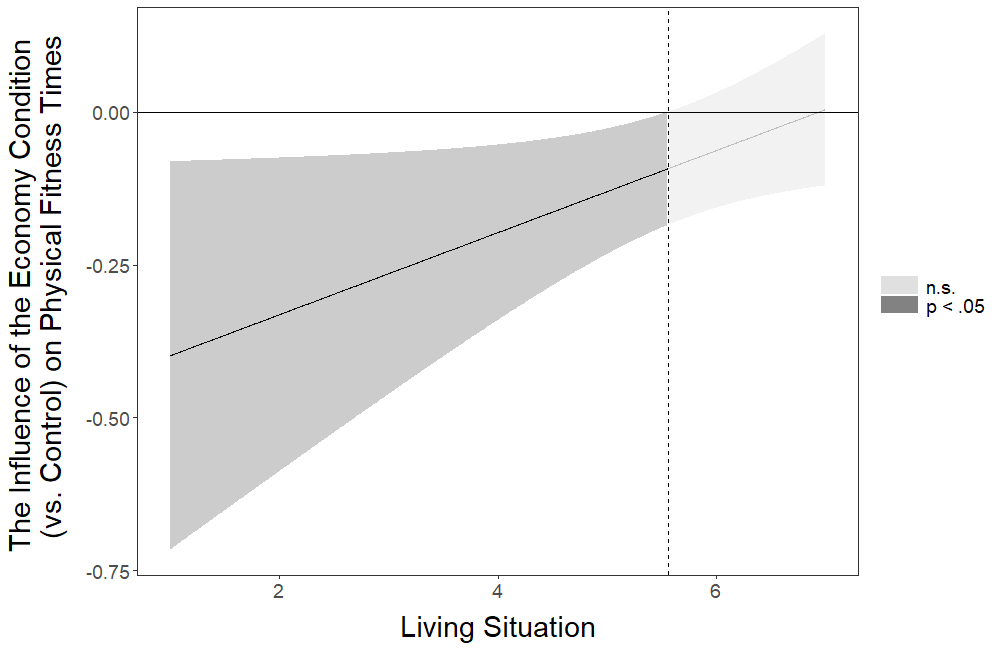


*Figure 14S.* The influence of the letter (vs. control) condition on physical fitness times at different levels of living situation. Mean value of living situation is 5.671.

Output of the Johnson-Neyman Analysis probing the pattern of the Interaction Between the *Information* Condition and *Living Situation*

**JOHNSON-NEYMAN INTERVAL**

When Living Situation is below 4.13253812, the Information (vs. Control) condition has a negative influence on Physical Fitness Times (p < .05). When Living Situation is above 4.13253812, the Information (vs. Control) condition does not influence Physical Fitness Times (p > .05).

*Note: The range of observed values of Living Situation is* [1.00000000, 7.00000000]

Interval calculated using false discovery rate adjusted t = 2.22562504

The figure below corresponds to the visual depiction of the results regarding the pattern of the interaction obtained via the Johnson-Neyman analysis.


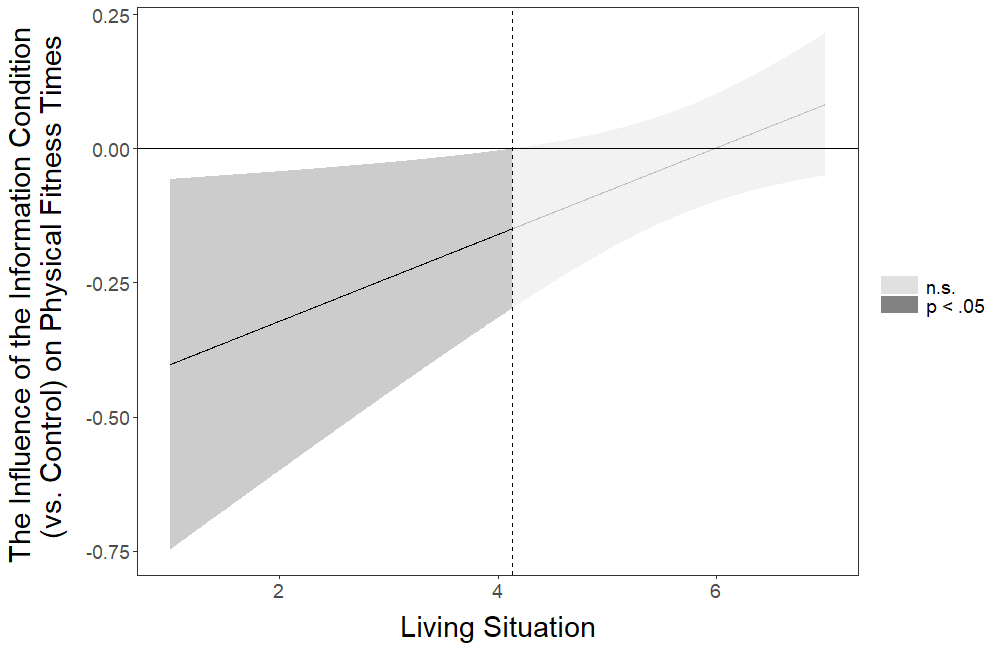


*Figure 15S.* The influence of the information (vs. control) condition on physical fitness times at different levels of living situation. Mean value of living situation is 5.671.

*Physical Fitness Hours*

Table S29

*Multiple Linear Regression for the Influence of the Interactions Between the Intervention Conditions and Living Situation on Physical Fitness Hours*

|  | | | | | | **95% CI for *b*** | |
| --- | --- | --- | --- | --- | --- | --- | --- |
| **Condition** | ***b*** | **Std. Error** | ***β*** | ***t*** | ***p*** | **Lower** | **Upper** |
| (Constant) | 0.652 | 0.130 |  | 5.007 | <.001 | 0.397 | 0.907 |
| Letter | -0.252 | 0.177 | -0.138 | -1.427 | .154 | -0.599 | 0.094 |
| Meaningful Activity | -0.017 | 0.178 | -0.010 | -0.094 | .925 | -0.367 | 0.333 |
| Economy | -0.349 | 0.177 | -0.199 | -1.976 | .048 | -0.696 | -0.003 |
| Information | -0.375 | 0.179 | -0.215 | -2.090 | .037 | -0.726 | -0.023 |
| Living Situation | -0.026 | 0.022 | -0.054 | -1.171 | .242 | -0.069 | 0.017 |
| Int. 1 | 0.036 | 0.030 | 0.115 | 1.192 | .233 | -0.023 | 0.095 |
| Int. 2 | -0.004 | 0.030 | -0.013 | -0.128 | .899 | -0.064 | 0.056 |
| Int. 3 | 0.051 | 0.030 | 0.171 | 1.687 | .092 | -0.008 | 0.110 |
| Int. 4 | 0.054 | 0.030 | 0.184 | 1.779 | .075 | -0.006 | 0.114 |
| *Note*. Model *R*^2^ = 0.004. Control condition is the reference category. Int. 1 = Interaction between *Letter* and *Living Situation*; Int. 2 = Interaction between *Meaningful Activity* and *Living Situation*; Int. 3 = Interaction between *Economy* and *Living Situation*; Int. 4 = Interaction between *Information* and *Living Situation*. | | | | | | | |

*Note*: Johnson-Neyman analysis was not conducted for *Physical Fitness Hours* because no significant interaction effects were identified.

*Out Family Friends*

Table S30

*Multiple Logistic Regression for the Influence of the Interactions Between the Intervention Conditions and Living Situation on Out Family Friends*

|  | | | | | | **95% CI for Odds Ratio** | |
| --- | --- | --- | --- | --- | --- | --- | --- |
| **Condition** | ***b*** | **Std. Error** | **Wald** | ***p*** | **Odds Ratio** | **Lower** | **Upper** |
| (Constant) | -1.614 | 0.694 | 5.412 | .020 | 0.199 |  |  |
| Letter | -3.047 | 1.460 | 4.356 | .037 | 0.047 | 0.003 | 0.831 |
| Meaningful Activity | -1.191 | 1.096 | 1.182 | .277 | 0.304 | 0.035 | 2.602 |
| Economy | -1.515 | 1.089 | 1.933 | .164 | 0.220 | 0.026 | 1.860 |
| Information | -0.132 | 0.993 | 0.018 | .895 | 0.877 | 0.125 | 6.143 |
| Living Situation | -0.286 | 0.128 | 5.005 | .025 | 0.751 | 0.584 | 0.965 |
| Int. 1 | 0.508 | 0.244 | 4.321 | .038† | 1.662 | 1.029 | 2.682 |
| Int. 2 | 0.190 | 0.198 | 0.919 | .338 | 1.209 | 0.820 | 1.781 |
| Int. 3 | 0.290 | 0.193 | 2.262 | .133 | 1.336 | 0.916 | 1.948 |
| Int. 4 | -0.043 | 0.187 | 0.053 | .817 | 0.958 | 0.664 | 1.382 |
| *Note*. Model *R*^2^ = 0.019 (Nagelkerke), Model *χ*^2^ (9) = 13.062, *p* = .160. Symbol † indicates the initially significant interaction effects that stopped being significant after the false discovery rate (FDR) correction was applied. Control condition is the reference category. Int. 1 = Interaction between *Letter* and *Living Situation*; Int. 2 = Interaction between *Meaningful Activity* and *Living Situation*; Int. 3 = Interaction between *Economy* and *Living Situation*; Int. 4 = Interaction between *Information* and *Living Situation*. | | | | | | | |

Output of the Johnson-Neyman Analysis probing the pattern of the Interaction Between the *Letter* Condition and *Living Situation*

**JOHNSON-NEYMAN INTERVAL**

The influence of the Letter (vs. Control) condition on Out Family Friends is not significant (p > .05) for any levels of Living Situation.

Interval calculated using false discovery rate adjusted t = 4.06260582

The figure below corresponds to the visual depiction of the results regarding the pattern of the interaction obtained via the Johnson-Neyman analysis.


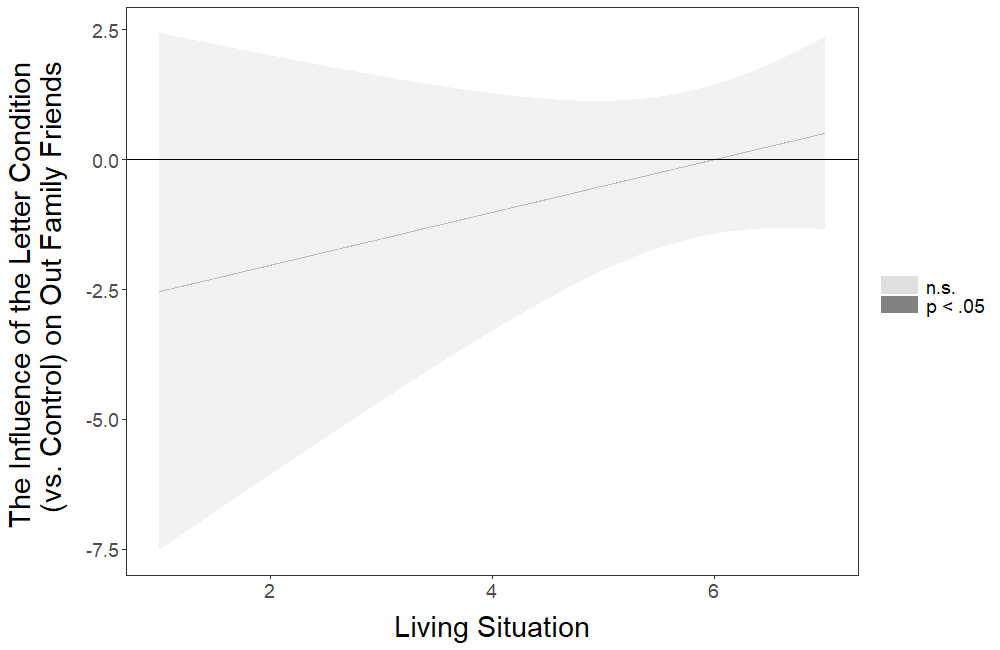


*Figure 16S.* The influence of the letter (vs. control) condition on physical fitness times at different levels of living situation. Mean value of living situation is 5.671.

*Social Gatherings*

Table S31

*Multiple Logistic Regression for the Influence of the Interactions Between the Intervention Conditions and Living Situation on Social Gatherings*

|  | | | | | | **95% CI for Odds Ratio** | |
| --- | --- | --- | --- | --- | --- | --- | --- |
| **Condition** | ***b*** | **Std. Error** | **Wald** | ***p*** | **Odds Ratio** | **Lower** | **Upper** |
| (Constant) | -2.596 | 0.848 | 9.377 | .002 | 0.075 |  |  |
| Letter | -1.471 | 1.433 | 1.054 | .305 | 0.230 | 0.014 | 3.808 |
| Meaningful Activity | -1.903 | 1.660 | 1.314 | .252 | 0.149 | 0.006 | 3.858 |
| Economy | 0.968 | 1.031 | 0.883 | .348 | 2.634 | 0.349 | 19.858 |
| Information | -2.481 | 1.891 | 1.721 | .190 | 0.084 | 0.002 | 3.407 |
| Living Situation | -0.112 | 0.148 | 0.571 | .450 | 0.894 | 0.669 | 1.195 |
| Int. 1 | 0.210 | 0.242 | 0.753 | .385 | 1.234 | 0.768 | 1.983 |
| Int. 2 | 0.210 | 0.282 | 0.554 | .457 | 1.233 | 0.710 | 2.142 |
| Int. 3 | -0.125 | 0.183 | 0.468 | .494 | 0.882 | 0.616 | 1.264 |
| Int. 4 | 0.281 | 0.313 | 0.802 | .370 | 1.324 | 0.716 | 2.447 |
| *Note*. Model *R*^2^ = 0.032 (Nagelkerke), Model *χ*^2^ (9) = 20.654, *p* = .014. Control condition is the reference category. Int. 1 = Interaction between *Letter* and *Living Situation*; Int. 2 = Interaction between *Meaningful Activity* and *Living Situation*; Int. 3 = Interaction between *Economy* and *Living Situation*; Int. 4 = Interaction between *Information* and *Living Situation*. | | | | | | | |

*Note*: Johnson-Neyman analysis was not conducted for *Social Gatherings* because no significant interaction effects were identified.

*Keeping Distance*

Table S32

*Multiple Linear Regression for the Influence of the Interactions Between the Intervention Conditions and Living Situation on Keeping Distance*

|  | | | | | | **95% CI for *b*** | |
| --- | --- | --- | --- | --- | --- | --- | --- |
| **Condition** | ***b*** | **Std. Error** | ***β*** | ***t*** | ***p*** | **Lower** | **Upper** |
| (Constant) | 6.008 | 0.216 |  | 27.804 | <.001 | 5.584 | 6.432 |
| Letter | 0.178 | 0.311 | 0.071 | 0.571 | .568 | -0.432 | 0.788 |
| Meaningful Activity | 0.478 | 0.294 | 0.197 | 1.630 | .103 | -0.097 | 1.054 |
| Economy | 0.251 | 0.315 | 0.101 | 0.794 | .427 | -0.368 | 0.869 |
| Information | -0.385 | 0.311 | -0.159 | -1.236 | .217 | -0.996 | 0.226 |
| Living Situation | 0.086 | 0.037 | 0.129 | 2.331 | .020 | 0.014 | 0.158 |
| Int. 1 | -0.032 | 0.053 | -0.076 | -0.606 | .544 | -0.136 | 0.072 |
| Int. 2 | -0.097 | 0.051 | -0.228 | -1.899 | .058 | -0.196 | 0.003 |
| Int. 3 | -0.056 | 0.054 | -0.134 | -1.039 | .299 | -0.161 | 0.049 |
| Int. 4 | 0.079 | 0.053 | 0.195 | 1.499 | .134 | -0.024 | 0.183 |
| *Note*. Model *R*^2^ = 0.020. Control condition is the reference category. Int. 1 = Interaction between *Letter* and *Living Situation*; Int. 2 = Interaction between *Meaningful Activity* and *Living Situation*; Int. 3 = Interaction between *Economy* and *Living Situation*; Int. 4 = Interaction between *Information* and *Living Situation*. | | | | | | | |

*Note*: Johnson-Neyman analysis was not conducted for *Keeping Distance* because no significant interaction effects were identified.

*Relative Hand Washing*

Table S33

*Multiple Linear Regression for the Influence of the Interactions Between the Intervention Conditions and Living Situation on Relative Hand Washing*

|  | | | | | | **95% CI for *b*** | |
| --- | --- | --- | --- | --- | --- | --- | --- |
| **Condition** | ***b*** | **Std. Error** | ***β*** | ***t*** | ***p*** | **Lower** | **Upper** |
| (Constant) | 5.966 | 0.264 |  | 22.559 | <.001 | 5.447 | 6.484 |
| Letter | -0.294 | 0.359 | -0.079 | -0.820 | .412 | -0.998 | 0.410 |
| Meaningful Activity | -0.142 | 0.362 | -0.040 | -0.392 | .695 | -0.853 | 0.568 |
| Economy | -0.646 | 0.359 | -0.182 | -1.799 | .072 | -1.349 | 0.058 |
| Information | -0.402 | 0.364 | -0.114 | -1.105 | .269 | -1.117 | 0.312 |
| Living Situation | -0.031 | 0.045 | -0.031 | -0.686 | .493 | -0.119 | 0.057 |
| Int. 1 | 0.043 | 0.061 | 0.068 | 0.699 | .485 | -0.077 | 0.162 |
| Int. 2 | 0.009 | 0.062 | 0.014 | 0.138 | .890 | -0.113 | 0.130 |
| Int. 3 | 0.094 | 0.061 | 0.155 | 1.533 | .125 | -0.026 | 0.213 |
| Int. 4 | 0.076 | 0.062 | 0.127 | 1.227 | .220 | -0.045 | 0.197 |
| *Note*. Model *R*^2^ = 0.003. Control condition is the reference category. Int. 1 = Interaction between *Letter* and *Living Situation*; Int. 2 = Interaction between *Meaningful Activity* and *Living Situation*; Int. 3 = Interaction between *Economy* and *Living Situation*; Int. 4 = Interaction between *Information* and *Living Situation*. | | | | | | | |

*Note*: Johnson-Neyman analysis was not conducted for *Relative Hand Washing* because no significant interaction effects were identified.

*Disinfect*

Table S34

*Multiple Linear Regression for the Influence of the Interactions Between the Intervention Conditions and Living Situation on Disinfect*

|  | | | | | | **95% CI for *b*** | |
| --- | --- | --- | --- | --- | --- | --- | --- |
| **Condition** | ***b*** | **Std. Error** | ***β*** | ***t*** | ***p*** | **Lower** | **Upper** |
| (Constant) | 4.065 | 0.489 |  | 8.316 | <.001 | 3.106 | 5.024 |
| Letter | 0.012 | 0.665 | 0.002 | 0.018 | .986 | -1.292 | 1.316 |
| Meaningful Activity | 0.276 | 0.662 | 0.051 | 0.417 | .677 | -1.022 | 1.574 |
| Economy | -0.219 | 0.662 | -0.042 | -0.332 | .740 | -1.517 | 1.078 |
| Information | 0.000 | 0.672 | 0.000 | 0.001 | >.999 | -1.317 | 1.318 |
| Living Situation | 0.060 | 0.083 | 0.040 | 0.720 | .472 | -0.103 | 0.223 |
| Int. 1 | 0.013 | 0.113 | 0.014 | 0.114 | .909 | -0.209 | 0.235 |
| Int. 2 | -0.005 | 0.114 | -0.005 | -0.041 | .967 | -0.228 | 0.218 |
| Int. 3 | 0.073 | 0.113 | 0.081 | 0.648 | .517 | -0.148 | 0.294 |
| Int. 4 | 0.032 | 0.115 | 0.036 | 0.283 | .777 | -0.192 | 0.257 |
| *Note*. Model *R*^2^ = 0.005. Control condition is the reference category. Int. 1 = Interaction between *Letter* and *Living Situation*; Int. 2 = Interaction between *Meaningful Activity* and *Living Situation*; Int. 3 = Interaction between *Economy* and *Living Situation*; Int. 4 = Interaction between *Information* and *Living Situation*. | | | | | | | |

*Note*: Johnson-Neyman analysis was not conducted for *Disinfect* because no significant interaction effects were identified.

*Hand Washing Times*

Table S35

*Multiple Linear Regression for the Influence of the Interactions Between the Intervention Conditions and Living Situation on Hand Washing Times*

|  | | | | | | **95% CI for *b*** | |
| --- | --- | --- | --- | --- | --- | --- | --- |
| **Condition** | ***b*** | **Std. Error** | ***β*** | ***t*** | ***p*** | **Lower** | **Upper** |
| (Constant) | 9.176 | 0.868 |  | 10.568 | <.001 | 7.473 | 10.878 |
| Letter | 0.283 | 1.179 | 0.023 | 0.240 | .810 | -2.028 | 2.594 |
| Meaningful Activity | -0.179 | 1.190 | -0.015 | -0.151 | .880 | -2.512 | 2.153 |
| Economy | 0.242 | 1.178 | 0.021 | 0.205 | .838 | -2.069 | 2.552 |
| Information | -0.166 | 1.196 | -0.014 | -0.139 | .890 | -2.511 | 2.179 |
| Living Situation | -0.038 | 0.147 | -0.012 | -0.257 | .797 | -0.326 | 0.250 |
| Int. 1 | -0.092 | 0.200 | -0.045 | -0.461 | .645 | -0.485 | 0.300 |
| Int. 2 | -0.008 | 0.203 | -0.004 | -0.041 | .967 | -0.407 | 0.390 |
| Int. 3 | -0.035 | 0.201 | -0.018 | -0.177 | .860 | -0.429 | 0.358 |
| Int. 4 | 0.072 | 0.203 | 0.037 | 0.353 | .724 | -0.326 | 0.469 |
| *Note*. Model *R*^2^ = 0.002. Control condition is the reference category. Int. 1 = Interaction between *Letter* and *Living Situation*; Int. 2 = Interaction between *Meaningful Activity* and *Living Situation*; Int. 3 = Interaction between *Economy* and *Living Situation*; Int. 4 = Interaction between *Information* and *Living Situation*. | | | | | | | |

*Note*: Johnson-Neyman analysis was not conducted for *Hand Washing Times* because no significant interaction effects were identified.

**Moderated Effects: The Influence of the Intervention Conditions on the Dependent Variables as Moderated by Economic Reasons**

Below we present the regression analyses in which we tested the interaction effects between the four intervention conditions (Letter, Meaningful Activity, Economy, and Information) versus control and economic reasons for each of the 11 dependent variables measured in this study. We also present the results of the analyses probing the patterns of significant interactions using the Johnson-Neyman technique (Johnson and Fay, 1950) implemented via the *interactions* package in R (Long, 2019). For informative purposes, these analyses are presented even for the interaction effects that were initially significant but stopped being significant after the false discovery rate (FDR) correction (Benjamini and Hochberg, 1995) was applied.

*General Distancing*

Table S36

*Multiple Linear Regression for the Influence of the Interactions Between the Intervention Conditions and Economic Reasons on General Distancing*

|  | | | | | | **95% CI for *b*** | |
| --- | --- | --- | --- | --- | --- | --- | --- |
| **Condition** | ***b*** | **Std. Error** | ***β*** | ***t*** | ***p*** | **Lower** | **Upper** |
| (Constant) | 4.937 | 0.046 |  | 106.224 | <.001 | 4.845 | 5.028 |
| Letter | -0.100 | 0.069 | -0.064 | -1.451 | .147 | -0.235 | 0.035 |
| Meaningful Activity | -0.071 | 0.067 | -0.047 | -1.063 | .288 | -0.203 | 0.060 |
| Economy | -0.084 | 0.066 | -0.056 | -1.269 | .205 | -0.214 | 0.046 |
| Information | -0.055 | 0.066 | -0.037 | -0.834 | .404 | -0.185 | 0.075 |
| Economic Reasons | -0.104 | 0.017 | -0.253 | -6.024 | <.001 | -0.138 | -0.070 |
| Int. 1 | 0.035 | 0.025 | 0.062 | 1.376 | .169 | -0.015 | 0.085 |
| Int. 2 | 0.033 | 0.024 | 0.064 | 1.356 | .175 | -0.015 | 0.080 |
| Int. 3 | 0.032 | 0.025 | 0.059 | 1.293 | .196 | -0.017 | 0.081 |
| Int. 4 | 0.035 | 0.025 | 0.065 | 1.407 | .160 | -0.014 | 0.083 |
| *Note*. Model *R*^2^ = 0.037. Control condition is the reference category. Int. 1 = Interaction between *Letter* and *Economic Reasons*; Int. 2 = Interaction between *Meaningful Activity* and *Economic Reasons*; Int. 3 = Interaction between *Economy* and *Economic Reasons*; Int. 4 = Interaction between *Information* and *Economic Reasons*. | | | | | | | |

*Note*: Johnson-Neyman analysis was not conducted for *General Distancing* because no significant interaction effects were identified.

*Going Out Times*

Table S37

*Multiple Linear Regression for the Influence of the Interactions Between the Intervention Conditions and Economic Reasons on Going Out Times*

|  | | | | | | **95% CI for *b*** | |
| --- | --- | --- | --- | --- | --- | --- | --- |
| **Condition** | ***b*** | **Std. Error** | ***β*** | ***t*** | ***p*** | **Lower** | **Upper** |
| (Constant) | 0.228 | 0.062 |  | 3.661 | <.001 | 0.106 | 0.350 |
| Letter | 0.016 | 0.092 | 0.008 | 0.173 | .862 | -0.165 | 0.197 |
| Meaningful Activity | 0.166 | 0.090 | 0.083 | 1.846 | .065 | -0.010 | 0.342 |
| Economy | 0.056 | 0.089 | 0.028 | 0.628 | .530 | -0.118 | 0.230 |
| Information | 0.097 | 0.089 | 0.049 | 1.094 | .274 | -0.077 | 0.272 |
| Economic Reasons | 0.089 | 0.023 | 0.164 | 3.848 | <.001 | 0.044 | 0.134 |
| Int. 1 | -0.039 | 0.034 | -0.053 | -1.145 | .252 | -0.106 | 0.028 |
| Int. 2 | -0.093 | 0.032 | -0.136 | -2.858 | .004† | -0.156 | -0.029 |
| Int. 3 | -0.052 | 0.033 | -0.072 | -1.553 | .120 | -0.117 | 0.014 |
| Int. 4 | -0.062 | 0.033 | -0.088 | -1.880 | .060 | -0.126 | 0.003 |
| *Note*. Model *R*^2^ = 0.009. Control condition is the reference category. Symbol † indicates the initially significant interaction effects that stopped being significant after the false discovery rate (FDR) correction was applied. Int. 1 = Interaction between *Letter* and *Economic Reasons*; Int. 2 = Interaction between *Meaningful Activity* and *Economic Reasons*; Int. 3 = Interaction between *Economy* and *Economic Reasons*; Int. 4 = Interaction between *Information* and *Economic Reasons*. | | | | | | | |

Output of the Johnson-Neyman Analysis probing the pattern of the Interaction Between the *Meaningful Activity* Condition and *Economic Reasons*

**JOHNSON-NEYMAN INTERVAL**

When Economic Reasons is above 3.01938321, the Meaningful Activity (vs. Control) condition has a negative influence on Going Out Times (p < .05). When Economic Reasons is below 3.01938321, the Meaningful Activity (vs. Control) condition does not influence Going Out Times (p > .05).

*Note: The range of observed values of Economic Reasons is* [1.00000000, 7.00000000]

Interval calculated using false discovery rate adjusted t = 2.13118090

The figure below corresponds to the visual depiction of the results regarding the pattern of the interaction obtained via the Johnson-Neyman analysis.


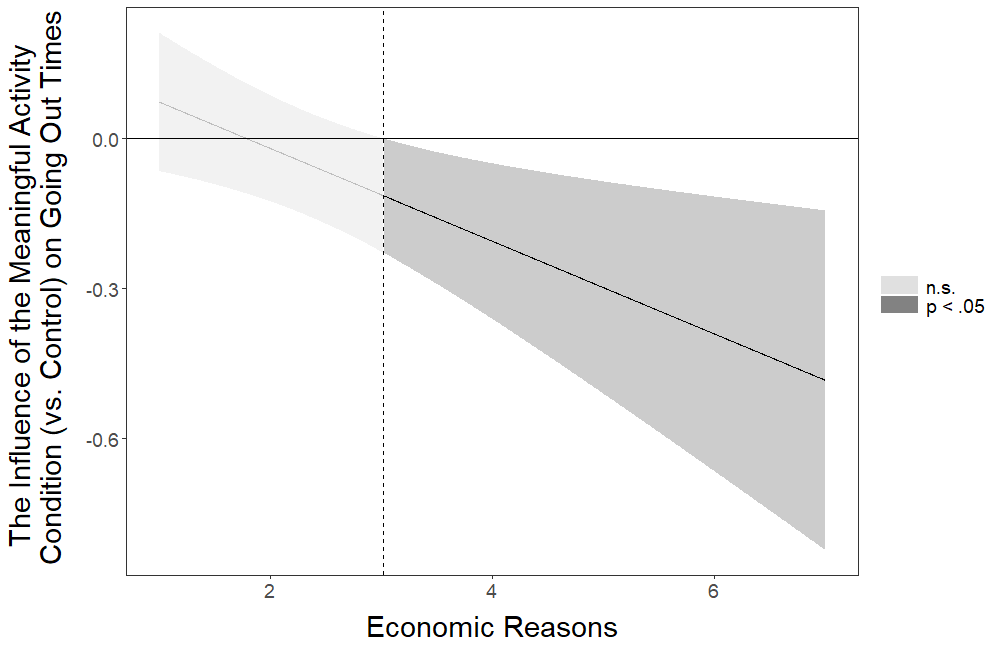


*Figure 17S.* The influence of the meaningful activity (vs. control) condition on going out times at different levels of economic reasons. Mean value of economic reasons is 2.290.

*Going Out Hours*

Table S38

*Multiple Linear Regression for the Influence of the Interactions Between the Intervention Conditions and Economic Reasons on Going Out Hours*

|  | | | | | | **95% CI for *b*** | |
| --- | --- | --- | --- | --- | --- | --- | --- |
| **Condition** | ***b*** | **Std. Error** | ***β*** | ***t*** | ***p*** | **Lower** | **Upper** |
| (Constant) | 0.208 | 0.077 |  | 2.707 | .007 | .057 | .358 |
| Letter | 0.051 | 0.113 | 0.020 | 0.451 | .652 | -.171 | .273 |
| Meaningful Activity | 0.164 | 0.110 | 0.067 | 1.486 | .138 | -.053 | .381 |
| Economy | 0.032 | 0.109 | 0.013 | 0.289 | .772 | -.183 | .246 |
| Information | 0.115 | 0.109 | 0.047 | 1.054 | .292 | -.099 | .330 |
| Economic Reasons | 0.129 | 0.028 | 0.192 | 4.523 | <.001 | .073 | .184 |
| Int. 1 | -0.079 | 0.042 | -0.086 | -1.874 | .061 | -.161 | .004 |
| Int. 2 | -0.100 | 0.040 | -0.119 | -2.507 | .012† | -.178 | -.022 |
| Int. 3 | -0.042 | 0.041 | -0.048 | -1.032 | .302 | -.122 | .038 |
| Int. 4 | -0.094 | 0.040 | -0.108 | -2.322 | .020† | -.173 | -.015 |
| *Note*. Model *R*^2^ = 0.015. Control condition is the reference category. Symbol † indicates the initially significant interaction effects that stopped being significant after the false discovery rate (FDR) correction was applied. Int. 1 = Interaction between *Letter* and *Economic Reasons*; Int. 2 = Interaction between *Meaningful Activity* and *Economic Reasons*; Int. 3 = Interaction between *Economy* and *Economic Reasons*; Int. 4 = Interaction between *Information* and *Economic Reasons*. | | | | | | | |

Output of the Johnson-Neyman Analysis probing the pattern of the Interaction Between the *Meaningful Activity* Condition and *Economic Reasons*

**JOHNSON-NEYMAN INTERVAL**

When Economic Reasons is above 3.06386897, the Meaningful Activity (vs. Control) condition has a negative influence on Going Out Hours (p < .05). When Economic Reasons is below 3.06386897, the Meaningful Activity (vs. Control) condition does not influence Going Out Hours (p > .05).

*Note: The range of observed values of Economic Reasons is* [1.00000000, 7.00000000]

Interval calculated using false discovery rate adjusted t = 2.13544175

The figure below corresponds to the visual depiction of the results regarding the pattern of the interaction obtained via the Johnson-Neyman analysis.


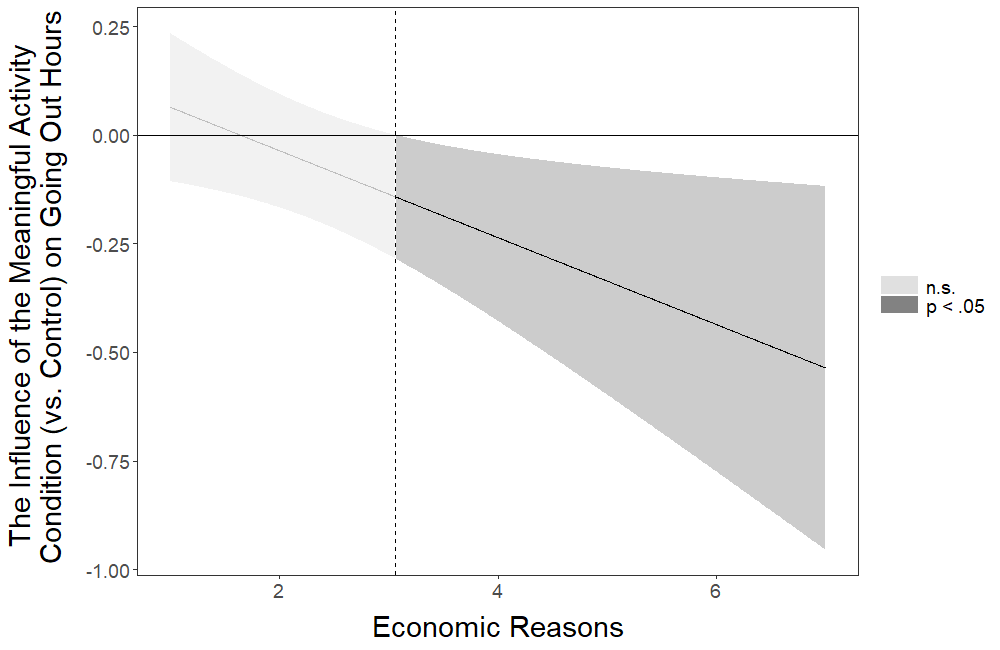


*Figure 18S.* The influence of the meaningful activity (vs. control) condition on going out hours at different levels of economic reasons. Mean value of economic reasons is 2.290.

Output of the Johnson-Neyman Analysis probing the pattern of the Interaction Between the *Information* Condition and *Economic Reasons*

**JOHNSON-NEYMAN INTERVAL**

When Economic Reasons is above 2.56937989, the Information (vs. Control) condition has a negative influence on Going Out Hours (p < .05). When Economic Reasons is below 2.56937989, the Information (vs. Control) condition does not influence Going Out Hours (p > .05).

*Note: The range of observed values of Economic Reasons is* [1.00000000, 7.00000000]

Interval calculated using false discovery rate adjusted t = 2.08778787

The figure below corresponds to the visual depiction of the results regarding the pattern of the interaction obtained via the Johnson-Neyman analysis.


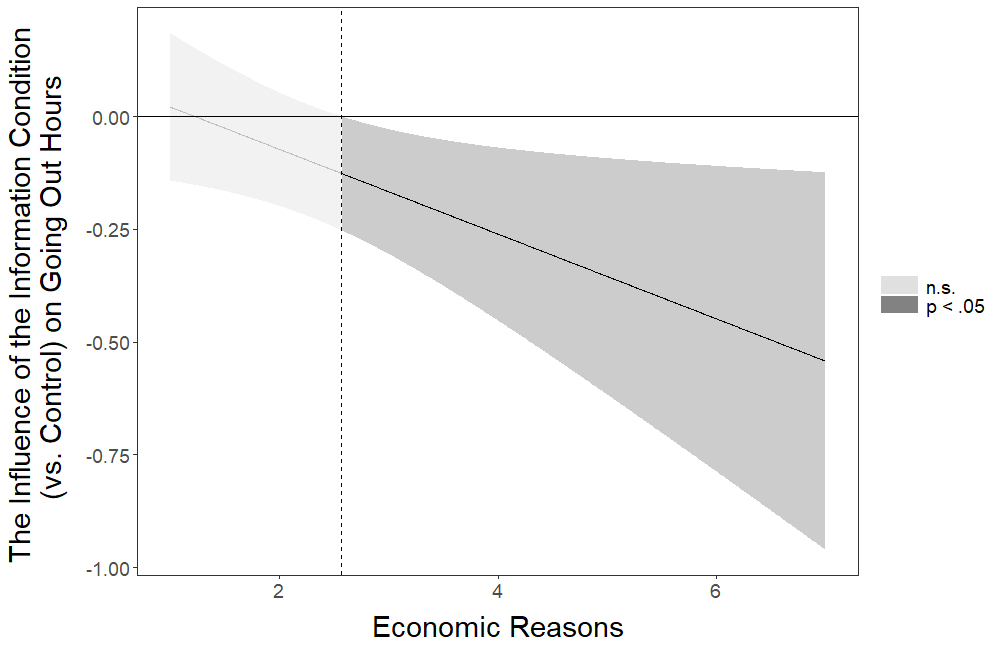


*Figure 19S.* The influence of the information (vs. control) condition on going out hours at different levels of economic reasons. Mean value of economic reasons is 2.290.

*Physical Fitness Times*

Table S39

*Multiple Linear Regression for the Influence of the Interactions Between the Intervention Conditions and Economic Reasons on Physical Fitness Times*

|  | | | | | | **95% CI for *b*** | |
| --- | --- | --- | --- | --- | --- | --- | --- |
| **Condition** | ***b*** | **Std. Error** | ***β*** | ***t*** | ***p*** | **Lower** | **Upper** |
| (Constant) | 0.463 | 0.057 |  | 8.094 | <.001 | 0.351 | 0.575 |
| Letter | -0.062 | 0.085 | -0.033 | -0.735 | .462 | -0.228 | 0.104 |
| Meaningful Activity | -0.059 | 0.082 | -0.032 | -0.719 | .472 | -0.221 | 0.102 |
| Economy | -0.120 | 0.082 | -0.066 | -1.465 | .143 | -0.280 | 0.040 |
| Information | 0.109 | 0.082 | 0.060 | 1.330 | .184 | -0.051 | 0.269 |
| Economic Reasons | 0.013 | 0.021 | 0.026 | 0.616 | .538 | -0.029 | 0.055 |
| Int. 1 | 0.009 | 0.031 | 0.014 | 0.295 | .768 | -0.052 | 0.071 |
| Int. 2 | 0.005 | 0.030 | 0.008 | 0.174 | .862 | -0.053 | 0.064 |
| Int. 3 | 0.017 | 0.030 | 0.027 | 0.573 | .567 | -0.042 | 0.077 |
| Int. 4 | -0.056 | 0.030 | -0.087 | -1.859 | .063 | -0.115 | 0.003 |
| *Note*. Model *R*^2^ = 0.004. Control condition is the reference category. Int. 1 = Interaction between *Letter* and *Economic Reasons*; Int. 2 = Interaction between *Meaningful Activity* and *Economic Reasons*; Int. 3 = Interaction between *Economy* and *Economic Reasons*; Int. 4 = Interaction between *Information* and *Economic Reasons*. | | | | | | | |

*Note*: Johnson-Neyman analysis was not conducted for *Physical Fitness Times* because no significant interaction effects were identified.

*Physical Fitness Hours*

Table S40

*Multiple Linear Regression for the Influence of the Interactions Between the Intervention Conditions and Economic Reasons on Physical Fitness Hours*

|  | | | | | | **95% CI for *b*** | |
| --- | --- | --- | --- | --- | --- | --- | --- |
| **Condition** | ***b*** | **Std. Error** | ***β*** | ***t*** | ***p*** | **Lower** | **Upper** |
| (Constant) | 0.496 | 0.055 |  | 8.981 | <.001 | 0.388 | 0.604 |
| Letter | -0.094 | 0.082 | -0.051 | -1.153 | .249 | -0.254 | 0.066 |
| Meaningful Activity | -0.055 | 0.080 | -0.031 | -0.690 | .490 | -0.211 | 0.101 |
| Economy | -0.105 | 0.079 | -0.060 | -1.332 | .183 | -0.259 | 0.050 |
| Information | 0.054 | 0.079 | 0.031 | 0.684 | .494 | -0.101 | 0.208 |
| Economic Reasons | 0.003 | 0.020 | 0.007 | 0.164 | .870 | -0.037 | 0.044 |
| Int. 1 | 0.020 | 0.030 | 0.031 | 0.675 | .500 | -0.039 | 0.080 |
| Int. 2 | 0.008 | 0.029 | 0.013 | 0.282 | .778 | -0.048 | 0.065 |
| Int. 3 | 0.020 | 0.029 | 0.032 | 0.681 | .496 | -0.038 | 0.078 |
| Int. 4 | -0.052 | 0.029 | -0.084 | -1.789 | .074 | -0.109 | 0.005 |
| *Note*. Model *R*^2^ = 0.004. Control condition is the reference category. Int. 1 = Interaction between *Letter* and *Economic Reasons*; Int. 2 = Interaction between *Meaningful Activity* and *Economic Reasons*; Int. 3 = Interaction between *Economy* and *Economic Reasons*; Int. 4 = Interaction between *Information* and *Economic Reasons*. | | | | | | | |

*Note*: Johnson-Neyman analysis was not conducted for *Physical Fitness Hours* because no significant interaction effects were identified.

*Out Family Friends*

Table S41

*Multiple Logistic Regression for the Influence of the Interactions Between the Intervention Conditions and Economic Reasons on Out Family Friends*

|  | | | | | | **95% CI for Odds Ratio** | |
| --- | --- | --- | --- | --- | --- | --- | --- |
| **Condition** | ***b*** | **Std. Error** | **Wald** | ***p*** | **Odds Ratio** | **Lower** | **Upper** |
| (Constant) | -4.133 | 0.433 | 91.075 | <.001 | 0.016 |  |  |
| Letter | 0.734 | 0.642 | 1.309 | .252 | 2.084 | 0.592 | 7.333 |
| Meaningful Activity | -0.141 | 0.655 | 0.046 | .829 | 0.868 | 0.241 | 3.132 |
| Economy | 1.195 | 0.587 | 4.145 | .042 | 3.302 | 1.046 | 10.428 |
| Information | -0.682 | 0.689 | 0.979 | .322 | 0.506 | 0.131 | 1.952 |
| Economic Reasons | 0.357 | 0.119 | 9.059 | .003 | 1.429 | 1.132 | 1.802 |
| Int. 1 | -0.341 | 0.210 | 2.648 | .104 | 0.711 | 0.471 | 1.072 |
| Int. 2 | -0.024 | 0.176 | 0.018 | .893 | 0.977 | 0.691 | 1.379 |
| Int. 3 | -0.436 | 0.199 | 4.824 | .028† | 0.646 | 0.438 | 0.954 |
| Int. 4 | 0.104 | 0.178 | 0.339 | .561 | 1.109 | 0.782 | 1.574 |
| *Note*. Model *R*^2^ = 0.038 (Nagelkerke), Model *χ*^2^ (9) = 26.930, *p* = .001. Control condition is the reference category. Symbol † indicates the initially significant interaction effects that stopped being significant after the false discovery rate (FDR) correction was applied. Int. 1 = Interaction between *Letter* and *Economic Reasons*; Int. 2 = Interaction between *Meaningful Activity* and *Economic Reasons*; Int. 3 = Interaction between *Economy* and *Economic Reasons*; Int. 4 = Interaction between *Information* and *Economic Reasons*. | | | | | | | |

Output of the Johnson-Neyman Analysis probing the pattern of the Interaction Between the *Economy* Condition and *Economic Reasons*

**JOHNSON-NEYMAN INTERVAL**

The influence of the Economy (vs. Control) condition on Out Family Friends is not significant (p > .05) for any levels of Economic Reasons.

Interval calculated using false discovery rate adjusted t = 4.06260582

The figure below corresponds to the visual depiction of the results regarding the pattern of the interaction obtained via the Johnson-Neyman analysis.


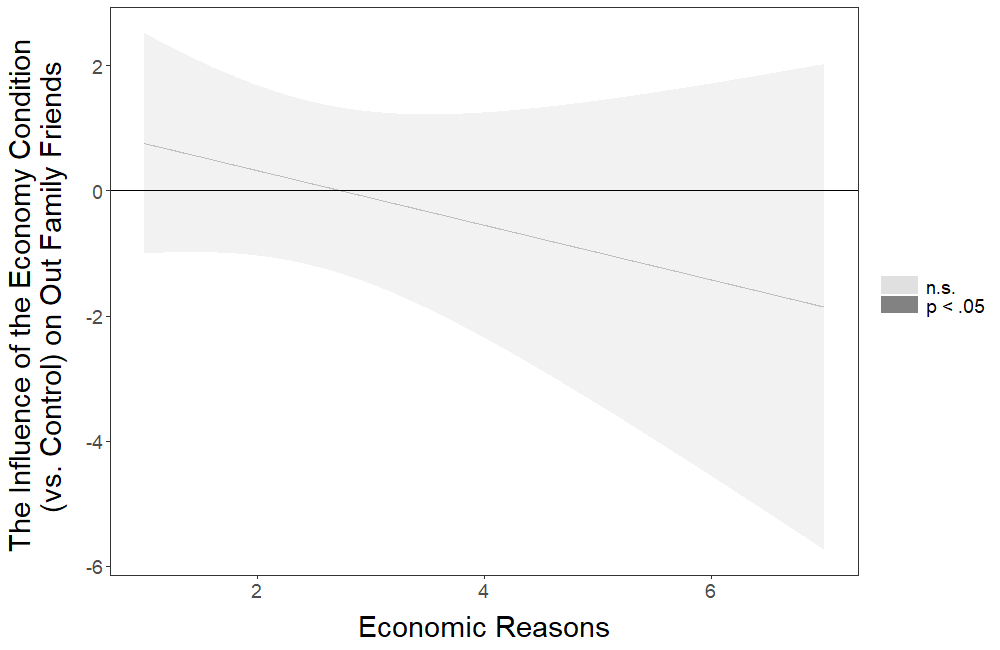


*Figure 20S.* The influence of the economy (vs. control) condition on out family friends at different levels of economic reasons. Mean value of economic reasons is 2.290.

*Social Gatherings*

Table S42

*Multiple Logistic Regression for the Influence of the Interactions Between the Intervention Conditions and Economic Reasons on Social Gatherings*

|  | | | | | | **95% CI for Odds Ratio** | |
| --- | --- | --- | --- | --- | --- | --- | --- |
| **Condition** | ***b*** | **Std. Error** | **Wald** | ***p*** | **Odds Ratio** | **Lower** | **Upper** |
| (Constant) | -3.548 | 0.412 | 74.080 | <.001 | 0.029 |  |  |
| Letter | -0.742 | 0.670 | 1.226 | .268 | 0.476 | 0.128 | 1.771 |
| Meaningful Activity | -0.797 | 0.735 | 1.175 | .278 | 0.451 | 0.107 | 1.904 |
| Economy | -0.218 | 0.561 | 0.151 | .697 | 0.804 | 0.268 | 2.415 |
| Information | -0.821 | 0.750 | 1.200 | .273 | 0.440 | 0.101 | 1.913 |
| Economic Reasons | 0.134 | 0.136 | 0.969 | .325 | 1.143 | 0.876 | 1.491 |
| Int. 1 | 0.166 | 0.202 | 0.678 | .410 | 1.181 | 0.795 | 1.754 |
| Int. 2 | 0.023 | 0.230 | 0.010 | .920 | 1.023 | 0.652 | 1.606 |
| Int. 3 | 0.199 | 0.174 | 1.304 | .253 | 1.220 | 0.867 | 1.717 |
| Int. 4 | -0.017 | 0.248 | 0.005 | .946 | 0.983 | 0.605 | 1.598 |
| *Note*. Model *R*^2^ = 0.045 (Nagelkerke), Model *χ*^2^ (9) = 28.715, *p* = .001. Control condition is the reference category. Int. 1 = Interaction between *Letter* and *Economic Reasons*; Int. 2 = Interaction between *Meaningful Activity* and *Economic Reasons*; Int. 3 = Interaction between *Economy* and *Economic Reasons*; Int. 4 = Interaction between *Information* and *Economic Reasons*. | | | | | | | |

*Note*: Johnson-Neyman analysis was not conducted for *Social Gatherings* because no significant interaction effects were identified.

*Keeping Distance*

Table S43

*Multiple Linear Regression for the Influence of the Interactions Between the Intervention Conditions and Economic Reasons on Keeping Distance*

|  | | | | | | **95% CI for *b*** | |
| --- | --- | --- | --- | --- | --- | --- | --- |
| **Condition** | ***b*** | **Std. Error** | ***β*** | ***t*** | ***p*** | **Lower** | **Upper** |
| (Constant) | 6.886 | 0.095 |  | 72.800 | <.001 | 6.701 | 7.072 |
| Letter | -0.246 | 0.142 | -0.098 | -1.735 | .083 | -0.525 | 0.032 |
| Meaningful Activity | -0.161 | 0.139 | -0.066 | -1.151 | .250 | -0.434 | 0.113 |
| Economy | -0.159 | 0.138 | -0.064 | -1.151 | .250 | -0.430 | 0.112 |
| Information | -0.028 | 0.139 | -0.012 | -0.203 | .839 | -0.300 | 0.244 |
| Economic Reasons | -0.164 | 0.034 | -0.254 | -4.898 | <.001 | -0.230 | -0.098 |
| Int. 1 | 0.102 | 0.050 | 0.117 | 2.022 | .043† | 0.003 | 0.201 |
| Int. 2 | 0.047 | 0.048 | 0.060 | 0.979 | .328 | -0.047 | 0.141 |
| Int. 3 | 0.035 | 0.050 | 0.040 | 0.699 | .485 | -0.063 | 0.133 |
| Int. 4 | 0.040 | 0.050 | 0.046 | 0.787 | .432 | -0.059 | 0.138 |
| *Note*. Model *R*^2^ = 0.041. Control condition is the reference category. Symbol † indicates the initially significant interaction effects that stopped being significant after the false discovery rate (FDR) correction was applied. Int. 1 = Interaction between *Letter* and *Economic Reasons*; Int. 2 = Interaction between *Meaningful Activity* and *Economic Reasons*; Int. 3 = Interaction between *Economy* and *Economic Reasons*; Int. 4 = Interaction between *Information* and *Economic Reasons*. | | | | | | | |

Output of the Johnson-Neyman Analysis probing the pattern of the Interaction Between the *Letter* Condition and *Economic Reasons*

**JOHNSON-NEYMAN INTERVAL**

The influence of the Letter (vs. Control) condition on Keeping Distance is not significant (p > .05) for any levels of Economic Reasons.

Interval calculated using false discovery rate adjusted t = 4.06718718

The figure below corresponds to the visual depiction of the results regarding the pattern of the interaction obtained via the Johnson-Neyman analysis.


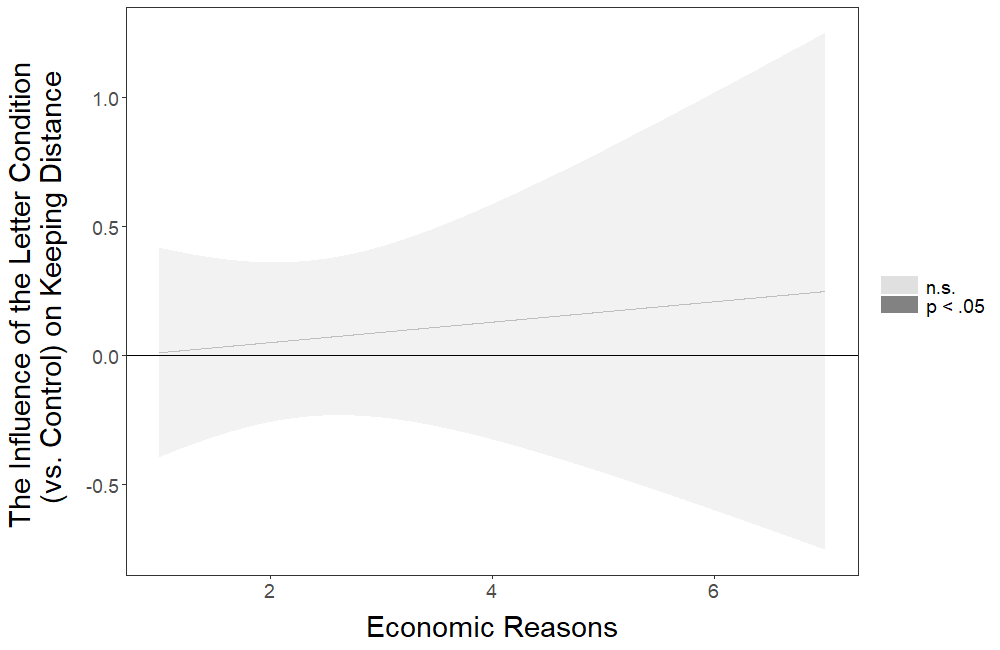


*Figure 21S.* The influence of the letter (vs. control) condition on keeping distance at different levels of economic reasons. Mean value of economic reasons is 2.290.

*Relative Hand Washing*

Table S44

*Multiple Linear Regression for the Influence of the Interactions Between the Intervention Conditions and Economic Reasons on Relative Hand Washing*

|  | | | | | | **95% CI for *b*** | |
| --- | --- | --- | --- | --- | --- | --- | --- |
| **Condition** | ***b*** | **Std. Error** | ***β*** | ***t*** | ***p*** | **Lower** | **Upper** |
| (Constant) | 6.044 | 0.112 |  | 54.067 | <0.001 | 5.825 | 6.263 |
| Letter | -0.185 | 0.165 | -0.050 | -1.119 | 0.263 | -0.509 | 0.139 |
| Meaningful Activity | -0.261 | 0.161 | -0.073 | -1.621 | 0.105 | -0.577 | 0.055 |
| Economy | -0.188 | 0.159 | -0.053 | -1.180 | 0.238 | -0.501 | 0.124 |
| Information | 0.098 | 0.159 | 0.028 | 0.616 | 0.538 | -0.214 | 0.411 |
| Economic Reasons | -0.113 | 0.041 | -0.116 | -2.717 | 0.007 | -0.194 | -0.031 |
| Int. 1 | 0.060 | 0.061 | 0.045 | 0.979 | 0.328 | -0.060 | 0.180 |
| Int. 2 | 0.078 | 0.058 | 0.064 | 1.336 | 0.182 | -0.036 | 0.192 |
| Int. 3 | 0.032 | 0.060 | 0.025 | 0.537 | 0.591 | -0.085 | 0.149 |
| Int. 4 | -0.028 | 0.059 | -0.022 | -0.479 | 0.632 | -0.144 | 0.087 |
| *Note*. Model *R*^2^ = 0.011. Control condition is the reference category. Int. 1 = Interaction between *Letter* and *Economic Reasons*; Int. 2 = Interaction between *Meaningful Activity* and *Economic Reasons*; Int. 3 = Interaction between *Economy* and *Economic Reasons*; Int. 4 = Interaction between *Information* and *Economic Reasons*. | | | | | | | |

*Note*: Johnson-Neyman analysis was not conducted for *Relative Hand Washing* because no significant interaction effects were identified.

*Disinfect*

Table S45

*Multiple Linear Regression for the Influence of the Interactions Between the Intervention Conditions and Economic Reasons on Disinfect*

|  | | | | | | **95% CI for *b*** | |
| --- | --- | --- | --- | --- | --- | --- | --- |
| **Condition** | ***b*** | **Std. Error** | ***β*** | ***t*** | ***p*** | **Lower** | **Upper** |
| (Constant) | 4.579 | 0.203 |  | 22.561 | <.001 | 4.181 | 4.977 |
| Letter | 0.058 | 0.298 | 0.011 | 0.196 | .844 | -0.526 | 0.643 |
| Meaningful Activity | 0.225 | 0.295 | 0.042 | 0.761 | .447 | -0.354 | 0.803 |
| Economy | 0.218 | 0.292 | 0.041 | 0.746 | .456 | -0.355 | 0.791 |
| Information | 0.160 | 0.297 | 0.030 | 0.540 | .590 | -0.422 | 0.742 |
| Economic Reasons | -0.074 | 0.074 | -0.052 | -1.000 | .318 | -0.220 | 0.072 |
| Int. 1 | 0.009 | 0.108 | 0.005 | 0.087 | .931 | -0.203 | 0.222 |
| Int. 2 | 0.012 | 0.103 | 0.007 | 0.113 | .910 | -0.191 | 0.215 |
| Int. 3 | -0.013 | 0.108 | -0.007 | -0.119 | .905 | -0.224 | 0.198 |
| Int. 4 | 0.009 | 0.109 | 0.005 | 0.083 | .934 | -0.204 | 0.222 |
| *Note*. Model *R*^2^ = 0.004. Control condition is the reference category. Int. 1 = Interaction between *Letter* and *Economic Reasons*; Int. 2 = Interaction between *Meaningful Activity* and *Economic Reasons*; Int. 3 = Interaction between *Economy* and *Economic Reasons*; Int. 4 = Interaction between *Information* and *Economic Reasons*. | | | | | | | |

*Note*: Johnson-Neyman analysis was not conducted for *Disinfect* because no significant interaction effects were identified.

*Hand Washing Times*

Table S46

*Multiple Linear Regression for the Influence of the Interactions Between the Intervention Conditions and Economic Reasons on Hand Washing Times*

|  | | | | | | **95% CI for *b*** | |
| --- | --- | --- | --- | --- | --- | --- | --- |
| **Condition** | ***b*** | **Std. Error** | ***β*** | ***t*** | ***p*** | **Lower** | **Upper** |
| (Constant) | 9.639 | 0.368 |  | 26.213 | <.001 | 8.918 | 10.360 |
| Letter | -0.434 | 0.544 | -0.036 | -0.798 | .425 | -1.501 | 0.632 |
| Meaningful Activity | -1.020 | 0.530 | -0.087 | -1.925 | .054 | -2.060 | 0.019 |
| Economy | -0.139 | 0.524 | -0.012 | -0.264 | .792 | -1.167 | 0.890 |
| Information | -0.012 | 0.525 | -0.001 | -0.022 | .982 | -1.040 | 1.017 |
| Economic Reasons | -0.301 | 0.136 | -0.094 | -2.210 | .027 | -0.569 | -0.034 |
| Int. 1 | 0.090 | 0.202 | 0.021 | 0.448 | .654 | -0.305 | 0.486 |
| Int. 2 | 0.351 | 0.192 | 0.088 | 1.832 | .067 | -0.025 | 0.726 |
| Int. 3 | 0.079 | 0.196 | 0.019 | 0.403 | .687 | -0.305 | 0.463 |
| Int. 4 | 0.115 | 0.194 | 0.028 | 0.592 | .554 | -0.266 | 0.496 |
| *Note*. Model *R*^2^ = 0.006. Control condition is the reference category. Int. 1 = Interaction between *Letter* and *Economic Reasons*; Int. 2 = Interaction between *Meaningful Activity* and *Economic Reasons*; Int. 3 = Interaction between *Economy* and *Economic Reasons*; Int. 4 = Interaction between *Information* and *Economic Reasons*. | | | | | | | |

*Note*: Johnson-Neyman analysis was not conducted for *Hand Washing Times* because no significant interaction effects were identified.

# **Covariate Testing for the Interaction Effects That Remained Significant After the False Discovery Rate (FDR) Correction Was Applied**

Below we present the regression analyses for the three significant interaction effects that remained significant after the false discovery rate (FDR) correction (Benjamini and Hochberg, 1995) was applied (the interaction between the information condition and distancing history for general distancing, between the information condition and distancing history for going out times, and between the information condition and distancing history for going out hours) with covariates added as predictors. We also present the results of the analyses probing the patterns of these three interaction effects using the Johnson-Neyman technique (Johnson and Fay, 1950) implemented via the *interactions* package in R (Long, 2019).

*General Distancing*

Table S47

*Multiple Linear Regression for the Influence of the Interactions Between the Intervention Conditions (vs. Control) and Distancing History on General Distancing with the Covariates Included in the Regression Model*

|  | | | | | | **95% CI for *b*** | |
| --- | --- | --- | --- | --- | --- | --- | --- |
| **Condition** | ***b*** | **Std. Error** | ***β*** | ***t*** | ***p*** | **Lower** | **Upper** |
| (Constant) | 4.064 | 0.130 |  | 31.285 | <.001 | 3.809 | 4.318 |
| Letter | 0.054 | 0.094 | 0.034 | 0.573 | .567 | -0.130 | 0.238 |
| Meaningful Activity | 0.081 | 0.086 | 0.054 | 0.949 | .343 | -0.087 | 0.249 |
| Economy | 0.089 | 0.089 | 0.059 | 0.992 | .321 | -0.087 | 0.264 |
| Information | 0.250 | 0.086 | 0.168 | 2.928 | .003 | 0.083 | 0.418 |
| Distancing History | 0.016 | 0.003 | 0.245 | 5.628 | <.001 | 0.010 | 0.021 |
| Int. 1 | -0.004 | 0.004 | -0.057 | -0.937 | .349 | -0.012 | 0.004 |
| Int. 2 | -0.004 | 0.004 | -0.057 | -0.973 | .330 | -0.011 | 0.004 |
| Int. 3 | -0.005 | 0.004 | -0.077 | -1.252 | .211 | -0.013 | 0.003 |
| Int. 4 | -0.011 | 0.004 | -0.186 | -3.050 | .002 | -0.018 | -0.004 |
| Household Income | 0.026 | 0.012 | 0.044 | 2.256 | .024 | 0.003 | 0.049 |
| Prior Home | -0.009 | 0.005 | -0.036 | -1.736 | .083 | -0.019 | 0.001 |
| Household | -0.004 | 0.009 | -0.010 | -0.457 | .648 | -0.021 | 0.013 |
| Garden | 0.035 | 0.035 | 0.021 | 0.995 | .320 | -0.034 | 0.103 |
| Key Worker | -0.208 | 0.028 | -0.144 | -7.388 | <.001 | -0.264 | -0.153 |
| Age | 0.003 | 0.001 | 0.072 | 3.335 | .001 | 0.001 | 0.004 |
| Female | 0.139 | 0.023 | 0.115 | 6.138 | <.001 | 0.095 | 0.183 |
| Other | 0.145 | 0.259 | 0.010 | 0.561 | .575 | -0.362 | 0.653 |
| Education1 | 0.084 | 0.067 | 0.064 | 1.246 | .213 | -0.048 | 0.215 |
| Education2 | 0.137 | 0.067 | 0.109 | 2.062 | .039 | 0.007 | 0.268 |
| Education3 | 0.084 | 0.069 | 0.058 | 1.215 | .225 | -0.051 | 0.218 |
| Education4 | 0.139 | 0.093 | 0.038 | 1.491 | .136 | -0.044 | 0.321 |
| Education5 | 0.145 | 0.083 | 0.051 | 1.740 | .082 | -0.018 | 0.309 |
| Property1 | 0.106 | 0.116 | 0.023 | 0.910 | .363 | -0.122 | 0.334 |
| Property2 | 0.163 | 0.088 | 0.082 | 1.861 | .063 | -0.009 | 0.335 |
| Property3 | 0.056 | 0.083 | 0.039 | 0.675 | .500 | -0.106 | 0.218 |
| Property4 | 0.082 | 0.082 | 0.066 | 1.001 | .317 | -0.078 | 0.242 |
| Property5 | 0.093 | 0.083 | 0.066 | 1.122 | .262 | -0.070 | 0.257 |
| Country | -0.214 | 0.024 | -0.177 | -9.012 | <.001 | -0.260 | -0.167 |
| On Time | -0.044 | 0.036 | -0.023 | -1.209 | .227 | -0.115 | 0.027 |
| *Note*. Model *R*^2^ = 0.110. For the interventions included in the model, control condition is the reference category. Int. 1 = Interaction between *Letter* and *Distancing History*; Int. 2 = Interaction between *Meaningful Activity* and *Distancing History*; Int. 3 = Interaction between *Economy* and *Distancing History*; Int. 4 = Interaction between *Information* and *Distancing History*. Education 1 = Secondary education; Education 2 = Undergraduate degree; Education 3 = Graduate degree; Education 4 = Doctoral degree; Education 5 = Professional qualification (“No formal qualifications” is the baseline condition for Education). Property1 = Living in a studio flat; Property2 = Living in a one bedroom property; Property3 = Living in a two bedroom property; Property4 = Living in a three bedroom property; Property5 = Living in a property that has more than three bedrooms (“Living in a single room in shared accommodation” is the baseline condition for Property). | | | | | | | |

Output of the Johnson-Neyman Analysis probing the pattern of the Interaction Between the *Information* Condition and *Distancing History*

**JOHNSON-NEYMAN INTERVAL**

When Distancing History is below 14.56049538, the Information (vs. Control) condition has a positive influence on General Distancing (p < .05). When Distancing History is above 31.85325567, the Information (vs. Control) condition has a negative influence on General Distancing (p < .05).

*Note: The range of observed values of Distancing History is* [0.00000000, 101.00000000]

Interval calculated using false discovery rate adjusted t = 2.03992625

The figure below corresponds to the visual depiction of the results regarding the pattern of the interaction obtained via the Johnson-Neyman analysis.


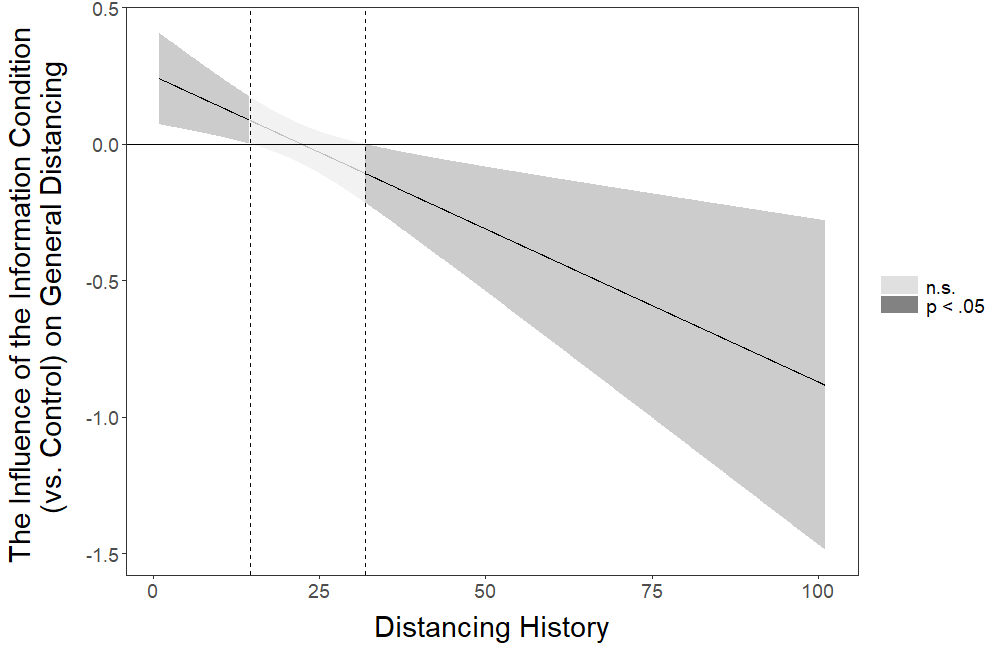


*Figure 22S.* The influence of the information (vs. control) condition on general distancing at different levels of distancing history. Mean value of distancing history is 21.134.

*Going Out Times*

Table S48

*Multiple Linear Regression for the Influence of the Interactions Between the Intervention Conditions (vs. Control) and Distancing History on Going Out Times with the Covariates Included in the Regression Model*

|  | | | | | | **95% CI for *b*** | |
| --- | --- | --- | --- | --- | --- | --- | --- |
| **Condition** | ***b*** | **Std. Error** | ***β*** | ***t*** | ***p*** | **Lower** | **Upper** |
| (Constant) | 0.728 | 0.179 |  | 4.069 | <.001 | 0.377 | 1.079 |
| Letter | -0.249 | 0.129 | -0.120 | -1.926 | .054 | -0.502 | 0.004 |
| Meaningful Activity | -0.197 | 0.118 | -0.098 | -1.671 | .095 | -0.428 | 0.034 |
| Economy | -0.206 | 0.123 | -0.104 | -1.676 | .094 | -0.448 | 0.035 |
| Information | -0.482 | 0.118 | -0.244 | -4.092 | <.001 | -0.713 | -0.251 |
| Distancing History | -0.017 | 0.004 | -0.194 | -4.280 | <.001 | -0.024 | -0.009 |
| Int. 1 | 0.009 | 0.006 | 0.098 | 1.532 | .126 | -0.002 | 0.020 |
| Int. 2 | 0.007 | 0.005 | 0.089 | 1.440 | .150 | -0.003 | 0.018 |
| Int. 3 | 0.007 | 0.005 | 0.086 | 1.346 | .178 | -0.003 | 0.018 |
| Int. 4 | 0.021 | 0.005 | 0.264 | 4.164 | <.001 | 0.011 | 0.031 |
| Household Income | 0.031 | 0.016 | 0.039 | 1.911 | .056 | -0.001 | 0.063 |
| Prior Home | -0.006 | 0.007 | -0.019 | -0.880 | .379 | -0.020 | 0.008 |
| Household | -0.001 | 0.012 | -0.001 | -0.044 | .965 | -0.024 | 0.023 |
| Garden | 0.025 | 0.048 | 0.012 | 0.528 | .598 | -0.069 | 0.120 |
| Key Worker | 0.020 | 0.039 | 0.011 | 0.520 | .603 | -0.056 | 0.096 |
| Age | -0.002 | 0.001 | -0.031 | -1.388 | .165 | -0.004 | 0.001 |
| Female | -0.142 | 0.031 | -0.089 | -4.561 | <.001 | -0.204 | -0.081 |
| Other | -0.207 | 0.357 | -0.011 | -0.580 | .562 | -0.906 | 0.492 |
| Education1 | 0.085 | 0.093 | 0.049 | 0.922 | .357 | -0.096 | 0.267 |
| Education2 | 0.083 | 0.092 | 0.050 | 0.900 | .368 | -0.097 | 0.262 |
| Education3 | 0.174 | 0.095 | 0.092 | 1.839 | .066 | -0.011 | 0.360 |
| Education4 | 0.168 | 0.128 | 0.035 | 1.314 | .189 | -0.083 | 0.419 |
| Education5 | 0.088 | 0.115 | 0.023 | 0.764 | .445 | -0.138 | 0.313 |
| Property1 | -0.047 | 0.160 | -0.008 | -0.295 | .768 | -0.361 | 0.267 |
| Property2 | 0.007 | 0.121 | 0.002 | 0.054 | .957 | -0.230 | 0.243 |
| Property3 | 0.014 | 0.114 | 0.007 | 0.123 | .902 | -0.209 | 0.237 |
| Property4 | -0.044 | 0.112 | -0.027 | -0.392 | .695 | -0.265 | 0.176 |
| Property5 | -0.042 | 0.115 | -0.022 | -0.363 | .717 | -0.266 | 0.183 |
| Country | 0.011 | 0.033 | 0.007 | 0.329 | .742 | -0.053 | 0.075 |
| On Time | 0.017 | 0.050 | 0.007 | 0.339 | .734 | -0.081 | 0.115 |
| *Note*. Model *R*^2^ = 0.032. For the interventions included in the model, control condition is the reference category. Int. 1 = Interaction between *Letter* and *Distancing History*; Int. 2 = Interaction between *Meaningful Activity* and *Distancing History*; Int. 3 = Interaction between *Economy* and *Distancing History*; Int. 4 = Interaction between *Information* and *Distancing History*. Education 1 = Secondary education; Education 2 = Undergraduate degree; Education 3 = Graduate degree; Education 4 = Doctoral degree; Education 5 = Professional qualification (“No formal qualifications” is the baseline condition for Education). Property1 = Living in a studio flat; Property2 = Living in a one bedroom property; Property3 = Living in a two bedroom property; Property4 = Living in a three bedroom property; Property5 = Living in a property that has more than three bedrooms (“Living in a single room in shared accommodation” is the baseline condition for Property). | | | | | | | |

Output of the Johnson-Neyman Analysis probing the pattern of the Interaction Between the *Information* Condition and *Distancing History*

**JOHNSON-NEYMAN INTERVAL**

When Distancing History is below 18.00869779, the Information (vs. Control) condition has a negative influence on Going Out Times (p < .05). When Distancing History is above 28.66272222, the Information (vs. Control) condition has a positive influence on Going Out Times (p < .05).

*Note: The range of observed values of Distancing History is* [0.00000000, 101.00000000]

Interval calculated using false discovery rate adjusted t = 2.00791829

The figure below corresponds to the visual depiction of the results regarding the pattern of the interaction obtained via the Johnson-Neyman analysis.


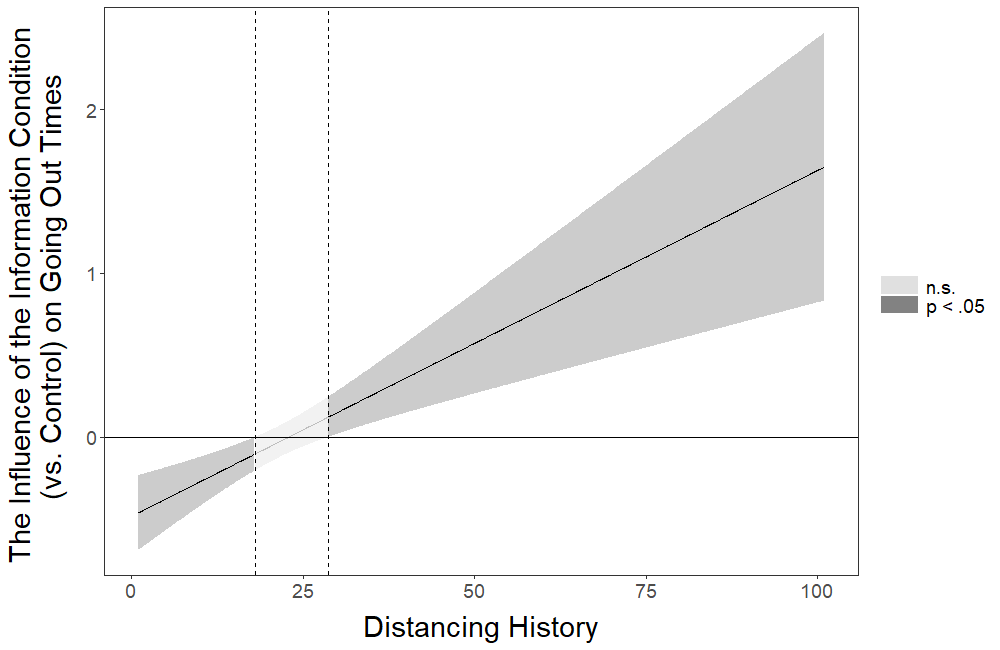


*Figure 23S.* The influence of the information (vs. control) condition on going out times at different levels of distancing history. Mean value of distancing history is 21.134.

*Going Out Hours*

Table S49

*Multiple Linear Regression for the Influence of the Interactions Between the Intervention Conditions (vs. Control) and Distancing History on Going Out Hours with the Covariates Included in the Regression Model*

|  | | | | | | **95% CI for *b*** | |
| --- | --- | --- | --- | --- | --- | --- | --- |
| **Condition** | ***b*** | **Std. Error** | ***β*** | ***t*** | ***p*** | **Lower** | **Upper** |
| (Constant) | 0.767 | 0.221 |  | 3.476 | .001 | 0.334 | 1.200 |
| Letter | -0.467 | 0.159 | -0.183 | -2.932 | .003 | -0.779 | -0.155 |
| Meaningful Activity | -0.288 | 0.145 | -0.117 | -1.983 | .047 | -0.573 | -0.003 |
| Economy | -0.200 | 0.152 | -0.082 | -1.317 | .188 | -0.497 | 0.098 |
| Information | -0.547 | 0.145 | -0.225 | -3.769 | <.001 | -0.832 | -0.263 |
| Distancing History | -0.018 | 0.005 | -0.173 | -3.810 | <.001 | -0.027 | -0.009 |
| Int. 1 | 0.016 | 0.007 | 0.145 | 2.278 | .023† | 0.002 | 0.030 |
| Int. 2 | 0.011 | 0.006 | 0.105 | 1.707 | .088 | -0.002 | 0.023 |
| Int. 3 | 0.007 | 0.007 | 0.064 | 1.011 | .312 | -0.006 | 0.020 |
| Int. 4 | 0.021 | 0.006 | 0.215 | 3.398 | .001 | 0.009 | 0.033 |
| Household Income | 0.007 | 0.020 | 0.007 | 0.345 | .730 | -0.032 | 0.046 |
| Prior Home | -0.007 | 0.009 | -0.018 | -0.851 | .395 | -0.024 | 0.010 |
| Household | 0.004 | 0.015 | 0.006 | 0.274 | .784 | -0.025 | 0.033 |
| Garden | 0.050 | 0.059 | 0.019 | 0.839 | .402 | -0.066 | 0.166 |
| Key Worker | 0.056 | 0.048 | 0.024 | 1.179 | .239 | -0.037 | 0.150 |
| Age | -0.001 | 0.001 | -0.015 | -0.657 | .511 | -0.004 | 0.002 |
| Female | -0.183 | 0.038 | -0.093 | -4.745 | <.001 | -0.258 | -0.107 |
| Other | 1.126 | 0.440 | 0.050 | 2.563 | .010 | 0.265 | 1.988 |
| Education1 | 0.096 | 0.114 | 0.045 | 0.844 | .399 | -0.127 | 0.320 |
| Education2 | 0.107 | 0.113 | 0.052 | 0.946 | .344 | -0.115 | 0.329 |
| Education3 | 0.228 | 0.117 | 0.097 | 1.954 | .051 | -0.001 | 0.457 |
| Education4 | 0.326 | 0.158 | 0.055 | 2.067 | .039 | 0.017 | 0.635 |
| Education5 | 0.160 | 0.142 | 0.034 | 1.131 | .258 | -0.118 | 0.438 |
| Property1 | 0.076 | 0.197 | 0.010 | 0.386 | .699 | -0.311 | 0.463 |
| Property2 | 0.101 | 0.149 | 0.031 | 0.676 | .499 | -0.191 | 0.392 |
| Property3 | 0.073 | 0.140 | 0.031 | 0.518 | .605 | -0.203 | 0.348 |
| Property4 | -0.004 | 0.139 | -0.002 | -0.025 | .980 | -0.275 | 0.268 |
| Property5 | -0.042 | 0.141 | -0.018 | -0.298 | .766 | -0.319 | 0.235 |
| Country | 0.028 | 0.040 | 0.014 | 0.694 | .488 | -0.051 | 0.107 |
| On Time | 0.160 | 0.062 | 0.051 | 2.593 | .010 | 0.039 | 0.282 |
| *Note*. Model *R*^2^ = 0.034. For the interventions included in the model, control condition is the reference category. Symbol † indicates the interaction effect that stopped being significant after the false discovery rate (FDR) correction was applied in the main interaction testing without covariates. Int. 1 = Interaction between *Letter* and *Distancing History*; Int. 2 = Interaction between *Meaningful Activity* and *Distancing History*; Int. 3 = Interaction between *Economy* and *Distancing History*; Int. 4 = Interaction between *Information* and *Distancing History*. Education 1 = Secondary education; Education 2 = Undergraduate degree; Education 3 = Graduate degree; Education 4 = Doctoral degree; Education 5 = Professional qualification (“No formal qualifications” is the baseline condition for Education). Property1 = Living in a studio flat; Property2 = Living in a one bedroom property; Property3 = Living in a two bedroom property; Property4 = Living in a three bedroom property; Property5 = Living in a property that has more than three bedrooms (“Living in a single room in shared accommodation” is the baseline condition for Property). | | | | | | | |

Output of the Johnson-Neyman Analysis probing the pattern of the Interaction Between the *Information* Condition and *Distancing History*

**JOHNSON-NEYMAN INTERVAL**

When Distancing History is below 20.05148987, the Information (vs. Control) condition has a negative influence on Going Out Hours (p < .05). When Distancing History is above 36.64384779, the Information (vs. Control) condition has a positive influence on Going Out Hours (p < .05).

*Note: The range of observed values of Distancing History is* [0.00000000, 101.00000000]

Interval calculated using false discovery rate adjusted t = 2.03643140

The figure below corresponds to the visual depiction of the results regarding the pattern of the interaction obtained via the Johnson-Neyman analysis.


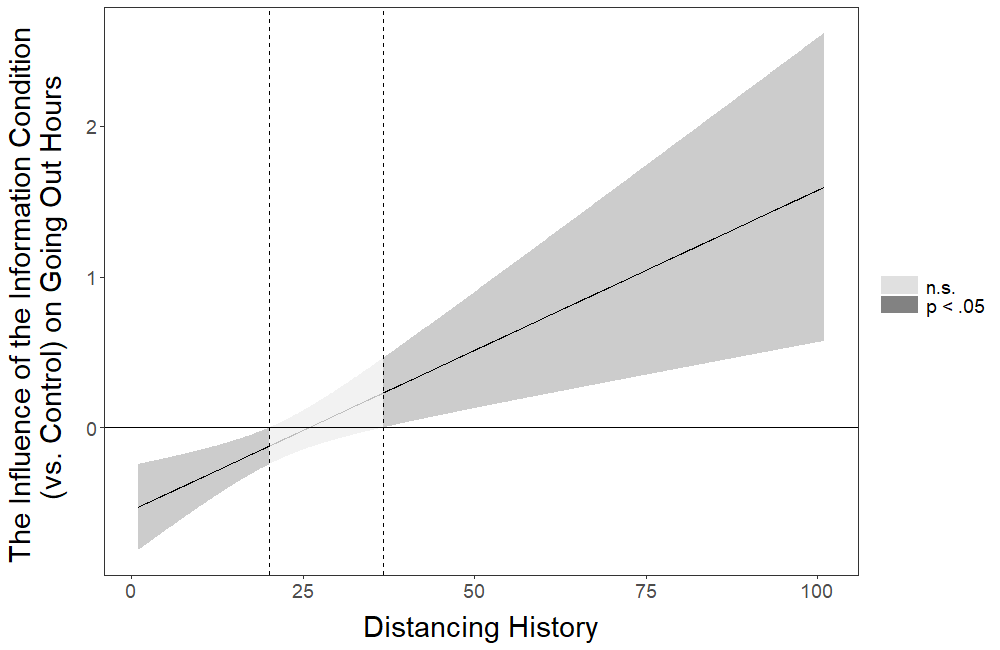


*Figure 24S.* The influence of the information (vs. control) condition on going out hours at different levels of distancing history. Mean value of distancing history is 21.134.

# **References**

Benjamini, Y., and Y. Hochberg (1995), ‘Controlling the false discovery rate: a practical and powerful approach to multiple testing’, *Journal of the Royal statistical society: Series B (Methodological)*, 57(1): 289-300.

Johnson, P.O. and L. C. Fay (1950), ‘The Johnson-Neyman technique, its theory and application. *Psychometrika*’, 15(4): 349-367.

Long, J. A. (2019), *Package ‘interactions’*, Available from: https://cran.rproject.org/web/packages/interactions/interactions.pdf

1. All categorical variables from the present study are labelled with * next to their name. [↑](#footnote-ref-1)
2. This is the original phrasing we used. However, it may be more appropriate to use the phrasing “family members/ friends” instead of “family/ friends members”. [↑](#footnote-ref-2)
3. This is how the original sentence was phrased in the experiment. However, it might be more appropriate to phrase the sentence as follows: “When social distancing is called for by the government going forward, you would still visit facilities that have stepped up their efforts to clean and disinfect their buildings.” [↑](#footnote-ref-3)
4. This item was adopted from the Gallup World Poll and is based on one of the core questions in the domain of “Business and Economics” (Gallup World Poll questions can be obtained via this link: <http://media.gallup.com/dataviz/www/WP_Questions.pdf>) [↑](#footnote-ref-4)
